# Efficacy, safety, and feasibility of youth-derived fecal microbiota transplantation among adults with type 1 diabetes mellitus: A protocol of Pilot Randomized Controlled Trial

**DOI:** 10.1371/journal.pone.0343078

**Published:** 2026-09-21

**Authors:** Xunuo Chen, Mengyun Lei, Jun Tang, Huawei Wang, Jiawei Chen, Yanxin Liu, Shuting Li, Fang Liu, Yonge Wang, Zhiqiang Li, Zhe Dai

**Affiliations:** 1 Department of Endocrinology, Zhongnan Hospital of Wuhan University, Wuhan, China; 2 Department of Clinical Nutrition, Zhongnan Hospital of Wuhan University, Wuhan, China; 3 Department of Neurosurgery, Zhongnan Hospital of Wuhan University, Wuhan, China; 4 State Key Laboratory of Metabolism and Regulation in Complex Organisms, College of Life Sciences, Wuhan University, Wuhan, China; Zhejiang University, CHINA

## Abstract

**Background:**

Dysbiosis of gut microbiota plays a key role in type 1 diabetes mellitus (T1DM). Fecal microbiota transplantation represents a novel therapeutic avenue. We hypothesize that youth-derived fecal microbiota transplantation (yFMT) can remodel the gut microecosystem and improve clinical outcomes. This pilot trial aims to assess the feasibility, safety, and preliminary efficacy of yFMT in adults with T1DM.

**Methods and analysis:**

This single-center, randomized, double-blind, placebo-controlled pilot study will enroll adults with T1DM who have suboptimal glycemic outcomes (glycated hemoglobin [HbA1c] of 7.0–14.0% or time in range [TIR] <70%). Following a 17-day run-in period for insulin optimization, continuous glucose monitoring (CGM) wearing, baseline assessments and bowel preparation, participants will be randomly allocated (1:1) to take yFMT or placebo capsules for 6 consecutive days, alongside their standard insulin therapy, and then complete a 12-week follow-up. The primary efficacy endpoint is the change from baseline in the rate of achieving the composite target of TIR > 70% and time below range <4% at 12 weeks post-randomization. Secondary efficacy endpoints include: (1) the change from baseline in the same composite achievement rate at 4 weeks post-intervention; (2) changes from baseline at Weeks 4 and 12 in other glycemic metrics (including HbA1c, fasting plasma glucose, 2-hour postprandial glucose, and additional CGM metrics), C-peptide, immune responses, infection markers, and gut microbiota composition; and (3) changes from baseline at Week 12 in serum metabolomic profiles (bile acids, short-chain fatty acids, and other related metabolites). Feasibility will be assessed through recruitment rate, retention rate, intervention adherence, and acceptability. Safety endpoints include the incidence of adverse events and serious adverse events.

**Discussion:**

Our findings will offer new insight into the feasibility and effects of oral yFMT capsules in adults with T1DM and provide the necessary evidence to power a subsequent multicenter large-scale study. Exploratory biomarker analyses conducted within this study may further pave the way for future individualized microbiome‑based therapeutics.

**Trial registration:**

Chinese Clinical Trial Registry identifier: ChiCTR2500111955 (November 7, 2025).

## 1. Introduction

Type 1 diabetes mellitus (T1DM) is a chronic autoimmune disease characterized by the destruction of pancreatic β cells and lifelong insulin dependence [1]. Although advances in insulin analogs, delivery modalities, and continuous glucose monitoring (CGM) systems have improved diabetes management, people with T1DM still face challenges such as suboptimal glycemic outcomes, frequent episodes of hypoglycemia, and a high burden of self-management [2–7].

The core pathological mechanism of T1DM involves autoimmune-mediated destruction of pancreatic β cells. Conventional insulin therapy fails to halt the autoimmune process or restore β-cell function. In recent years, the gut microbiota, termed the “second genome,” has been recognized as an important contributor to T1DM pathogenesis [8]. Numerous studies have characterized gut microbiota alterations in individuals with T1DM, marked by a reduction in butyrate-producing bacteria and secondary bile acids, along with an increase in pathobionts [9–11]. Impaired intestinal barrier function represents another hallmark of this dysbiotic state, and together these features contribute to disruption of the “gut microbiota-intestinal-islet axis” [11].

Fecal microbiota transplantation (FMT) is a promising technique that transfers healthy donor microbiota to a recipient, aiming to restore microbial homeostasis. Evidence from animal studies has demonstrated that modulation of the gut microbiota can improve glycemic control through multiple mechanisms, including enhancement of intestinal barrier integrity, increased production of short-chain fatty acids, regulation of immune responses, and preservation of residual β-cell function [12,13].

Clinical evidence supports the potential of FMT in enhancing glycemic control among patients with T1DM. In a randomized controlled trial (RCT), both autologous and allogeneic FMT groups exhibited a reduction in glycated hemoglobin (HbA1c) at 12 months, without significant changes in insulin dosages [14]. Additionally, clinical studies demonstrate that washed microbiota transplantation significantly increases the time in range (TIR) and reduces glycemic variability among adults with T1DM [15,16]. Together with two capsule-based RCTs [17,18], these studies showed that FMT was generally safe, with mild and transient gastrointestinal symptoms being the most frequent adverse events [14–18].

Despite preliminary evidence supporting the potential of FMT to treat T1DM, several challenges remain: (1) studies primarily target β-cell function with inconsistent results, and lack assessment of autoimmunity or insulin sensitivity; (2) reliance on HbA1c or isolated CGM values, while composite target of TIR (3.9–10.0 mmol/L) >70% and time below range <3.9mmol/L (TBR3.9) <4% are seldom used; (3)donor screening lacks unified criteria, and techniques for preparation and delivery vary widely; and (4) the complex and poorly tolerated nasojejunal tube remains the primary administration method.

The youth-derived FMT capsules are prepared from rigorously screened healthy young donors (aged 4–17 years), selected for the relatively high temporal stability of the gut microbiota during this developmental window [19,20]. Our study pioneers the oral use of yFMT in a pilot RCT to evaluate the feasibility, safety, and preliminary efficacy of yFMT in adults with T1DM.

## 2. Methods

### 2.1. Study design

**Fig 1:**
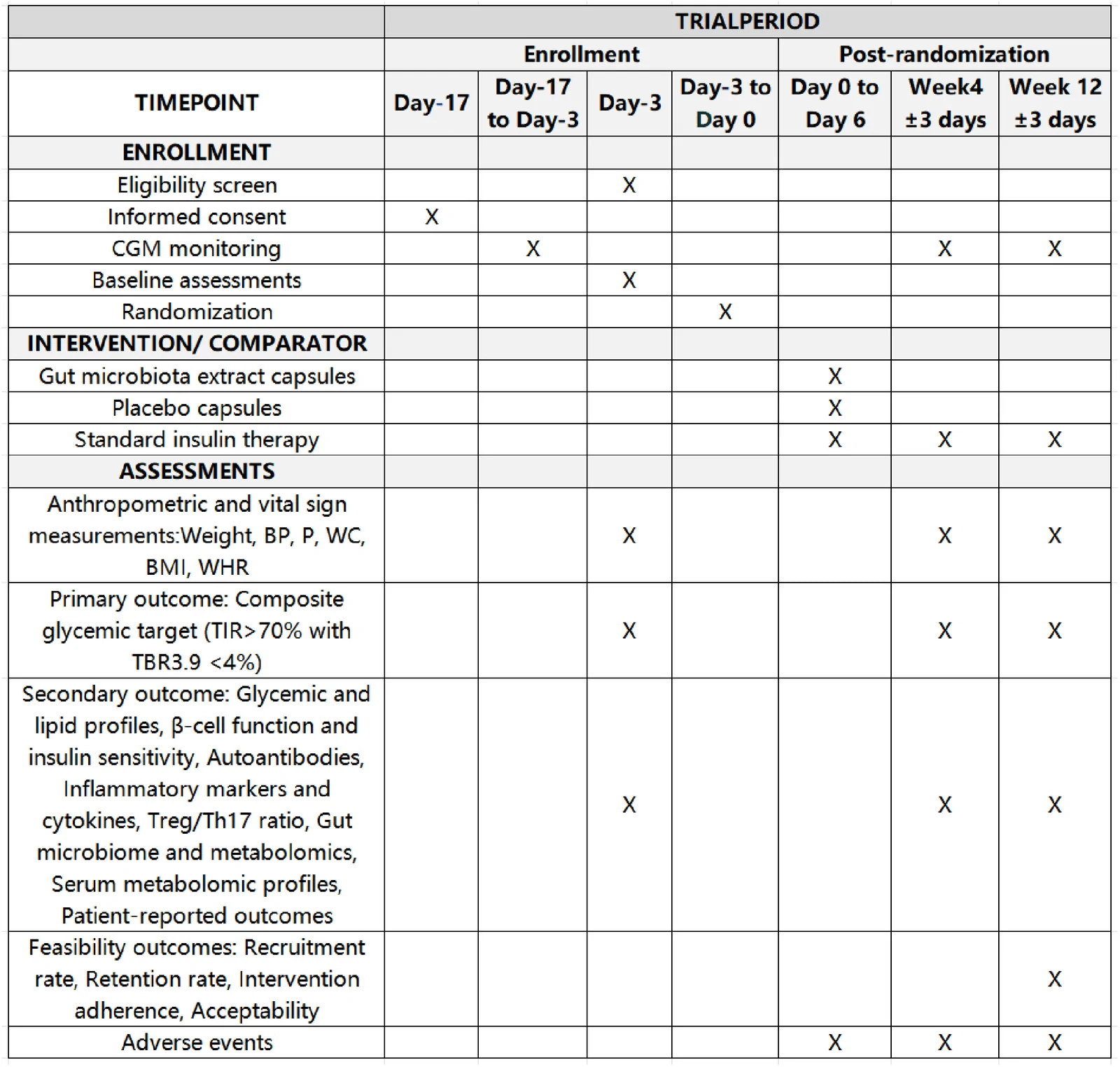
SPIRIT schedule of enrollment, intervention, and assessments. Note: Following informed consent on Day −17, candidates will undergo a 14-day CGM assessment and HbA1c testing (Day −3). Only those with HbA1c of 7.0–14.0% or CGM-derived TIR below 70% will be enrolled. Abbreviations: CGM, Continuous Glucose Monitoring; BP, Blood Pressure; P, Pulse; WC, Waist Circumference; BMI, Body Mass Index; WHR, Waist-to-Hip Ratio; TIR, Time in Range; TBR3.9, Time Below Range <3.9 mmol/L; Treg, Regulatory T cells; Th17, T helper 17 cells;.

This pilot, randomized, double-blind, placebo-controlled trial conducted at Zhongnan Hospital of Wuhan University (China), is designed primarily to assess the efficacy, safety, and feasibility of yFMT in adults with T1DM. The schedule of activities to be performed and a study flowchart are provided in Figs 1 **and** 2. Participants will be randomized in a 1:1 ratio to receive either oral yFMT capsules or placebo capsules. The study will remain blinded until the database lock. Participants will provide written informed consent prior to enrollment.

**Fig 2:**
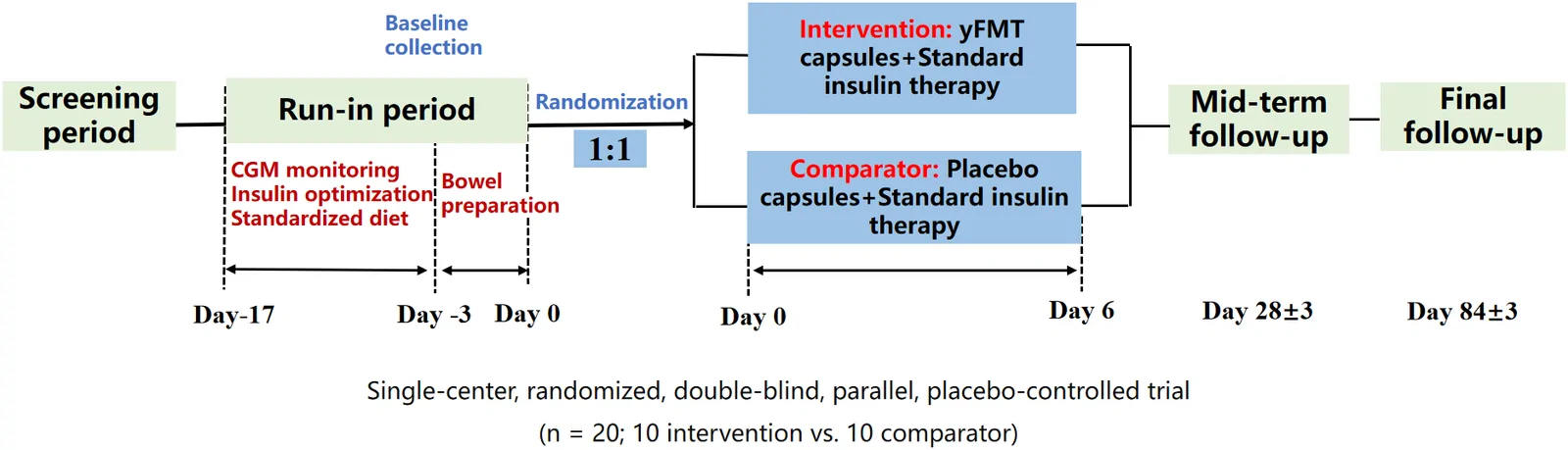
Flow chart of design. Abbreviations: CGM, Continuous Glucose Monitoring; yFMT, youth-derived fecal microbiota transplantation;.

### 2.2. Trial status

This study was approved by the Medical Ethics Committee, Zhongnan Hospital of Wuhan University (Ethics Approval No. 2025302) in October 2025. Recruitment started on November 20, 2025, and is ongoing, with completion expected by July 31, 2026. Data collection will conclude by November 30, 2026, with results expected by January 31, 2027.

### 2.3. Participants

Participant recruitment will be conducted from November 2025 to July 2026. Eligible participants aged 18–60 years must have a T1DM duration of more than one year and suboptimal glycemic outcomes, defined as HbA1c of 7.0–14.0% within the past three months or 14-day CGM-derived TIR < 70% within the past month. Participants will be excluded if they had recent episodes of diabetic ketoacidosis, concurrent severe gastrointestinal diseases, significant gastrointestinal surgery, pregnancy, or lactation. The diagnosis of T1DM adheres to the criteria established by the World Health Organization and the American Diabetes Association. Full criteria are listed in Table 1.

**Table 1:** Inclusion and exclusion criteria for enrollment.

Inclusion Criteria	
1	T1DM was diagnosed by an endocrinologist for at least 1 year
2	Aged 18–60 years (inclusive)
3	HbA1c between 7.0 to 14.0% (within past 3 months) or TIR < 70% (14-day CGM within the past month)
4	On MDI or insulin pump therapy for ≥3 months
5	TDD before admission ≥0.3 IU/kg/day, with a basal rate ≥0.05 IU/hour
6	Women of childbearing age are willing to use appropriate contraceptive measures
7	Signed informed consent form
**Exclusion Criteria**	
1	Treatment with any anti-hyperglycemic medications other than insulin
2	Continuous use of antimicrobial agents, probiotics, intestinal microecological modulators, or other medications with potential impact on gut microbiota for >3 days within 3 months prior to enrollment;
3	Occurrence of acute diabetic complications (diabetic ketoacidosis, hyperosmolar hyperglycemic state) within 1 month prior to enrollment;
4	Coexisting gastrointestinal diseases (including persistent vomiting, confirmed/suspected gastrointestinal obstruction, short bowel syndrome, inflammatory bowel disease, active major gastrointestinal bleeding, gastrointestinal perforation, high-output intestinal fistula, severe diarrhea, significant fibrotic intestinal stenosis, fulminant colitis, or toxic megacolon), history of gastrointestinal surgery (such as gastrectomy, pelvic anastomosis, colostomy, or other digestive system procedures), malignancy, or sepsis;
5	Coexisting severe cardiovascular or cerebrovascular diseases, hepatic or renal disorders, hematopoietic system diseases, severe immunosuppression, or congenital/acquired immunodeficiency diseases;
6	Recent administration of high-risk immunosuppressive or cytotoxic medications: e.g., rituximab, doxorubicin, or medium-to-high dose corticosteroids (prednisone ≥20 mg/day) for more than 4 weeks;
7	Use of closed-loop systems or sensor-augmented pumps within the past 3 months;
8	Concurrent participation in another randomized clinical trial;
9	Pregnancy, lactation, or planned pregnancy during the study period in female participants;
10	Inability to adhere to the recommended intervention and follow-up schedule for any reason.

**Abbreviations:** T1DM, type 1 diabetes mellitus; HbA1c, glycated hemoglobin; MDI, multiple daily insulin injections; TDD, total daily insulin dose.

### 2.4. Procedures

#### 2.4.1. Screening (Day −17).

Screening (Day −17, Outpatient): Informed consent will be obtained, and eligibility will be assessed against all inclusion and exclusion criteria. Eligible participants will be provided with a CGM device.

#### 2.4.2. Run-in Period and Baseline Assessments (Day −17 to Day 0).

This period standardizes participants’ status prior to intervention and consists of two phases:

*Outpatient Phase* (Day −17 to −3): Participants will wear CGM and undergo insulin dose optimization. During this period, participants are instructed to follow a standardized diet according to the Chinese Diabetes Dietary Guidelines (2017). Dietary adherence is monitored through food records and regular follow-up communications via WeChat or outpatient visits. CGM data from this phase serves as the baseline for glycemic metrics, including TIR, TBR3.9, glucose management indicator (GMI), coefficient of variation (CV), and other metrics.

*Inpatient Phase* (Day −3–0): Participants are hospitalized for baseline assessments and bowel preparation:

(1) Baseline Assessments (Completed by Day −3) include:
  a. Demographics and clinical parameters: age, sex, T1DM duration, anthropometrics (height, weight, waist-to-hip ratio [WHR]), vital signs (blood pressure, pulse), and comprehensive medical/medication history;
  b. Glycemic and lipid profiles: fasting plasma glucose, 2-hour postprandial glucose via Mixed-Meal Tolerance Test [MMT], HbA1c, total cholesterol (TC), triglycerides (TG), low-density lipoprotein cholesterol [LDL-c], and high-density lipoprotein cholesterol [HDL-c];
  c. β-cell function and insulin sensitivity (fasting/stimulated 2-hour C-peptide during Mixed-Meal Tolerance Test [MMT 2h CP], estimated glucose disposal rate [eGDR]);
  d. Diabetic autoantibodies: Glutamic Acid Decarboxylase Autoantibodies [GADA], Insulinoma-associated antigen-2 autoantibodies [IA‑2A], Islet cell autoantibodies [ICA];
  e. Inflammatory markers: white blood cell count [WBC], High-Sensitivity C-reactive protein [hs-CRP], erythrocyte sedimentation rate [ESR], procalcitonin [PCT], and cytokines [TNF-α, IL-1β, IL-8, IFN-γ];
  f. Intestinal Barrier-Related Antibodies: Anti-Lipopolysaccharide [Anti-LPS], Anti-Occludin;
  g. Immune marker: Treg/Th17 ratio;
  h. Gut microbiome and serum metabolomics;
  i. Patient-reported outcomes (PROs): Simplified Chinese version of the EQ-5D-5L, Diabetes Distress Scale [DDS], Chinese version of the Hypoglycemia Fear Survey II-Worry Scale [CHFSII-WS];
  j. Laboratory safety indicators: liver and renal function.

Detailed protocols for all laboratory measurements, including sample processing, storage conditions, and analytical methods, are summarized in S2 File.

(2) Bowel Preparation: from Day −3, participants receive oral rifaximin (0.2 g per dose, four times daily), with bowel preparation using a polyethylene glycol solution performed on Day 0.

#### 2.4.3. Randomization and blinding.

Following the completion of the run-in period, eligible participants will be randomized in a 1:1 ratio to either the intervention or the control group. Randomized allocation sequences will be generated using a permuted block design in R software. Sequentially numbered, opaque, sealed envelopes will be prepared and stored by a non-investigational team member. To minimize selection bias, the randomization codes are assigned by an independent researcher. Once an eligible participant is enrolled, the principal investigator notifies the independent researcher to open sequentially the sealed envelope for allocation. yFMT and placebo capsules are identical in appearance, shape, odor, and specifications.

The investigational capsules will be assigned predefined random codes that were dispensed without selection. Randomization number is not allowed to be reassigned following participant discontinuation. The randomization code will be securely maintained by an independent statistician throughout the study. The code will remain inaccessible to investigators, participants, and endpoint assessors, except for authorized access by the independent Data Monitoring Committee (DMC) during the pre-specified interim analysis. Full unblinding will occur only after database lock and completion of the final analysis.

#### 2.4.4. Study intervention.

**Fig 3:**
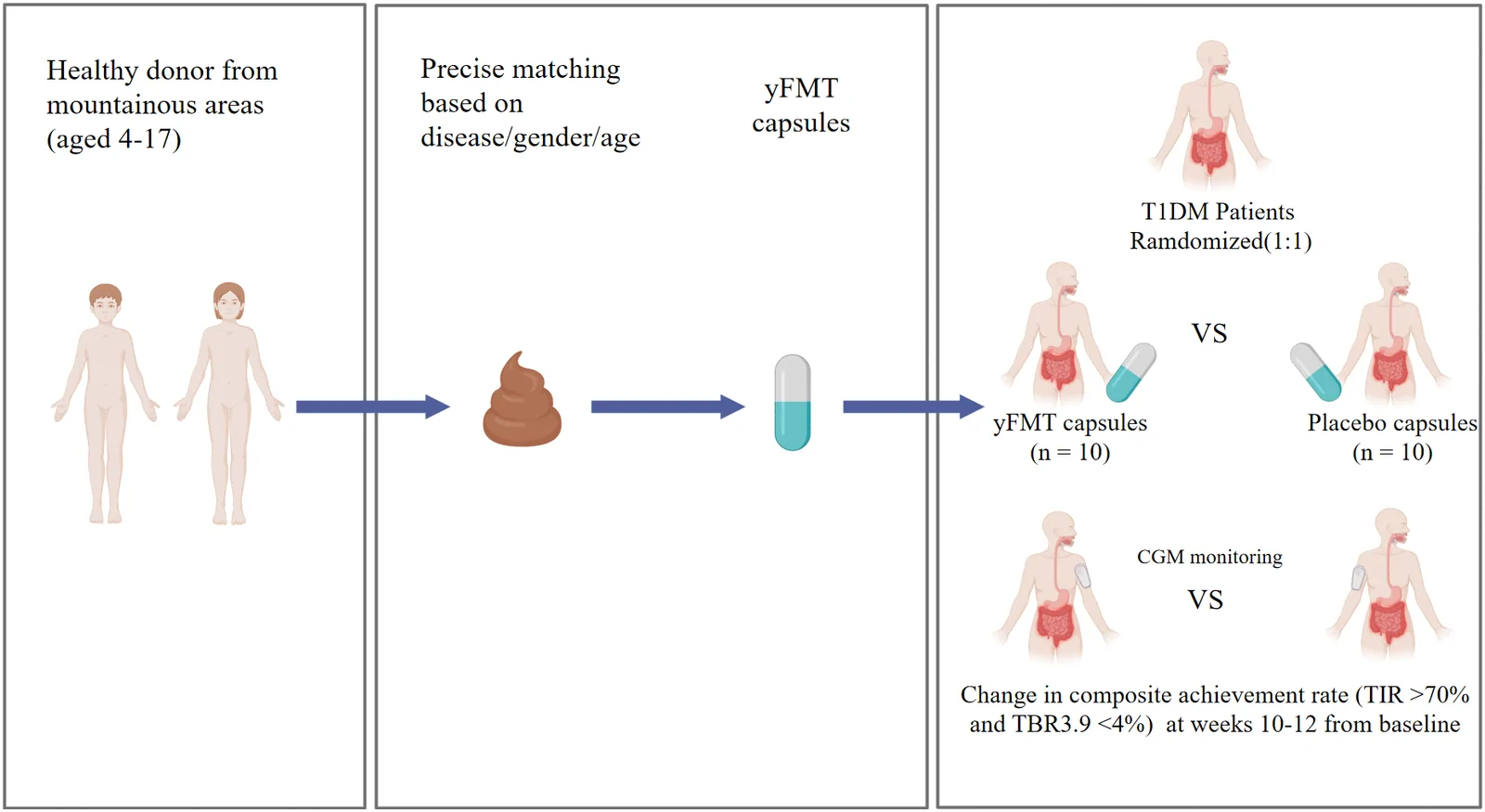
Source and allocation of gut microbiota capsules. Abbreviations: yFMT, youth-derived fecal microbiota transplantation; T1DM, Type 1 diabetes mellitus; TIR, Time in Range; TBR3.9, Time Below Range <3.9mmol/L;.

(1) Regimen: The yFMT capsules are manufactured by Meiyitian Biomedical Co., Ltd. (Wuhan, Hubei, China) and prepared from rigorously screened healthy young donors (aged 4–17 years), selected for the relatively high temporal stability of the gut microbiota during this developmental window [19,20]. Donor-recipient matching follows a three-dimensional framework based on disease state, sex, and age (Fig 3). All donor screening, infectious disease testing, and microbiological safety procedures comply with the Chinese expert consensus on FMT donor management [21], with dual informed consent obtained from both donors (when age-appropriate) and their legal guardians. Eligible participants will be randomized to receive either yFMT capsules or identical capsules (skimmed milk and starch). The administration protocol is as follows: 6 capsules are to be taken orally twice daily (a total of 12 capsules/day) under fasting conditions (at least 2 hours before or after meals) for six consecutive days, delivering a total cumulative dose of approximately 2.0 × 10^^^ [13] viable bacteria per course [22]. All capsules must be swallowed whole without chewing to maintain bacterial viability and blinding. All participants maintain their usual insulin therapy throughout the study period and are allowed to adjust their insulin dose based on CGM data.
(2) Storage and Handling: Capsules are stored at −80°C and must be administered within 20 minutes of thawing.
(3) Safety Monitoring: During the 6-day intervention period, participants will be monitored daily for:
  a. Gastrointestinal tolerance: specifically assessing bloating, abdominal pain, changes in bowel habits (constipation or diarrhea), nausea, or vomiting.
  b. Systemic symptoms: fever, rash, or any new allergic-like reactions.
  c. Glucose metabolism: close review of CGM data for significant or unexplained glycemic fluctuations, particularly hypoglycemia.

Any adverse events (AEs) will be recorded, and their relationship to the study intervention will be assessed using a predefined causality algorithm specific to microbiota-based therapies.

#### 2.4.5. Follow-up visits.

(1) Interim Visit (Week 4 ± 3 days, outpatient): Includes clinical evaluation, laboratory tests (metabolic, β-cell function, immune markers), CGM data collection (Weeks 2–4), PROs, and AEs/Serious adverse events (SAEs) documentation.
(2) Final Visit (Week 12 ± 3 days, outpatient): A comprehensive reassessment, including all parameters from the interim visit plus final gut microbiome and safety laboratory samples. CGM data from Weeks 10–12 are collected. The study concludes with database lock and unblinding.

A detailed schedule is provided in Table 2.

**Table 2:** Study visit schedule.

Process	Screening period	Run-in period 1	Baseline period	Run-in period 2	Intervention period	Mid-term follow-up	Final follow-up
	Day −17	Day −17 to Day −3	Day −3	Day −3 to Day 0	Day 0 to Day 6	Week 4 ± 3 days	Week 12 ± 3 days
Obtain informed consent	**√**						
Basic characteristics	**√**						
Lifestyle survey			**√**				
Record anthropometric measurements			**√**			**√**	**√**
Average total daily insulin dose (Past 1–2 Weeks)			**√**			**√**	**√**
Glucose + C-peptide (Fasting and MMT 2h)			**√**			**√**	**√**
HbA1c	**√**		**√**			**√**	**√**
Islet autoantibody titer			**√**			**√**	**√**
Treg/Th17 ratio			**√**			**√**	**√**
Lipids profile			**√**			**√**	**√**
Liver function			**√**				**√**
Renal function			**√**				**√**
Thyroid function			**√**				**√**
Infection markers and inflammatory cytokines			**√**			**√**	**√**
Intestinal Barrier-Related Antibodies			**√**			**√**	**√**
Safety assessments			**√**		**√**	**√**	**√**
AEs and SAEs			**√**			**√**	**√**
Gut microbiome			**√**			**√**	**√**
Serum metabolomics			**√**				**√**
Bowel cleansing				**√**			
Oral yFMT capsules					**√**		
Standard insulin therapy		**√**	**√**	**√**	**√**	**√**	**√**
CGM monitoring		**√**			**√**	**√**	**√**
Questionnaires			**√**			**√**	**√**
Feasibility endpoints							**√**
Provision of yFMT to the placebo group after unblinding							**√**

**Note:** Basic characteristics: age, sex, ethnicity, height, weight, waist circumference, hip circumference, duration of T1DM, past medical history, and medication history; Lifestyle survey: smoking, alcohol consumption, diet, and physical activity; Record anthropometric measurements: height, weight, blood pressure, pulse, waist circumference, hip circumference, BMI, and WHR; Infection markers: WBC, ESR, hs-CRP, PCT; cytokines: TNF-α, IL-1β, IL-8, IFN-γ; Intestinal Barrier-Related Antibodies: Anti-LPS, Anti-Occludin Antibodies; Safety assessments: body temperature, pulse, respiration, and blood pressure; Adverse events and serious adverse events: including abnormalities in liver and kidney function tests, as well as symptoms such as nausea, abdominal pain, diarrhea, abdominal distension, fatigue, constipation, loss of appetite, and allergic reactions. SAEs refer to medical events occurring during the clinical trial that result in hospitalization or prolonged hospitalization, disability, impaired work capacity, life-threatening conditions, death, or congenital malformations; Completion of relevant questionnaires: Simplified Chinese version (Mainland China) of the EQ-5D-5L scale, Chinese version of the Hypoglycemia Fear Survey-Worry Subscale (HFS-WS), and the Diabetes Distress Scale (DDS). Feasibility endpoints: Recruitment rate, Retention rate, Intervention adherence, Acceptability.

**Abbreviations:** MMT 2h, Mixed-Meal Tolerance Test, 2 hours; HbA1c, glycated hemoglobin; Treg, Regulatory T cell; Th17, T helper 17 cells; yFMT, youth-derived Fecal Microbiota Transplantation; CGM, Continuous Glucose Monitoring; T1DM, Type 1 Diabetes Mellitus; BMI, Body Mass Index; WHR, Waist-to-Hip Ratio; WBC, White Blood Cell; ESR, Erythrocyte Sedimentation Rate; hs-CRP, High-Sensitivity C-reactive Protein; PCT, Procalcitonin; TNF-α, Tumor Necrosis Factor-alpha; IL-1β, Interleukin-1β; IL-8, Interleukin-8; IFN-γ, Interferon gamma; Anti-LPS, Anti-Lipopolysaccharide Antibody.

#### 2.4.6. Post-study procedures.

To ensure ethical equity, all consenting participants in the placebo group will be eligible to receive a complimentary course of open-label yFMT upon study unblinding, regardless of baseline glycemic status. This extension phase utilizes a treatment regimen and dose identical to those of the yFMT group. Data from this open-label extension phase will be analyzed separately and are not included in the primary analysis. Post-treatment, participants are advised to undergo HbA1c testing and 14-day CGM monitoring at 1 and 3 months. Concurrently, they are instructed to self-report any subsequent adverse events via the 24-hour safety hotline.

#### 2.4.7. Strategies to enhance feasibility and adherence.

To ensure successful recruitment, retention, and protocol adherence, the following strategies will be implemented. Recruitment: Potential participants will be identified through the established T1DM cohort at our center, supplemented by community referrals and social-media-based patient community recommendations. Study information will be disseminated through posters, WeChat official accounts, and direct mailings to registered patients. Retention and Adherence: (1) All study-related clinical assessments and laboratory tests are provided free of charge to participants; (2) participants will receive reminder calls and WeChat messages before each scheduled visit; (3) flexible rescheduling within the ± 3-day window is permitted; and (4) participants can communicate directly with the study coordinator via WeChat for real-time queries and support. Intervention Adherence: During the 6-day inpatient intervention period, study nurses will directly observe each capsule administration. During the outpatient follow-up phase, adherence will be monitored by counting returned capsules and recorded in the case report form. These measures are designed to minimize missing data and ensure the feasibility of this pilot trial.

### 2.5. Endpoints

The primary endpoint is change in the composite achievement rate of TIR > 70% and TBR3.9 < 4% at 3 months (Weeks 10–12) post-intervention compared to baseline (days −17 to −3 during the run-in period). Secondary efficacy endpoints included glycemic metabolism, β-cell function, insulin sensitivity, immunomodulation, Intestinal Barrier-Related Antibodies, inflammatory markers, gut microbiome and metabolites, PROs, lipid profile and TDD. The 1-month (Weeks 2–4) assessment is defined as a secondary endpoint to evaluate early glycemic changes. Feasibility endpoints include: (1) recruitment rate (proportion of screened participants enrolled); (2) retention rate (proportion of participants completing the 12-week follow-up); (3) intervention adherence (percentage of prescribed capsules consumed); and (4) acceptability (participant-reported tolerability and willingness to undergo treatment). Safety endpoints comprised the incidence of AEs (such as gastrointestinal symptoms, infection-related events, immune-related reactions, and allergic responses), as well as the incidence of serious AEs (SAEs, including severe hypoglycemia, diabetic ketoacidosis, hospitalization, disability, life-threatening events, or death). Detailed information is provided in Table 3.

**Table 3:** Study endpoints.

Primary endpoint	change in the composite achievement rate (TIR > 70% and TBR3.9 < 4%) at 3 months (Weeks 10–12) post-intervention
**Secondary endpoints**	change in the composite achievement rate (TIR > 70% and TBR3.9 < 4%) at 1 month (Weeks 2–4) post-intervention
	Changes in glycemic parameters at Week 4 and Week 12 post-intervention
	Changes in pancreatic β-cell function at Week 4 and Week 12 post-intervention
	Changes in insulin sensitivity indicators at Week 4 and Week 12 post-intervention
	Changes in TDD at Week 4 and Week 12 post-intervention
	Changes in infection and inflammatory markers at Week 4 and Week 12 post-intervention
	Changes in Intestinal Barrier-Related Antibodies at Week 4 and Week 12 post-intervention
	Changes in cellular immune indicators at Week 4 and Week 12 post-intervention
	Changes in lipid profile at Week 4 and Week 12 post-intervention
	Changes in gut microbiota composition and abundance at Week 4 and Week 12 post-intervention
	Changes in serum metabolomic profiles at Week 12 post-intervention
	Changes in PROs at Week 4 and Week 12 post-intervention
**Feasibility endpoints**	Recruitment rate
	Retention rate
	Intervention adherence
	Acceptability
**Safety endpoints**	Number of adverse events and serious adverse events

**Note:** All changes in the above-mentioned indicators are relative to baseline values. Glycemic parameters: including HbA1c, FBG, MMT 2h PG and other CGM metrics [TIR, TBR3.9, TAR, GMI, GRI, CV]; Pancreatic β-cell function: Including FCP, MMT 2h CP, and the AUC of MMT 2h CP; Insulin sensitivity indicators: Including eGDR; Infection and inflammatory markers: Including WBC, PCT, ESR, hs-CRP, and inflammatory cytokines; Intestinal Barrier-Related Antibodies: Including Anti-LPS, Anti-Occludin Antibodies; Cellular immune indicators: Including peripheral blood Treg/Th17 ratio and islet-related autoantibodies [GADA, IA-2A, ICA]; Lipid profile: Including TC, TG, LDL-c, and HDL-c; Serum metabolomic profiles: Including bile acids [e.g., UDCA, TUDCA] and SCFAs; PROs: Including EQ-5D-5L, CHFSII-WS, and DDS; Recruitment rate: Proportion of screened participants enrolled; Retention rate: Proportion of participants completing the 12-week follow-up; Intervention adherence: Percentage of prescribed capsules consumed; Acceptability; Participant-reported tolerability and willingness to undergo treatment. Serious adverse events: Including severe hypoglycemia, diabetic ketoacidosis and severe allergic reactions;

**Abbreviations:** TIR, Time in Range; TBR3.9, Time Below Range <3.9 mmol/L; HbA1c, Glycated hemoglobin; FBG, Fasting Plasma Glucose; MMT 2h PG, 2-hour Postprandial Glucose during Mixed-Meal Tolerance Test; CGM, Continuous Glucose Monitoring; TAR, Time Above Range; GMI, Glucose Management Indicator; GRI, Glycemic Risk Index; CV, Coefficient of Variation; FCP, Fasting C-Peptide; MMT 2h CP, 2-hour Postprandial C-Peptide during Mixed-Meal Tolerance Test; AUC, Area Under the Curve; eGDR, Estimated Glucose Disposal Rate; TDD, Total daily insulin dose; WBC, White Blood Cell count; PCT, Procalcitonin; ESR, Erythrocyte Sedimentation Rate; hs-CRP, High-Sensitivity C-Reactive Protein; Anti-LPS, Anti-Lipopolysaccharide; Treg, Regulatory T cells; Th17, T helper 17 cells; GADA, Glutamic Acid Decarboxylase Antibody; IA-2A, Insulinoma-Associated Antigen-2 Antibody; ICA, Islet cell autoantibodies; TC, Total Cholesterol; TG, Triglycerides; LDL-c, Low-Density Lipoprotein Cholesterol; HDL-c, High-Density Lipoprotein Cholesterol; UDCA, Ursodeoxycholic Acid; TUDCA, Tauroursodeoxycholic Acid; SCFAs, Short-Chain Fatty Acids; PROs, patient-reported outcomes; EQ-5D-5L, EuroQol Five-Dimension Five-Level Questionnaire; CHFSII-WS, Chinese version of the Hypoglycemia Fear Survey II-Worry Scale; DDS, Diabetes Distress Scale;

### 2.6. Risk and AEs

An AE is defined as any unfavorable medical occurrence in an individual after signing the informed consent form and entering the study until the last follow-up, regardless of whether it had a causal relationship with yFMT capsules. The anticipated risks associated with the intervention include gastrointestinal symptoms (e.g., nausea, abdominal pain, diarrhea, bloating, constipation), fatigue, decreased appetite, and potential metabolic or immune-related complications (e.g., diabetic ketoacidosis, severe hypoglycemia, severe allergic reactions).

In the event of any AEs, the investigators will provide prompt management and may discontinue the study intervention based on the clinical judgment of the investigator. All AEs, regardless of severity, will be recorded in the case report form and followed until satisfactory resolution. Only SAEs are required to be reported to the Institutional Review Board as mandated by the protocol and regulatory guidelines.

### 2.7. Withdrawal standard

DMC will periodically convene to make clinical judgments on who should withdraw from the study. The following withdrawal rules will be abided by (1) SAEs; (2) failure to complete the yFMT intervention per protocol (e.g., missing ≥50% of the doses); (3) pregnancy or planned pregnancy; and (4) development of a newly diagnosed condition that met exclusion criteria (e.g., inflammatory bowel disease, malignancy) or any other situation in which the investigator determined that study discontinuation was warranted. It should be noted that participants maintain the right to terminate the study at any time without impact on future care.

### 2.8. Data management

All pseudonymized research data, including demographics, laboratory data, questionnaire responses, and AE records, will be recorded in the case report form and securely maintained at Zhongnan Hospital of Wuhan University under strict confidentiality and data security protocols.

To ensure data integrity and accessibility, the following measures will be implemented: (1) all investigators will receive standardized training on data management procedures prior to trial initiation; (2) responsible investigators will perform ongoing monitoring and periodic quality checks to assess data integrity throughout the study; (3) the principal investigator will oversee the data lifecycle and audit procedures.

### 2.9. Sample size and pilot trial rationale

This is a pilot study. The primary objectives are to assess the feasibility (recruitment, adherence) and safety of the oral yFMT, to evaluate the preliminary effciency, and to collect preliminary effect-size estimates for the planning of a subsequent pivotal trial.

As no prior data exist on achieving the composite glycemic target (TIR > 70% with TBR3.9 < 4%) following yFMT in adults with T1DM, a traditional sample size calculation based on an anticipated effect size was not feasible. The sample size for this trial was therefore determined in accordance with methodological best practices for pilot trial design. We referenced the guidance proposed by Wang et al., which indicates that a pilot study for a future definitive trial with 90% power and a two-sided alpha of 0.05 typically requires approximately 10–25 participants per group to provide meaningful parameter estimates for small-to-moderate effect sizes [23]. Based on this, and considering the common requirement for assessing primary feasibility objectives (e.g., recruitment rate, procedural acceptance), a sample size of 10 participants per group was set.

A pre-planned interim analysis will be conducted after the first 10 participants (50% of the total planned sample) have completed the 12-week follow-up. During this interim analysis, the DMC will review unblinded data, including safety outcomes and the primary composite endpoint, to evaluate safety and obtain preliminary treatment effect estimates for future sample size planning. No formal hypothesis testing will be performed, and no sample size re-estimation is planned for the current trial, consistent with its pilot nature. Based on the DMC’s review, the trial may continue as planned, be modified, or be terminated early if unacceptable safety concerns or clear futility are observed.

### 2.10. Statistics and analysis

Efficacy analyses will be performed in the intention-to-treat population. For the primary efficacy endpoint, participants with missing primary endpoint assessments will be considered non-responders using a non-responder imputation approach in the intention-to-treat analysis. Safety analyses will be performed in the safety analysis set. Normality of continuous variables will be assessed using the Shapiro-Wilk test at each time point. Normally distributed data will be presented as mean±standard deviation and analyzed using independent samples t-test (between-group) or paired t-test (within-group). Non-normally distributed data will be presented as median (Q1, Q3) and analyzed using Mann-Whitney U test (between-group) or Wilcoxon signed-rank test (within-group). Categorical data will be expressed as count (percentages) and analyzed using Fisher’s exact test.

For the primary efficacy endpoint assessed from baseline to Week 12 post-intervention, a generalized estimating equation (GEE) model with a binary distribution, logit link function, and an exchangeable correlation structure will be employed. The model includes group, time, and their interaction as fixed effects, with baseline composite achievement status as a covariates, as appropriate. Given the pilot nature of the study (n = 20), Fisher’s exact test will additionally be performed at each time point as a sensitivity analysis to ensure robustness. For continuous secondary outcomes, linear mixed-effects models will be applied with group, time, and their interaction as fixed effects, the baseline value of the respective outcome as a covariate, and age and T1DM duration as additional covariates, with a random intercept for each participant. Multicollinearity will be assessed using the variance inflation factor (VIF), with VIF > 10 indicating significant collinearity. Given the limited sample size, a parsimonious sensitivity model including only baseline value, group, and time will also be performed to assess robustness against overfitting. Feasibility outcomes will be assessed descriptively. All feasibility endpoints will be presented as frequencies and percentages, with 95% confidence intervals where appropriate. For secondary continuous outcomes and covariates, missing data will be addressed using multiple imputation. Twenty imputed datasets will be generated using baseline outcome values, age, T1DM duration, and treatment group as predictors, and results will be pooled using Rubin’s rules. Logistic regression analysis will be used to explore factors associated with response to yFMT treatment. CGM metrics, as recommended by international expert consensus, will be calculated using Glyculator 2.0 software (Medical University of Lodz, Poland). All statistical analyses will be performed using R software (version 4.3.2) and GraphPad Prism 9.0. Statistical significance is defined as two-sided *P* < 0.05. Bonferroni correction will be applied for multiple comparisons in post hoc analyses.

## 3. Discussion

T1DM remains a major clinical challenge as standard insulin therapy fails to halt the underlying autoimmune destruction of pancrea,tic β cells. The well-established association between T1DM and gut dysbiosis [8,9], supported by preclinical studies in which microbial modification slowed disease progression [12,13], has positioned FMT as a viable therapeutic strategy. Recent pioneering human trials have further indicated that FMT may help preserve residual islet function and improve glycemic outcomes [14–16], yet several methodological constraints impede its translation.

Current limitations include the reliance on invasive, poorly tolerated nasojejunal tubes for delivery, which is a key barrier to clinical adoption, and the absence of standardized donor screening, which leads to inconsistent microbial compositions. Furthermore, outcomes are typically assessed using isolated glycemic metrics such as HbA1c, FBG, or isolated CGM metrics rather than integrated metabolic profiling.

To address these gaps, we propose a pilot study of yFMT capsules. The rationale for selecting young donors (aged 4–17 years) is grounded in the relatively high gut microbiota temporal stability characteristic of this developmental window [19,20]. Advanced cryogenic lyophilization and embedding technologies are employed to ensure microbial integrity and safety. Beyond this, we adopted a composite target of TIR > 70% and TBR3.9 < 4% for a more holistic assessment of efficacy and safety.

This pilot study incorporates predefined feasibility endpoints—including recruitment rate, retention rate, intervention adherence, and acceptability—which are critical for informing the design and conduct of a future confirmatory trial. Recruitment and retention rates will provide essential information on patient interest and the practicality of the study procedures. Adherence and acceptability data will help refine the intervention protocol and identify potential barriers to implementation in larger, multicenter settings. Together, these feasibility metrics, combined with the preliminary efficacy and safety data, will provide a comprehensive foundation for planning a subsequent adequately powered trial.

However, our study is also limited by a relatively small sample size, single-center design, and relatively short (12-week) follow-up period, which might be insufficient to assess long-term glycemic benefit and sustained impact on residual islet β cell function. Additionally, several recipient-related factors may further complicate the interpretation of outcomes. Longer T1DM duration has been associated with reduced residual β-cell function, potentially limiting the capacity for β-cell preservation [14]. Age may influence both gut microbiota composition and immune responsiveness [24]. Underlying comorbidities are also recognized as important determinants of the efficacy and safety of yFMT [25]. Although age and T1DM duration were adjusted for in the statistical analyses and individuals with relevant comorbidities were excluded per protocol, the limited sample size (n = 20) precludes robust subgroup analyses for these variables, as well as for other potential confounders. This further underscores a key limitation of the present pilot study and highlights the need for future adequately powered trials to systematically evaluate these potential effect modifiers.

## Supporting information

S1 File SPIRIT 2025 Checklist. (DOCX)

S2 File Detailed protocols for all laboratory measurements. (XLSX)

S3 File Protocol submitted for ethical approval. (Chinese).
(PDF)

S4 File Protocol submitted for ethical approval. (English Translation). Note: This document is an English translation of the protocol approved by the Ethics Committee. In case of discrepancies, the original Chinese version shall prevail.
(DOCX)
