# Supplementary material for: Efficacy, safety, and feasibility of youth-derived fecal microbiota transplantation among adults with type 1 diabetes mellitus: A protocol of Pilot Randomized Controlled Trial
Source: PLoS One. 2026 Sep 21;21(9):e0343078. doi: 10.1371/journal.pone.0343078 (PMC13592622; doi:10.1371/journal.pone.0343078)
Supplement: Supplementary file 1 [file pone.0343078.s003.pdf]

项目来源与编号：

初幼菌群移植对 1 型糖尿病成人的疗效及安全性：一项试点随机对照试验

组长单位：武汉大学中南医院

项目负责人：代喆

承担科室：内分泌科

联系电话：67813107

研究年限：2025 年 7 月至 2026 年 12 月

版本号：第 1.0 版

版本日期：2025 年 7 月 24 日

方 案 摘 要

**项目名称**	初幼菌群移植（yFMT）对1型糖尿病（T1DM）成人的疗效及安全性：一项试点随机对照试验
**研究目的**	本研究旨在探索yFMT对T1DM成人的疗效及安全性，通过口服给药方案和多维评估体系，验证yFMT在改善血糖控制、保护胰岛功能、改善胰岛素敏感性、调节免疫和肠道菌群等方面的潜在作用，为T1DM提供新的治疗选择。
**研究设计**	单中心、随机、双盲、平行、安慰剂对照研究
**病例总数**	20例
**病例选择**	*入选标准*<br>①由专业内分泌科医师确诊 T1DM，且病程超过 1 年以上；<br>②年龄 18-60 岁；<br>③糖化血红蛋白（HbA1c）7.0-14.0%或葡萄糖在目标范围内时间（TIR）<70%；<br>④使用每日多次注射或胰岛素泵治疗≥3 个月<br>⑤入院前每日胰岛素总剂量≥0.3IU/kg 体重/天，基础率≥0.05IU/小时<br>⑥育龄期女性愿意使用适当的避孕措施<br>⑦签署知情同意书
	排除标准<br>①使用任何除胰岛素外的降糖药物治疗；<br>②入组前3个月连续使用抗菌药物、益生菌制剂、肠道微生态调节及对肠道菌群有潜在影响的其他药物 ＞3天；<br>③入组前1个月出现糖尿病急性并发症（糖尿病酮症酸中毒、高渗性高血糖状态）；<br>④合并消化道疾病（持续呕吐或确诊/疑诊胃肠道梗阻、短肠综合征、

	炎症性肠病、消化道活动性大出血、消化道穿孔、高流量肠瘘、严重腹泻、显著纤维性肠腔狭窄、暴发性结肠炎或中毒性巨结肠者等）或消化道手术史（曾接受胃切除术、盆底吻合、结肠造口术及其他消化系统手术等）或肿瘤患者或脓毒症；<br>⑤合并严重心脑血管疾病，或肝、肾、造血系统疾病，或严重免疫抑制者、先天或获得性免疫缺陷病；<br>⑥近期接受高风险免疫抑制或细胞毒性药物治疗者：例如利妥昔单抗、阿霉素或中高剂量类固醇激素（20mg/d 泼尼松或更高剂量）持续应用 4 周以上。<br>⑦近3个月内使用闭环泵或传感器增强泵；<br>⑧同时参与另一项随机临床试验；<br>⑨妊娠或哺乳，或研究期间计划妊娠的女性；<br>⑩因各种原因无法遵循推荐的干预及随访计划。
**治疗方案**	肠道菌群提取物胶囊+胰岛素标准疗法 vs. 安慰剂胶囊+胰岛素标准疗法
**疗效评定**	*有效性评价指标（主要疗效指标和次要疗效指标）*<br>（1）主要指标：TIR>70%+血糖低于目前范围内时间（TBR）<4%的达标率（CGM 指标）<br>（2）次要指标<br>①血糖代谢<br>HbA1c：相较于基线，干预 4 周及 12 周后 HbA1c 的绝对变化值（%）。<br>空腹及餐后 2 小时血糖：相较于基线，干预 4 周及 12 周后空腹及餐后 2 小时血糖的绝对变化值（mmol/L）。<br>CGM 核心指标：相较于基线，干预 4 周及 12 周后其他 CGM 核心指标水平的绝对变化值（%）。<br>②胰岛β细胞功能评估<br>空腹 C 肽：相较于基线，干预 4 周及 12 周后空腹 C 肽水平的绝对变化

	值（ng/mL）；<br>MMT2 小时 C 肽：相较于基线，干预 4 周及 12 周后 MMT2 小时 C 肽水平的绝对变化值（ng/mL）；<br>MMT2 小时 C 肽曲线下面积：相较于基线，干预 4 周及 12 周后 MMT2 小时 C 肽曲线下面积；<br>③胰岛素敏感性：相较于基线，eGDR（基于临床指标计算，包括腰臀比、舒张压、HbA1c）在干预 4 周及 12 周后的变化。<br>④细胞免疫调节效应<br>Treg 与 Th17 比例：相较于基线，干预 4 周及 12 周后外周血中 CD4+CD25+FoxP3+（Treg）和 CD4+IL-17A+（Th17）细胞比例的变化。<br>胰岛自身抗体滴度：相较于基线，干预 4 周及 12 周后 GADA、IAA、ICA 抗体滴度及滴度下降≥50%的受试者比例的变化。<br>⑤感染指标：相较于基线，在干预 4 周及 12 周后，WBC、CRP、ESR、PCT 及炎性因子（TNF-α、IL-1β、IL-8、IFN-γ、脂多糖抗体、阻塞蛋白抗体）水平的变化。<br>⑥肠道微生态重塑<br>关键菌属丰度变化：相较于基线，干预 12 周后粪便中阿克曼菌（Akkermansia）、罗斯氏菌（Roseburia）等有益菌丰度的变化。<br>胆汁酸代谢谱：相较于基线，干预 12 周后血液及粪便中次级胆汁酸（LCA、isoLCA）及 SCFAs 等代谢物的变化。<br>微生物来源 cfRNA 的表观转录组学特征图谱变化：相较于基线，干预 12 周后血液样本 cfRNA 的甲基化位点及模式的变化。并通过统计建模，识别对与 yFMT 治疗反应相关的 cfRNA 甲基化生物标志物。<br>⑦患者报告结局<br>低血糖恐惧：相较于基线，干预 4 周及 12 周后 HFS-WS 评分的变化。<br>糖尿病相关痛苦：相较于基线，干预 4 周及 12 周后 DDS 评分的变化。<br>生活质量：相较于基线，干预 4 周及 12 周后 EQ-5D-5L 量表评分的变

	化。<br>⑧血脂代谢：相较于基线，干预 4 周及 12 周后 TC、TG、LDL-c 及 HDL-c 水平的变化。<br>⑨胰岛素剂量：相较于基线，干预 4 周及 12 周后 TDD 的变化（u/d）。
	安全性评价指标<br>① 不良事件（ADEs）的发生率：根据不良事件通用术语评价标准（CTCAE）5.0 版记录不良事件；<br>② 严重不良事件（SAEs）的发生率：严重低血糖事件、糖尿病酮症酸中毒（DKA）以及其他 SAE（是指临床试验过程中发生需要住院治疗或延长住院时间、伤残、影响工作能力、危及生命或死亡、导致先天畸形等医学事件）。
**统计方法**	所有疗效终点均基于意向治疗人群进行分析，那些未完成治疗终期评估的患者也被纳入分析，并被归类为未改善。安全性终点则在安全性分析集中进行分析。我们使用 Glyculator 2.0 软件（Medical University of Lodz，Lodz，波兰）计算了国际专家共识建议的 CGM 关键指标。<br>计量资料经 Shapiro-Wilk 正态性检验后，符合正态分布者以均值±标准差描述，采用独立样本 t 检验（组间比较）或配对 t 检验（组内比较）；非正态分布者以中位数(Q1, Q3)描述，采用 Mann-Whitney U 检验（组间）或 Wilcoxon 符号秩检验（组内）。计数资料以例数(%)表示，采用 Fisher 精确检验。主要疗效指标（复合达标率）及分类次要指标采用广义估计方程分析组别、时间及交互效应。连续型次要指标（如 HbA1c、C 肽等变化值）采用线性混合效应模型分析，固定效应包括组别、时间、交互项及基线值，并设定受试者随机截距。安全性指标（不良事件发生率）采用 Fisher 精确检验比较。使用 Logistic 回归分析来探索 yFMT 治疗后是否应答的影响因素。主要指标缺失数据采用末次观测值结转法处理。所有分析使用 R 软件（4.3.2 版）和 GraphPad Prism 9.0 进行。所有检验均为双侧，$P<0.05$ 为差异有统计学意义，对事后多重

	比较进行 Bonferroni 校正。
**研究期限**	2025.7-2026.12（1.5年）

一、研究背景

1、研究背景及意义

1.1 我国 1 型糖尿病发病率呈增长趋势，患者血糖控制不佳

1 型糖尿病（T1DM）终生依赖胰岛素治疗，可发生在任何年龄，多于儿童青少年起病[(1)]。我国最新 T1DM 发病率调查显示，中国全年龄段 T1DM 发病率为 1.01/10 万，0-14、15-29、30 岁及以上发病率分别为 1.93、1.28、0.69/10 万[(2)]。据这一最新数据，我国每年有 13000 例新诊断 T1DM，新发病患者数居于西太区第一；与既往流行病学数据比较[(3)]（14 岁以下儿童 T1DM 发病率 0.59/10 万），我国 T1DM 增加迅速；亟待加强对该领域的关注及相关研究。

教育、营养、运动、血糖监测和胰岛素治疗是 T1DM 综合管理的五架马车[(4)]。尽管近年来新型胰岛素剂型、持续葡萄糖监测系统（CGM）、新型胰岛素泵等为 T1DM 的血糖监测和治疗提供了更好的手段。然而，国内外相关数据提示 T1DM 管理仍面临对患者自我管理能力要求高、整体人群血糖控制差、低血糖频发等多种挑战[(5-10)]。急需采取相关措施改善以上不良现况。

1.2 肠道菌群紊乱在 T1DM 发病中扮演关键角色

T1DM 的核心病理机制是自身免疫介导的胰岛β细胞破坏。传统胰岛素治疗虽能控制血糖，但无法阻断自身免疫进程或恢复β细胞功能。近年来，人们越来越意识到肠道菌群作为“第二基因组”在 T1DM 发病中的关键作用[(11)]。多项研究表明，T1DM 患者肠道菌群呈现特征性改变，包括产丁酸盐菌减少、次级胆汁酸（如 LCA、isoLCA）水平下降和致病菌增加（拟杆菌门/厚壁菌门比例降低），伴随肠道屏障功能受损，形成“菌群-肠-胰岛轴”紊乱[(12,13)]。

1.3 粪菌移植为治疗 T1DM 提供了新的希望

粪菌移植（FMT）是通过将健康供体的肠道菌群移植至患者肠道内，重建微生态平衡的颠覆性技术，动物研究证实通过调整肠道菌群结构可改善或延缓 T1DM [14,15]。因此 FMT 具有治疗 T1DM 的潜力，不仅可能改变胰岛素敏感性，还可能通过减弱潜在的自身免疫来改变 T1DM 的病程。其作用途径可能包括：（1）恢复产短链脂肪酸（SCFAs）菌群，改善肠屏障功能 [16]；（2）调节自身免疫反应 [17]；（3）通过肠-脑轴调控代谢；（4）抑制肠杆菌科等致病菌过度增殖 [18]。

2、国内外研究现状分析及存在问题

2.1 FMT 治疗 T1DM 的研究进展

目前FMT在T1DM患者中应用的证据还相对缺乏。一项RCT研究比较了自体 FMT 与来自健康供体的同种异体 FMT 对于延缓胰岛β细胞功能下降的疗效[19]。研究表明，同种异体 FMT 组的β细胞下降速度似乎确实比在其他几项临床试验中显示的要慢，但在接受了 12 次自体 FMT 而非来自健康供体的 FMT 后，β细胞下降的速度保留了 3 个月，并改善血糖控制；宏基因组分析发现狄氏副拟杆菌等菌群变化与疗效相关。刘煜团队研究证实，洗涤粪菌移植（WMT）能显著改善胰岛功能完全丧失的脆性糖尿病患者的血糖波动（降低 MAGE、CV），并提高治疗满意度[20]。该团队进一步研究发现 WMT 可提升 LCA、isoLCA 水平，与胰岛功能改善相关[21]。以上研究菌群移植的方式均为经鼻肠管菌液输注。 另有两项 RCT 分别比较了 FMT 胶囊和安慰剂胶囊对于 T1DM 合并严重胃肠道神经病变患者[22]及合并糖尿病周围神经病变[23]的安全性及有效性，FMT 口服胶囊改善了患有肠道症状的 T1DM 患者的胃肠道症状以及生活质量，显着改善了患者周围神经病变的严重程度。以上研究均报告了粪菌移植的良好安全性，主要不良事件为轻度胃肠道反应[19-23]。最常见的不良事件是恶心、腹痛、腹泻、腹胀、疲劳、便秘和食欲下降。

2.2 现存问题

尽管现有研究已初步证实 FMT 在 T1DM 治疗中的潜在价值，但当前研究仍存在以下

关键科学问题亟待解决：

（1）研究靶点局限：

- 现有研究多聚焦于 FMT 对残余胰岛β细胞功能的保护作用，但研究结论存在异质性；
- 缺乏对 FMT 调节自身免疫（如胰岛相关抗体滴度变化）及改善胰岛素敏感性的系统评估；

（2）疗效评价体系不完善：

- 血糖监测指标单一：多数研究仅采用糖化血红蛋白（HbA1c）、空腹血糖等传统指标或孤立的 CGM 参数（如葡萄糖在目标范围内时间[TIR]或葡萄糖低于目标范围时间[TBR]）；
- 缺乏“双达标”综合评估（TIR>70%联合 TBR<4%），难以全面反映血糖控制的质与量；

（3）技术标准化不足：

- 供体筛选缺乏金标准：未系统考虑代谢特征、菌群组成、遗传背景等多维参数；
- 移植技术差异大：菌群制备工艺、移植途径等关键参数缺乏统一标准；

（4）临床应用瓶颈：

- 现有的研究多采用鼻空肠管给药，存在技术要求高、患者耐受性差等问题；
- 口服 FMT 胶囊便利性更高（不仅可以避免侵入性操作，同时减少了让捐赠者和受试者在同一天反复去医院的繁琐），而且不良反应较少。

针对上述问题，本研究首次提出利用“三代 FMT 技术”——初幼菌群移植（youth-derived FMT, yFMT），通过口服胶囊给药方式开展一项小样本随机、双盲、平行、安慰剂对照试点研究系统评估其对成人 T1DM 的多维干预效果，包括对 T1DM 患者血糖控制（采用“双达标”评价）、胰岛功能（空腹 C 肽、MMT2 小时 C 肽及其曲线下面积）、胰岛素敏感性（eGDR）及细胞免疫状态（GADA、IAA、ICA 滴度）的影响，为 T1DM 的微生物治疗提供新范式。

yFMT 技术具有三大创新优势：①精准供体体系：年龄严格限定（4-17 岁），捕获肠道菌群多样性峰值期；建立疾病/性别/年龄三维配型系统；②质控革新：引入 16S

rRNA+宏基因组双平台筛选；实施供体-受体动态匹配算法；③工艺升级：采用低温冻干包埋技术保持菌群活性；建立严格的有害菌清除标准（<0.1%）；

二、研究目的

1.主要目的：评估 yFMT 对 T1DM 成人血糖控制的疗效及安全性。

2.次要目的：

①探究 yFMT 对残余β细胞功能的保护作用。

②探究 yFMT 对胰岛素敏感性的调节效应；

③分析 yFMT 对细胞免疫的调节：

④探讨 yFMT 对肠道菌群及代谢物的影响：

⑤探索性分析：筛选预测 yFMT 疗效的微生物标志物。

三、研究设计类型、原则与试验步骤

1.研究设计

本研究是一项小样本单中心、随机、双盲、平行、安慰剂对照试点研究，在武汉大学中南医院进行，旨在初步评估 yFMT 对成人 T1DM 患者的疗效与安全性。研究包括为期 17 天的导入期、6 天的治疗期及 3 个月的随访期。导入期内（第-17 至-3 天）可以允许优化患者胰岛素剂量，并按照《中国糖尿病饮食指南（2017）》进行饮食指导，佩戴 CGM，并收集 CGM 数据作为基线血糖数据。导入期第-3 天至 0 天需要住院口服利福昔明并进行肠道准备。导入期结束后，符合条件的受试者将按照 1:1 比例随机分配至干预组及对照组，干预组将接受来自健康受试者新鲜粪便的肠道菌群提取物胶囊，6 粒日两次，连续干预 6 天；对照组接受安慰剂胶囊治疗（用法用量同肠道菌群提取物胶囊）。两组均继续接受常规胰岛素治疗方案（继续既往使用的胰岛素方案）治疗。在干预开始后第 4 及 12 周对受试者分别进行安全性及干预效果评估。在主要终点评估（3 个月随访结束）后进行揭盲。揭盲后，安慰剂组的参与者将有机会接受一次免费的 yFMT 治疗，治疗方案与干预组相同，即 6 粒日两次，连续 6 天。这一措施是出于伦理考虑，确保所有参与者都能受益，但参与者需自愿选择是否接受。研究方

案已获武汉大学中南医院伦理委员会的审查和批准。在试验开始前，研究人员向受试者说明了试验的性质、目的、流程、潜在益处以及可能出现的不良风险，受试者同意后需签署知情同意书。知情同意书中将明确告知受试者，如果他们被分配到安慰剂组，在研究结束后将有机会接受一次免费的初幼菌移植。

2.随机化

在完成导入期后，所有符合资格的受试者按照 1：1 的比例随机分配至干预组或对照组。分配过程采用计算机生成的排列区组随机化编码（该编码由统计学专家使用 R 软件预先准备，并由独立的研究者根据随机序列表顺序组装成密封的不透明信封）。为减少选择偏倚，随机分组的编码由独立的研究者进行分配。一旦合格受试者入组，主要研究者需通知独立研究者按顺序打开密封信封以执行分配。

3.设盲水平

本研究采用双盲，单模拟设计，以肠道菌群提取物安慰剂胶囊为对照，其外观、形状、气味、规格要求与肠道菌群提取物胶囊一致。对试验药物行随机编码按照临床试验随机化方案，研究者应严格依照药品编号发放药品，不得选择药品，编号在整个试验过程当中保持稳定不变。对于因任何原因退出/撤出试验的已随机分组的受试者，无论是否已给予试验药物，其随机号不能分配给其他患者再次使用。为确保研究终点评估的客观性，将对评估研究终点的研究人员及研究受试者设盲，电子化盲底保存在非盲统计师处，研究结束前不得揭盲。

4.样本量计算

由于 yFMT 在 T1DM 领域的复合血糖达标率（TIR>70%+TBR<4%）尚无文献报道，因此无法计算样本量。借鉴既往试点研究的样本量（n=10-20），本研究拟纳入 20 例符合标准的成人 T1DM 患者，即干预组与对照组各 10 例。

5.研究步骤

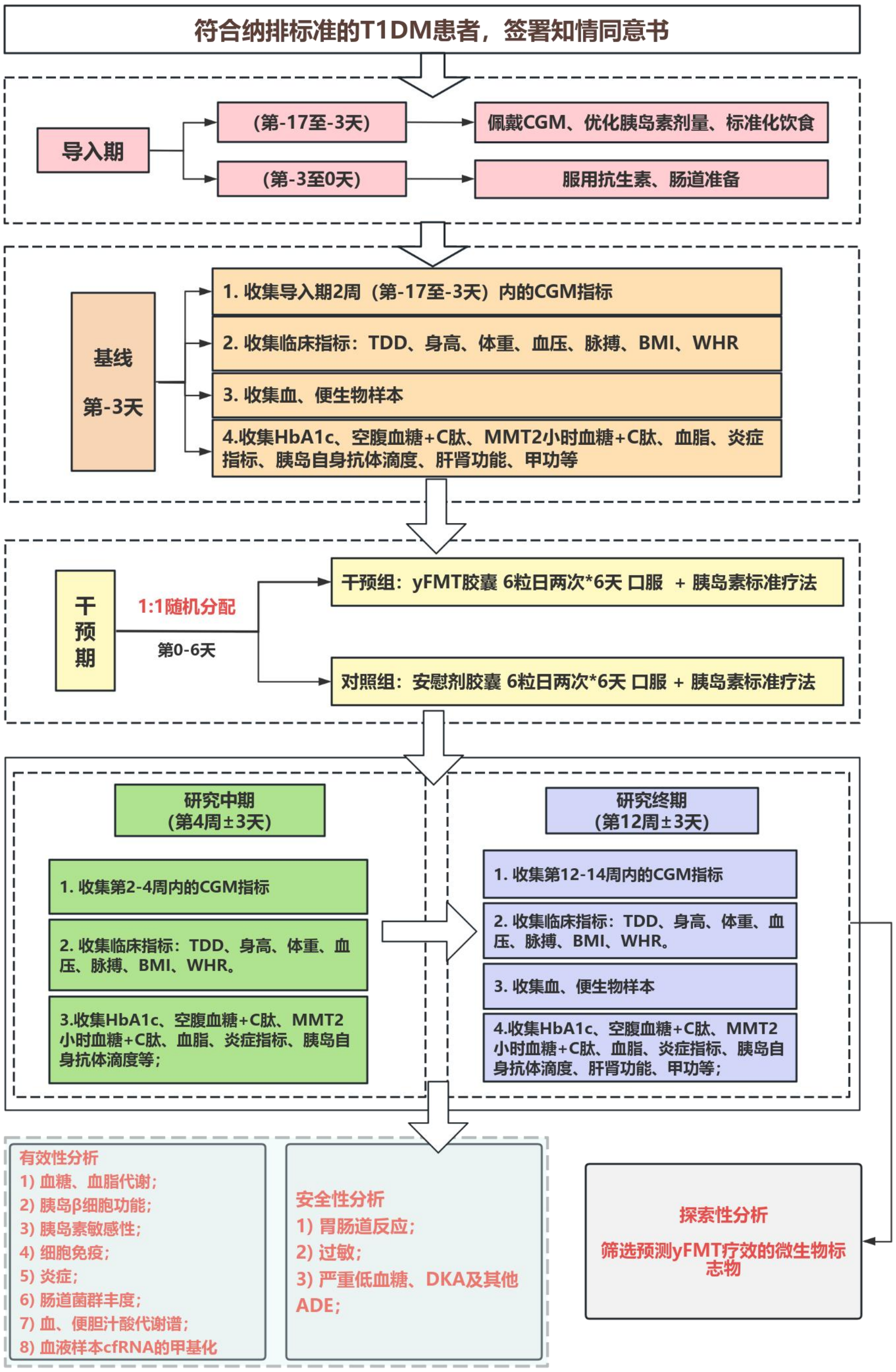

四、病例选择

1. 入选标准

①由专业内分泌科医师确诊 T1DM，且病程超过 1 年以上；

②年龄 18-60 岁；

③HbA1c7.0-14.0%或 TIR<70%；

④使用每日多次注射或胰岛素泵治疗≥3 个月

⑤入院前每日胰岛素总剂量≥0.3IU/kg 体重/天，基础率≥0.05IU/小时

⑥育龄期女性愿意使用适当的避孕措施

⑦签署知情同意书

2. 排除标准

①使用任何除胰岛素外的降糖药物治疗；

②入组前 3 个月连续使用抗菌药物、益生菌制剂、肠道微生态调节及对肠道菌群有潜在影响的其他药物> 3 天；

③入组前 1 个月出现糖尿病急性并发症（糖尿病酮症酸中毒[DKA]、高渗性高血糖状态）；

④合并消化道疾病（持续呕吐或确诊/疑诊胃肠道梗阻、短肠综合征、炎症性肠病、消化道活动性大出血、消化道穿孔、高流量肠瘘、严重腹泻、显著纤维性肠腔狭窄、暴发性结肠炎或中毒性巨结肠者等）或消化道手术史（曾接受胃切除术、盆底吻合、结肠造口术及其他消化系统手术等）或肿瘤患者或脓毒症；

⑤合并严重心脑血管疾病，或肝、肾、造血系统疾病，或严重免疫抑制者、先天或获得性免疫缺陷病；

⑥近期接受高风险免疫抑制或细胞毒性药物治疗者：例如利妥昔单抗、阿霉素或中高剂量类固醇激素（泼尼松≥20mg/d）持续应用 4 周以上。

⑦近 3 个月内使用闭环泵或传感器增强泵；

⑧同时参与另一项随机临床试验；

⑨妊娠或哺乳，或研究期间计划妊娠的女性；

⑩因各种原因无法遵循推荐的干预及随访计划。

3. 剔除标准

①违反入组标准（误纳入）

a. 事后发现不符合主要入组条件（如实际病程<1 年、合并使用非胰岛素降糖药

等）；

b. 基线数据严重缺失（如未完成 CGM 监测、未采集血样及粪便样本等）；

②严重依从性问题

a. 未完成 yFMT 干预（服用胶囊<50%剂量）；

b. 导入期或干预期间擅自改变治疗方案（如自行使用抗生素）；

c. 饮食严重偏离指南建议（如长期高脂/高糖饮食，影响菌群定植）；

③数据不可靠或缺失

a. 关键指标（如 TIR、C 肽）数据缺失≥50%（如拒绝复查、设备故障）；

b. 发现数据造假或严重记录错误（如人为修改血糖日志）；

④其他不可控因素

a. 失访（无法完成≥2 次访视）；

b. 因非研究相关疾病退出（如意外骨折、非糖尿病急性疾病住院）。

4. 终止研究标准

①安全相关：

a. 发生严重不良事件（SAE，如 DKA、严重低血糖、严重过敏反应）；

b. 持续 3 级及以上胃肠道不良反应（如严重腹泻、呕吐、腹痛）且对症治疗无效；

c. 新发自身免疫病或原有免疫疾病恶化（如甲状腺炎、乳糜泻等）；

②依从性差或主动退出：

a. 未按方案完成 yFMT 干预（如漏服≥50%剂量）；

b. 受试者要求退出（如因个人原因无法继续随访）；

③其他医学原因

a. 妊娠或计划妊娠（yFMT 对胎儿影响未知）;

b. 新发需排除的疾病（如炎症性肠病、肿瘤等）;

c. 其他医生认为需要退出的情况。

五、研究方法与技术路线

1.研究用药名称和规格

（1）试验组干预药物：肠道菌群提取物胶囊

规格：每粒含活菌个数≥4×10^{13}

来源：经严格筛选的健康供体的人源活性肠菌

生产单位：美益添生物医药（武汉）有限公司

有效期：-80℃保存：6 个月；室温解冻后 20 分钟后吞服

保存条件：冷冻（-80±2℃）保存，保质期 6 个月；冷冻（-20±2℃）保存，保质期 4 周。

（2）对照组干预药物：肠道菌群提取物安慰剂胶囊

成分：脱脂牛奶和淀粉及其他微量的保护剂

生产单位：美益添生物医药（武汉）有限公司

有效期：-80℃保存：6 个月；室温解冻后 20 分钟后吞服

保存条件：冷冻（-80±2℃）保存，保质期 6 个月；冷冻（-20±2℃）保存，保质期 4 周。

安慰剂外观、大小、颜色、剂型、重量、味道和气味会尽可能地与肠道菌群提取物胶囊相同，通常由没有药效和毒副作用的物质制成。需要说明的是，一旦本研究中发现受试者出现相关不良反应，研究团队将提供及时的治疗和密切的随访，确保受试者安全。

2.治疗方案

（1）肠道菌群提取物胶囊或肠道菌群提取物安慰剂胶囊

给药途径：口服

给药方法：整粒吞服，避免咬破；服药前后空腹 2 小时

单次剂量：一次 6 粒（1 板）

给药频次：每日 2 次（早晚各 1 次，建议固定时间）

疗程：连续服用 6 天（总剂量：72 粒）；前三天吞服白色胶囊，后三天吞服白绿拼色

胶囊（胶囊编码尾号带 M）。

用药时机：早晚空腹 2 小时（减少胃酸对菌群影响）

（2）胰岛素治疗方案

所有受试者将继续既往的胰岛素治疗方案（胰岛素泵或每日多次皮下胰岛素注射），包括任何剂型（速效、长效和预混胰岛素）。根据血糖监测结果调整胰岛素剂量，所有剂量调整需记录在案。详细记录每位受试者的胰岛素使用情况，包括剂量、时间和类型。记录任何胰岛素剂量的调整，以及调整的原因和结果。

3.注意事项

服用胶囊前半流状饮食一周，且用药前服用 3 天抗生素（利福昔明）后用泻药（聚乙二醇电解质散）清肠。

储存条件：-80℃保存，使用前室温解冻 10 分钟，解冻后 20 分钟内服用。

不良反应应对：轻度腹胀/腹泻：暂观察，无需停药；严重腹痛/血便：立即停药并就医。

饮食配合：干预期间避免辛辣食物，禁食麦麸类食物；

运动：在无其他合并症及并发症时，建议进行中等强度的有氧运动：每周锻炼 3～5 次，每次至少 30 分钟，总时长 150~250 分钟；运动的形式可选择快走、动感单车等。

4.合并用药

（1）允许合并使用的药物

①对症治疗药物（需记录使用原因及频率）

-胃肠道症状：蒙脱石散（腹泻）、奥美拉唑（胃酸过多）

-过敏/炎症：氯雷他定（非镇静抗组胺药）、局部糖皮质激素（如氢化可的松乳膏）

-止痛药：对乙酰氨基酚（禁用 NSAIDs）

②慢性病基础用药（需稳定剂量≥4 周）

-降压药（ACEI/ARB 类优先）

-甲状腺激素（如左甲状腺素钠）

（2）禁止合并使用的药物

①影响肠道菌群的药物

-抗生素（如青霉素、头孢类、大环内酯类）

-泻药（如聚乙二醇、番泻叶）

②免疫调节剂

-糖皮质激素（泼尼松≥5mg/d）

-免疫抑制剂（如甲氨蝶呤、环孢素）

③其他降糖药物

-口服降糖药（如二甲双胍、SGLT-2 抑制剂等）

-GLP-1 受体激动剂

④非必要中药/保健品

-含大黄、黄连等泻下或抗菌成分的中药

-酵素、纤维素补充剂

（3）特殊情况下需暂停研究的药物

①临时使用禁药（如抗生素治疗感染）

-暂停研究干预，记录为“中断”

-感染治愈后需重新筛查菌群（粪便检测），符合标准方可继续

②紧急用药（如手术需麻醉药）：报伦理委员会备案，评估是否退出研究

六、研究访视

1.筛选期（第-17 天）：在门诊完成

(1)签署电子知情同意书

(2)记录人口学资料（年龄、性别、民族、身高、体重、腰围、臀围、T1DM 病程、既

往病史、药物治疗史）及生活方式调查（吸烟、饮酒、饮食、运动）

(3)糖代谢指标：近 3 个月内 HbA1c 数据（需介于 7-14%）或 1 个月内 CGM 数据（TIR 需＜70%），若无相关数据，可测量 HbA1c。

(4)胰岛功能：近 3 个月的空腹 C 肽数据

(5)排除标准核查：近期抗生素/益生菌使用史、合并自身免疫病等

(6)符合纳排标准的患者发放三诺动态血糖监测仪

2.导入期（-17 天至 0 天）

(1)第-17 天至-3 天：该阶段在门诊完成：

①佩戴动态血糖监测：持续佩戴 14 天

②胰岛素优化：调整剂量至稳定状态

③标准化饮食：按《中国糖尿病饮食指南》执行

(2)第-3 天至 0 天：该阶段需住院完成：

利福昔明 0.2g/次 qid 连服 3 天，第 3 天晚上开始口服聚乙二醇电解质清肠

3.基线期（第-3 天）

(1)临床评估：血压、脉搏、身高、体重、腰围、臀围，计算体重质量指数[BMI]及腰臀比[WHR]；胰岛素剂量（TDD，u/kg）以及合并用药记录。

(2)实验室检测：

①代谢：*糖代谢*：空腹血糖、MMT-2h 血糖、HbA1c；

脂代谢：血脂四项（总胆固醇[TC]、甘油三酯[TG]、低密度胆固醇脂蛋白[LDL-c]、高密度胆固醇脂蛋白[HDL-c]）

②胰岛β细胞分泌功能：空腹 C 肽、MMT-2h C 肽及其曲线下面积；

③胰岛素敏感性：预估葡萄糖处置指数（eGDR，基于临床指标如舒张压、WHR 及 HbA1c 计算）；

④感染指标：血常规、ESR、CRP、PCT 以及炎症因子（TNF-α、IL-1β、IL-8、IFN-γ、脂多糖抗体、阻塞蛋白抗体等）。

⑤细胞免疫指标：GADA/IAA/ICA 抗体滴度、外周血 Treg/Th17 细胞比例；

⑥肠道微生态：肠道菌群结构、血浆与粪便代谢物以及血液样本微生物来源 cfRNA。

⑦安全性基线：肝功能、肾功能、甲功五项。

(3)收集并分析导入期 2 周内（第-17 天至-3 天）CGM 数据；

(4)完成相关量表：简体中文版本（中国大陆）EQ-5D-5L 量表、中文版低血糖恐惧调查-忧虑量表（CHFSII-WS）、糖尿病痛苦量表（DDS）

4.干预期（第 0-6 天）：需住院完成

(1)具体措施：肠道菌群提取物胶囊或安慰剂胶囊 6 粒/次 bid 连服 6 天；

(2)每日监测：胃肠道不良反应（腹泻/腹痛/呕吐）、血糖波动（佩戴 CGM，实时预警）

5.研究中期访视（第 4 周±3 天）：可在门诊完成

(1)临床评估：血压、脉搏、身高、体重、腰围、臀围，计算 BMI 及 WHR；TDD（u/kg）以及合并用药记录；

(2)实验室检测：

①代谢：*糖代谢*：空腹血糖、MMT-2h 血糖、HbA1c；

脂代谢：TC、TG、LDL-c、HDL-c

②胰岛β细胞功能：空腹 C 肽、MMT2 小时 C 肽及其曲线下面积；

③胰岛素敏感性：eGDR；

④细胞免疫指标：GADA/IAA/ICA 抗体滴度、外周血 Treg/Th17 细胞比例；

(3)收集并分析第 2-4 周内 CGM 数据作为早期疗效评估；

(4)完成相关量表：EQ-5D-5L、HFS-WS 及 DDS 量表

(5)收集并统计 ADE 与 SAE

6.研究终期访视（第 12 周±3 天）：在门诊完成

(1)临床评估：血压、脉搏、身高、体重、腰围、臀围，计算 BMI 及 WHR；TDD（u/kg）以及合并用药记录；

(2)实验室检测：

①代谢：*糖代谢*：空腹血糖、MMT-2h 血糖、HbA1c；

脂代谢：TC、TG、LDL-c、HDL-c

②胰岛功能：空腹 C 肽、MMT2 小时 C 肽及其曲线下面积；

③胰岛素敏感性：eGDR

④感染指标：

常规：血常规、ESR、CRP、PCT

炎症因子：TNF-α、IL-1β、IL-8、IFN-γ、脂多糖抗体、阻塞蛋白抗体

⑤免疫指标：GADA/IAA/ICA 抗体滴度、外周血 Treg/Th17 细胞比例；

⑥肠道微生态：肠道菌群结构、血浆与粪便代谢物以及血液样本微生物来源 cfRNA。

⑦安全指标：肝功能、肾功能、甲功五项

(3)收集并分析第 10-12 周内 CGM 数据；

(4)完成相关量表：EQ-5D-5L、HFS-WS 及 DDS 量表

(5)收集并统计 ADE 与 SAE

(6)揭盲与后续安排

- 进行揭盲。揭盲后，安慰剂组的参与者可自愿选择是否接受一次免费的 yFMT 治疗。这一措施是出于伦理考虑，旨在确保所有参与者都有机会从潜在的治疗中受益。
- 治疗方案与干预组相同，即 6 粒日两次，连续 6 天。在完成 yFMT 治疗后不再进行额外的随访，但研究团队将提供必要的支持和指导，以确保参与者的安全和福祉。
- 参与者将被告知如何报告任何不良事件，并保证研究团队将密切监测并记录任何 ADE 与 SAE，以确保所有参与者的安全。

研究访视图

项目	筛选期	导入期 1	基线期	导入期 2	干预期	研究中期访视	研究终期访视
	第-17 天	第-17 至-3 天	第-3 天	第-3 至 0 天	第 0 至 6 天	第 4 周±3 天	第 12 周±3 天

签署知情同意书	✔						
记录基本情况[a]	✔						
生活方式调查[b]			✔				
记录身高、体重、血压、脉搏、腰围、臀围、BMI 及 WHR			✔			✔	✔
近 1-2 周平均每日胰岛素总剂量			✔			✔	✔
空腹血糖+C 肽			✔			✔	✔
MMT 2h 血糖+C 肽			✔			✔	✔
糖化血红蛋白	✔		✔			✔	✔
胰岛自身抗体滴度			✔			✔	✔
外周血 Treg/Th17 细胞比例			✔			✔	✔
血脂（TC、TG、LDL-c、HDL-c）			✔			✔	✔
肝功能			✔				✔
肾功能			✔				✔
甲功五项			✔				✓
感染指标（WBC、ESR、CRP、PCT）			✔			✓	✔
炎症因子（TNF-α、IL-1β、IL-8、IFN-γ、脂多糖抗体、阻塞蛋白抗体）			✔			✔	✔
安全性记录[i]			✔		✔	✔	✔
不良事件及严重不良事件[j]			✔			✔	✔
采集生物样本[k]			✔				✔
口服利福昔明+清肠				✔			
口服 yFMT 胶囊					✔		
胰岛素标准疗法	✔	✔	✔	✔	✔	✔	✔
佩戴 CGM	✔	✔			✔	✔	✔
完成相关量表[#]			✔			✔	✔

揭盲后为安慰剂组免费提供 yFMT							✓

[a] 基本情况：年龄、性别、民族、身高、体重、腰围、臀围、T1DM 病程、既往病史、药物治疗史；

[b] 生活方式调查：吸烟、饮酒、饮食、运动；

[i] 安全性记录：体温、脉搏、呼吸、血压（常规记录）；

[j] 不良事件：包括肝肾功能结果的异常以及恶心、腹痛、腹泻、腹胀、疲劳、便秘和食欲下降以及过敏等。严重不良事件是指临床试验过程中发生需要住院治疗或延长住院时间、伤残、影响工作能力、危及生命或死亡、导致先天畸形等医学事件。由专门负责且经过培训的护士记录不良反应、持续时间（开始和结束日期）、严重程度、病程、结果、意义和针对不良反应采取的任何措施；

[k] 血液、粪便（分别留取 2 管 5ml 红头管及 EDTA 管外周血；留取 5g 大便标本）。

简体中文版本（中国大陆）EQ-5D-5L 量表、中文版低血糖恐惧调查-忧虑量表（HFS-WS）、糖尿病痛苦量表（DDS）

七、检测方法

1. CGM 血糖监测：

- 使用三诺动态血糖监测仪进行连续血糖监测。
- 收集的原始数据通过 Glyculator2.0 软件进行分析，以评估血糖控制情况。

2. 免疫细胞分析：

- 采用流式细胞术检测受试者外周血中调节性 T 细胞（CD4+CD25+FoxP3+ Treg）和辅助性 T 细胞 17（CD4+IL-17A+ Th17）的比例。
- 评估免疫细胞的变化，以探究 yFMT 对细胞免疫的调节效应。

3. 自身抗体检测：

- 通过放射免疫法检测 GADA、IAA 及 ICA 的滴度。
- 评估自身抗体的变化，以探究 yFMT 对胰岛自身免疫的影响。

4. 肠道菌群分析：

- 采集受试者粪便样本，通过 16S rRNA 基因测序技术进行肠道微生物组分析。
- 重点关注 Akkermansia、Roseburia 等特定菌属的相对丰度变化，以评估 yFMT 对肠道菌群的影响。

5. 代谢物定量分析：

➢ 采用靶向代谢组学技术，定量检测受试者血液及粪便样本中次级胆汁酸（包括石胆酸 LCA、异石胆酸 isoLCA）和短链脂肪酸（SCFAs，如乙酸、丙酸、丁酸）的浓度。

➢ 评估代谢物的变化，以探究 yFMT 对胆汁酸代谢和短链脂肪酸代谢的影响。

6. 甲基化测序：

➢ 从收集的血液样本中提取 cfRNA，使用 Low-Input Multiple Methylation Sequencing (LIME-seq)等技术，分析 cfRNA 的甲基化模式。

➢ 评估 cfRNA 甲基化模式的变化，以探索其作为 yFMT 疗效预测生物标志物的潜力。

八、疗效评定标准

1.有效性评价

（1）主要疗效指标

相较于基线，干预 4 周及 12 周后血糖控制复合达标率（TIR>70% + TBR<4%）的变化。

（2）次要指标

①血糖代谢

HbA1c：相较于基线，干预 4 周及 12 周后 HbA1c 的绝对变化值（%）。

空腹及餐后 2 小时血糖：相较于基线，干预 4 周及 12 周后空腹及餐后 2 小时血糖的绝对变化值（mmol/L）。

CGM 核心指标：相较于基线，干预 4 周及 12 周后其他 CGM 核心指标水平的绝对变化值（%）。

②胰岛β细胞功能评估

空腹 C 肽：相较于基线，干预 4 周及 12 周后空腹 C 肽水平的绝对变化值（ng/mL）；

MMT2 小时 C 肽：相较于基线，干预 4 周及 12 周后 MMT2 小时 C 肽水平的绝对变化值（ng/mL）；

MMT2 小时 C 肽曲线下面积：相较于基线，干预 4 周及 12 周后 MMT2 小时 C 肽曲线下面积；

③胰岛素敏感性：相较于基线，eGDR 在干预 4 周及 12 周后的变化。

备注：IneGDR= 4.964－0.121*HbA1c (%)-0.012*DBP (mmHg)-1.409*WHR。

④细胞免疫调节效应

Treg 与 Th17 比例：相较于基线，干预 4 周及 12 周后外周血中 CD4+CD25+FoxP3+（Treg）和 CD4+IL-17A+（Th17）细胞比例的变化。

胰岛自身抗体滴度：相较于基线，干预 4 周及 12 周后 GADA、IAA、ICA 抗体滴度及滴度下降≥50%的受试者比例的变化。

⑤感染指标：相较于基线，在干预 4 周及 12 周后，WBC、CRP、ESR、PCT 及炎性因子（TNF-α、IL-1β、IL-8、IFN-γ、脂多糖抗体、阻塞蛋白抗体）水平的变化。

⑥肠道微生态重塑

关键菌属丰度变化：相较于基线，干预 12 周后粪便中 Akkermansia、Roseburia 等有益菌丰度的变化。

胆汁酸代谢谱：相较于基线，干预 12 周后血液及粪便中次级胆汁酸及 SCFAs 等代谢物的变化。

微生物来源 cfRNA 的表观转录组学特征图谱变化：相较于基线，干预 12 周后血液样本 cfRNA 的甲基化位点及模式的变化。并通过统计建模，识别对与 yFMT 治疗反应相关的 cfRNA 甲基化生物标志物。

⑦患者报告结局

低血糖恐惧：相较于基线，干预 4 周及 12 周后 HFS-WS 评分的变化。

糖尿病相关痛苦：相较于基线，干预 4 周及 12 周后 DDS 评分的变化。

生活质量：相较于基线，干预 4 周及 12 周后 EQ-5D-5L 量表评分的变化。

⑧血脂代谢：相较于基线，干预 4 周及 12 周后 TC、TG、LDL-c 及 HDL-c 水平的变化。

⑨胰岛素剂量：相较于基线，干预 4 周及 12 周后 TDD 的变化（u/d）。

2.安全性评价：

①不良事件（ADEs）的发生率：根据不良事件通用术语评价标准（common terminology criteria for adverse events，CTCAE）5.0 版记录不良事件，包括：胃肠道反应（腹泻/

腹胀/呕吐）、感染风险（如菌群移位相关事件）、免疫相关不良事件及过敏等发生次数。

②严重不良事件（SAEs）的发生率：严重低血糖事件、糖尿病酮症酸中毒（DKA）以及其他 SAE（是指临床试验过程中发生需要住院治疗或延长住院时间、伤残、影响工作能力、危及生命或死亡、导致先天畸形等医学事件）。

九、不良事件的观察

1.不良事件的判定

（1）定义

a. 不良事件：自受试者签署知情同意书并入选研究后开始至最后一次随访期间，发生任何不利医疗事件，无论与 FMT 是否有因果关系，均判定为 AE。

b. 重要 AE：发生的任何导致针对性医疗措施（如停药，降低剂量和对症治疗）的 AE，如血液学和/或其他实验室异常。

（2）不良事件强度判定标准

在本临床研究中发生的所有临床 AE 将记录在研究病历不良事件页上，并将 AE 强度进行分级。为统一标准，事件强度分级如下：

a. 轻度：可察觉的不适感但是不影响日常活动；

b. 中度：不适感较强以致影响或减少日常活动；

c. 重度：无法工作或进行日常活动。

注意区别 AE 的严重程度和强度。重度用来描述强度，不一定是 SAE。例如头痛可能在强度上表现为重度，但不能列入 SAE，除非它符合 SAE 标准。

（3）不良事件与 FMT 的判断标准

对所有 AE 与 FMT 关系的因果分析，均按肯定有关、很可能有关、可能有关、可能无关、肯定无关五级进行判断，对前三种定为药物的不良反应。因果分析的判断指标有以下五个方面：

a.与 FMT 有合理时间顺序（即在移植后特定时间窗内发生）

b.该事件类型是 FMT 后已知或理论上可能发生的反应（符合文献）。

c. 事件的严重程度或持续时间与移植的剂量或途径相符。

d. 无法用受试者的临床状况、合并疾病或其他治疗/饮食变化来解释（其他解释）。

e. 有客观检测指标（如菌群分析、炎症指标）支持其与移植的关联

研究者应对 AE 和 FMT 以及合并药之间可能存在的关联作出评估，参照下表：

判断结果	判断指标				
	1	2	3	4	5
肯定有关	＋	＋	－	＋	＋
很可能有关	＋	＋	－	＋	?
可能有关	＋	＋	±	±	?
可能无关	＋	－	±	±	?
肯定无关	－	－	＋	－	－

说明：+：肯定，一：否定，±：难以肯定或否定，？：情况不明。

根据上表，判定以下 5 级不良事件与药物的关系：

1：肯定有关、2：很可能有关、3：可能有关、4：可能无关、5：肯定无关。

AE 发生率的计算：以 1+2+3 的病例总数作分子，全部可供 AE 评价的入选病例作为分母。

2.严重不良事件的判定

定义：SAE 是指试验药物在任何剂量下或在观察期间任何时候提示显著危害、禁忌症、副作用或者需谨慎的临床事件。AE 符合下面一条或以上标准时，归为严重 SAE：

a. 死亡；

b. 有生命危险（指出现该事件的患者在事件发生当时存在立即死亡的风险；并不包括那些如果更加严重将有可能导致患者死亡的事件）；

c. 导致住院或住院时间延长；

d. 导致持久或显著的劳动力丧失或残疾；

e. 先天性畸形缺陷。

有些还没有导致死亡、生命危险或需住院的医疗事件，经过适当的医学判断，认为其可能对病人或受试者造成危害，或需药物或外科手术治疗以避免上述情况发生时，也应视为SAE。

3.发生严重不良事件时对受试者的处理

发生AE时，研究者可根据病情决定采取的措施，一般采取的方法有：①观察，不中止FMT；②观察并终止，不用补救治疗；③中止，给予补救治疗。所有AE都应当追踪调查，详细记录处理经过及结果，直至受试者得到妥善解决或病情稳定。化验出现异常者应追踪至恢复正常或FMT前水平。追踪方式可以根据AE的轻重选择住院、门诊、家访、电话、通讯等多种形式。如果临床研究中出现大范围的非预期不良反应，药品监督管理部门、伦理委员会可以视情况决定暂停或者中止临床试验，并记录在案。

4.不良事件出现的风险预估及处置预案

本研究药物治疗存在出现恶心、腹痛、腹泻、腹胀、疲劳、便秘和食欲下降等不良反应；DKA、严重低血糖、严重过敏（呼吸困难甚至休克）等风险事件的可能性，一旦患者出现上诉任何不良反应及风险事件，医生及时予以免费的对症治疗，必要时停止干预，并给予患者一定补助。

5.不良事件的随访与记录

出现的AE，尤其是那些与研究药物相关的事件，应当随访直至它们恢复至基线状态或者趋于稳定。如果经过随访，仍无法恢复基线状态或者稳定，那么应当在研究病历中记录说明。除试验方案或者其他文件（如研究者手册）中规定不需立即报告的严重不良事件外，研究者应当书面报告所有SAE，随后应当及时提供详尽、书面的随访报告。SAE报告和随访报告应当注明受试者在临床试验中的鉴认代码，而不是受试者的真实姓名、公民身份号码和住址等身份信息。试验方案中规定的、对安全性评价重要的AE和实验室异常值，按照试验方案的要求在24小时内报告。涉及死亡事件的报告，研究者应当向伦理委员会提供尸检报告和最终医学报告。

6.实验室结果异常

研究者应当对实验室结果异常是否具有临床意义进行判断，并给出可能的解释。已经被报告的 AE 导致的实验室异常结果应同时作为 AE 记录在 AE 表中。具有临床意义的实验室检查异常满足以下一项或多项条件者，应作为独立诊断记录在研究病历的 AE 页中（不包括已被报告的 AE 导致的实验室结果异常）：

a. 伴有临床症状的；

b. 导致研究用药改变的；

c. 需要改变合并用药和（或）其他治疗措施的。

十、研究的质量控制与质量保证

1.研究过程管理

（1）成立独立的质量管控组：由不参与本试验的医师对整个试验的各个环节进行管控，保证试验的客观、真实进行。主要职责：①对试验的每个环节实施均进行监控；②监测在干预期间肠道菌群提取物胶囊的服用情况；③每周核查 CGM 佩戴、饮食记录及其他合并药物服用情况；④随机抽查 20%病例的原始记录（如胰岛素剂量调整日志）

（2）肠道菌群提取物胶囊的质量控制：①供体筛选：公司需提供供体的健康证明、菌群检测报告（如 Akkermansia 丰度）、病原体筛查（阴性）；②胶囊制备：每批次胶囊需提供活菌计数报告、菌群稳定性检测；③运输与储存：胶囊需在 -80℃或液氮条件下运输，并在研究单位指定超低温冰箱中保存，全程温度监控；建立完整的接收、储存和使用记录。

（3）样本采集与检测标准化：①血液样本：抗凝真空管采集 2 管 5ml 全血；1 小时内完成血清分离；-80℃分装冻存；②粪便样本：使用 DNA/RNA 稳定剂保存；避免菌群降解；③检测机构要求：具有 ISO 9001 质量管理体系认证证书；提供标准化操作流程证明。

2.质量控制措施

（1）参加临床试验的研究者的资格审查：必须具有临床试验的专业特长、资格和能力，经过资格审查后确定，人员要求相对固定。

（2）临床试验开始前培训：①培训内容：对于临床研究方案及其各指征具体内函有充分的理解和认识；标准化操作流程；不良反应观察与处理；②重点要求：客观记录症状；按规定时间和方法进行检查；完整填写研究病历。

（3）试验用药管理：采用药物计数法监控依从性；加强随访管理；完整记录用药情况。

（4）不良反应处理：提前告知可能的不良反应；制定应急预案；建立快速报告机制。

3.受试者依从性的管理

（1）住院期间管理：干预期全程住院观察；专人负责药物发放和服用监督；每日记录服用 yFMT 胶囊或安慰剂胶囊的情况和不良反应。

（2）随访期管理：定期随访检查；加强沟通和教育；提高随访完成率。

（3）监查员制度：由申办方任命独立监查员；定期核查研究资料；确保研究符合 GCP 要求。

十一、数据安全监查

临床研究将根据风险大小制定相应的数据安全监察计划。所有不良事件均详细记录，恰当处理并追踪直到妥善解决或病情稳定，按照规定及时向伦理委员会、主管部门和药品监督管理部门报告严重不良事件与非预期事件等；主要研究者定期对所有不良事件进行累积性回顾，必要时召开研究者会议评估研究的风险与受益；大于最小风险的研究将安排独立的数据监查员对研究数据进行监查，高风险研究将建立独立的数据安全监察委员会对累积的安全性数据以及有效性数据进行监查，以做出研究是否继续进行的建议。

十二、统计学处理

所有疗效终点均基于意向治疗人群进行分析，那些未完成治疗终期评估的患者也被纳入分析，并被归类为未改善。安全性终点则在安全性分析集中进行分析。我们使

用 Glyculator 2.0 软件（Medical University of Lodz，Lodz，波兰）计算了国际专家共识建议的 CGM 关键指标。

计量资料经 Shapiro-Wilk 正态性检验后，符合正态分布者以均值±标准差描述，采用独立样本 t 检验（组间比较）或配对 t 检验（组内比较）；非正态分布者以中位数(Q1, Q3)描述，采用 Mann-Whitney U 检验（组间）或 Wilcoxon 符号秩检验（组内）。计数资料以例数(%)表示，采用 Fisher 精确检验。主要疗效指标（复合达标率）及分类次要指标采用广义估计方程分析组别、时间及交互效应。连续型次要指标（如 HbA1c、C 肽等变化值）采用线性混合效应模型分析，固定效应包括组别、时间、交互项及基线值，随机效应为受试者。安全性指标（不良事件发生率）采用 Fisher 精确检验比较。使用 Logistic 回归分析来探索 yFMT 治疗后是否应答的影响因素。主要指标缺失数据采用末次观测值结转法处理。所有分析使用 R 软件（4.3.2 版）和 GraphPad Prism 9.0 进行。所有检验均为双侧，$P<0.05$ 为差异有统计学意义，对事后多重比较进行 Bonferroni 校正。

十三、临床研究的伦理学

临床研究将遵循世界医学大会《赫尔辛基宣言》等相关规定。在研究开始之前，由伦理委员会批准该试验方案后才实施临床研究。每一位受试者入选本研究前，研究者有责任向受试者或其代理人完整、全面地介绍本研究的目的、程序和可能的风险，并签署书面知情同意书，应让受试者知道他们有权随时退出本研究，知情同意中应作为临床研究文件保留备查。研究过程中将保护受试者的个人隐私与数据机密性。

十四、研究进度

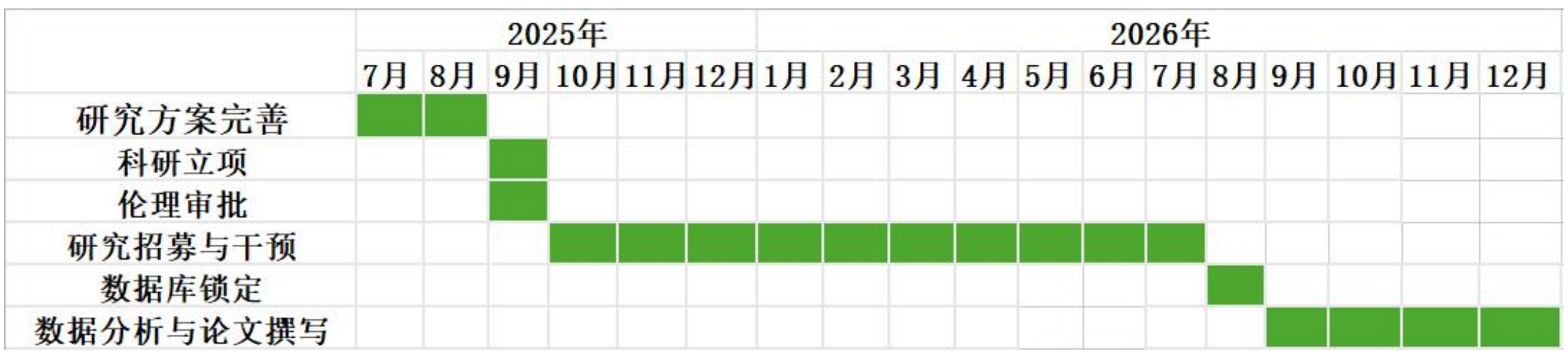

十五、参加人员

姓名	职称/专业	任务	**GCP 培训（时间）**
代喆	主任医师/内分泌科	研究设计、质量控制与监督	2024.12
雷梦云	主治医师/内分泌科	研究设计、研究执行、数据分析、论文撰写	2024.12
唐俊	副主任医师/内分泌科	研究执行、数据收集、论文撰写	2024.12
李姝婷	主管护师/内分泌科	随访与数据收集	2024.12
刘方	主管护师/内分泌科	随访与数据收集	2024.12

十六、参考文献

1. Alberti KG, Zimmet PZ. Definition, diagnosis and classification of diabetes mellitus and its complications. Part 1: diagnosis and classification of diabetes mellitus provisional report of a WHO consultation. *Diabet Med*. 1998;15(7):539-553.

2. Yang Z, Wang K, Li T, et al. Childhood diabetes in China. Enormous variation by place and ethnic group. *Diabetes Care*. 1998;21(4):525-529. doi:10.2337/diacare.21.4.525

3. Weng J, Zhou Z, Guo L, et al. Incidence of type 1 diabetes in China, 2010-13: population based study. *BMJ*. 2018;360:j5295. Published 2018 Jan 3. doi:10.1136/bmj.j5295

4. Chiang JL, Kirkman MS, Laffel LM, Peters AL; Type 1 Diabetes Sourcebook Authors. Type 1 diabetes through the life span: a position statement of the American Diabetes Association. *Diabetes Care*. 2014;37(7):2034-2054. doi:10.2337/dc14-1140

5. Beck RW, Tamborlane WV, Bergenstal RM, et al. The T1D Exchange clinic registry. *J Clin Endocrinol Metab*. 2012;97(12):4383-4389. doi:10.1210/jc.2012-1561

6. iller KM, Foster NC, Beck RW, et al. Current state of type 1 diabetes treatment in the U.S.: updated data from the T1D Exchange clinic registry. *Diabetes Care*. 2015;38(6):971-978. doi:10.2337/dc15-0078

7. Li J, Yang D, Yan J, et al. Secondary diabetic ketoacidosis and severe hypoglycaemia in patients with established type 1 diabetes mellitus in China: a multicentre registration study. *Diabetes Metab Res Rev*. 2014;30(6):497-504. doi:10.1002/dmrr.2547

8. Liu L, Yang D, Zhang Y, et al. Glycaemic control and its associated factors in Chinese adults with type 1 diabetes mellitus. *Diabetes Metab Res Rev*. 2015;31(8):803-810. doi:10.1002/dmrr.2716
9. Qin ZY, Yan JH, Yang DZ, et al. Behavioral Analysis of Chinese Adult Patients with Type 1 Diabetes on Self-monitoring of Blood Glucose. *Chin Med J (Engl)*. 2017;130(1):39-44. doi:10.4103/0366-6999.196574
10.郑雪瑛，翁建平，骆斯慧，熊珊珊，杨黛稚，邱丽玲，秦兆艺，任文倩，吕婧，姚斌，严晋华：广东省成人 1 型糖尿病患者自我血糖监测及其影响因素调查[J]．中华糖尿病杂志, 2016;8(4): 215-219.
11.Knip M, Siljander H. The role of the intestinal microbiota in type 1 diabetes mellitus. *Nat Rev Endocrinol*. 2016;12(3):154-167. doi:10.1038/nrendo.2015.218
12.de Goffau MC, Fuentes S, van den Bogert B, et al. Aberrant gut microbiota composition at the onset of type 1 diabetes in young children. *Diabetologia*. 2014;57(8):1569-1577. doi:10.1007/s00125-014-3274-0
13.Burger-van Paassen N, Vincent A, Puiman PJ, et al. The regulation of intestinal mucin MUC2 expression by short-chain fatty acids: implications for epithelial protection. *Biochem J*. 2009;420(2):211-219. Published 2009 May 13. doi:10.1042/BJ20082222
14.Calcinaro F, Dionisi S, Marinaro M, et al. Oral probiotic administration induces interleukin-10 production and prevents spontaneous autoimmune diabetes in the non-obese diabetic mouse. *Diabetologia*. 2005;48(8):1565-1575. doi:10.1007/s00125-005-1831-2
15.Mithieux G. Does Akkermansia muciniphila play a role in type 1 diabetes?. *Gut.* 2018;67(8):1373-1374. doi:10.1136/gutjnl-2017-315732
16.Allegretti JR, Mullish BH, Kelly C, Fischer M. The evolution of the use of faecal microbiota transplantation and emerging therapeutic indications. *Lancet*. 2019;394(10196):420-431. doi:10.1016/S0140-6736(19)31266-8

17.Allegretti JR, Kassam Z, Mullish BH, et al. Effects of Fecal Microbiota Transplantation With Oral Capsules in Obese Patients. *Clin Gastroenterol Hepatol*. 2020;18(4):855-863.e2. doi:10.1016/j.cgh.2019.07.006

18.Fei N, Zhao L. An opportunistic pathogen isolated from the gut of an obese human causes obesity in germfree mice. *ISME J*. 2013;7(4):880-884. doi:10.1038/ismej.2012.153

19.de Groot P, Nikolic T, Pellegrini S, et al. Faecal microbiota transplantation halts progression of human new-onset type 1 diabetes in a randomised controlled trial. *Gut.* 2021;70(1):92-105. doi:10.1136/gutjnl-2020-322630

20.李阳阳, 朱昱晓, 周迎, 等. 洗涤菌群移植治疗脆性糖尿病的有效性及安全性观察[J] . 中华糖尿病杂志, 2020, 12(12) : 962-967.

21.Liu Q, Hua Y, He R, et al. Restoration of intestinal secondary bile acid synthesis: A potential approach to improve pancreatic β cell function in type 1 diabetes. *Cell Rep Med*. 2025;6(5):102130. doi:10.1016/j.xcrm.2025.102130

22. Høyer KL, Dahl Baunwall SM, Kornum DS, et al. Faecal microbiota transplantation for patients with diabetes type 1 and severe gastrointestinal neuropathy (FADIGAS): a randomised, double-blinded, placebo-controlled trial. *EClinicalMedicine*. 2024;79:103000. Published 2024 Dec 16. doi:10.1016/j.eclinm.2024.103000

23. Yang J, Yang X, Wu G, et al. Gut microbiota modulate distal symmetric polyneuropathy in patients with diabetes. *Cell Metab*. 2023;35(9):1548-1562.e7. doi:10.1016/j.cmet.2023.06.010
