# Supplementary material for: Efficacy, safety, and feasibility of youth-derived fecal microbiota transplantation among adults with type 1 diabetes mellitus: A protocol of Pilot Randomized Controlled Trial
Source: PLoS One. 2026 Sep 21;21(9):e0343078. doi: 10.1371/journal.pone.0343078 (PMC13592622; doi:10.1371/journal.pone.0343078)
Supplement: Supplementary file 3 [file pone.0343078.s004.docx]

**Efficacy and safety of** **Youth-derived Fecal Microbiota Transplantation among adults with Type 1 Diabetes Mellitus: A protocol of pilot randomized** **controlled trial**

**Leading Institution:** Zhongnan Hospital of Wuhan University

**Principal Investigator:** Zhe Dai

**Department:** Department of Endocrinology

**Research Period:** July 2025 to December 2026

**Version:** 1.0 (July 24, 2025)

**Protocol Abstract**

| **Study title** | Efficacy and safety of Youth-derived Fecal Microbiota Transplantation among adults with Type 1 Diabetes Mellitus: A protocol of pilot randomized controlled trial |
| --- | --- |
| **Study Objective** | To explore the efficacy and safety of yFMT in adults with Type 1 Diabetes Mellitus (T1DM). Through oral administration and a multi-dimensional assessment system, the study aims to verify the potential effects of yFMT on improving glycemic control, protecting islet function, enhancing insulin sensitivity, and regulating immunity and gut microbiota. |
| **Study Design** | Single-center, randomized, double-blind, parallel, placebo-controlled study. |
| **Sample Size** | 20 |
| **Participant selection** | **Inclusion Criteria:**  1.Confirmed T1DM by a professional endocrinologist with a disease duration of >1 year.  2.Age 18–60 years.  3.HbA1c 7.0–14.0% or Time in Range (TIR) < 70%.  4.Use of multiple daily injections (MDI) or insulin pump therapy for ≥3 months.  5.Total daily insulin dose ≥0.3 IU/kg and basal rate ≥0.05 IU/h.  6.Women of childbearing age are willing to use appropriate contraceptive measures.  7.Signed informed consent form. |
|  | **Exclusion Criteria：**  1.Use of any glucose-lowering medications other than insulin;  2.Continuous use (>3 days) of antibiotics, probiotic preparations, intestinal microecological modulators, or other medications with potential effects on the gut microbiota within 3 months prior to enrollment;  3.Occurrence of acute diabetic complications within 1 month prior to enrollment, including diabetic ketoacidosis (DKA) or hyperosmolar hyperglycemic state;  4.Presence of gastrointestinal diseases (e.g., persistent vomiting; confirmed or suspected gastrointestinal obstruction; short bowel syndrome; inflammatory bowel disease; active gastrointestinal bleeding; gastrointestinal perforation; high-output enteric fistula; severe diarrhea; significant fibrotic intestinal strictures; fulminant colitis or toxic megacolon), history of major gastrointestinal surgery (e.g., gastrectomy, pelvic anastomosis, colostomy, or other digestive system surgeries), malignancy, or sepsis;  5.Severe cardiovascular or cerebrovascular disease, or significant hepatic, renal, or hematopoietic disorders, or severe immunosuppression, congenital or acquired immunodeficiency;  6.Recent treatment with high-risk immunosuppressive or cytotoxic agents, such as rituximab, doxorubicin, or moderate-to-high dose systemic corticosteroids (e.g., prednisone ≥20 mg/day for more than 4 consecutive weeks);  7.Use of closed-loop insulin delivery systems or sensor-augmented insulin pumps within the past 3 months;  8.Concurrent participation in another randomized clinical trial;  9.Pregnancy or lactation, or planning pregnancy during the study period;  10.Inability to comply with the recommended intervention or follow-up schedule for any reason. |
| **Treatment** | Gut microbiota extract capsules + standard insulin therapy vs. placebo capsules + standard insulin therapy. |
| ****Study assessment**** | **Efficacy assessment:**  (1) ****Primary efficacy endpoint****  the composite achievement rate of time in range(TIR)>70%+**time below range(**TBR)<4% , assessed using **continuous glucose monitoring (CGM) metrics**.   (2) **Secondary efficacy endpoints**   **① Glycemic metabolism**  **HbA1c**: Absolute change (%) from baseline at **weeks 4 and 12** after the intervention.  **Fasting plasma glucose and 2-hour postprandial glucose**: Absolute changes (mmol/L) from baseline at **weeks 4 and 12** after the intervention.  **CGM metrics**: Absolute changes (%) from baseline in other core CGM parameters at **weeks 4 and 12** after the intervention.  **② islet β-cell function**  **Fasting C-peptide**: Absolute change (ng/mL) from baseline at **weeks 4 and 12** after the intervention.  **2-hour C-peptide during the mixed-meal tolerance test (MMT)**: Absolute change (ng/mL) from baseline at **weeks 4 and 12** after the intervention.  **Area under the curve of 2-hour C-peptide during MMT**: Absolute change from baseline at **weeks 4 and 12** after the intervention.  **③ Insulin sensitivity**  **Estimated glucose disposal rate (eGDR)**, calculated based on clinical parameters including **waist-to-hip ratio, diastolic blood pressure, and HbA1c**: Change from baseline at **weeks 4 and 12** after the intervention.  **④ Immune modulation**  **Treg/Th17 ratio**: Changes from baseline at **weeks 4 and 12** in the proportions of **CD4⁺CD25⁺FoxP3⁺ regulatory T cells (Treg)** and **CD4⁺IL-17A⁺ T helper 17 cells (Th17)** in peripheral blood.  **Diabetic autoantibody**: Changes from baseline at **weeks 4 and 12** in titers of **GADA, IAA, and ICA**, as well as the proportion of participants achieving a **≥50% reduction in antibody titers**.  **⑤ Infection and inflammatory markers**  Changes from baseline at **weeks 4 and 12** in levels of **white blood cell count (WBC), C-reactive protein (CRP), erythrocyte sedimentation rate (ESR), procalcitonin (PCT)**, and inflammatory mediators including **TNF-α, IL-1β, IL-8, IFN-γ, lipopolysaccharide antibodies, and occludin antibodies**.  **⑥ Gut microbiota**  **Changes in the abundance of key bacterial genera**: Relative abundance changes of beneficial bacteria, including **Akkermansia** and **Roseburia**, in fecal samples at **week 12** compared with baseline.  **Bile acid metabolic profiles**: Changes from baseline at **week 12** in **secondary bile acids** (including **lithocholic acid [LCA] and isolithocholic acid [isoLCA]**) and **short-chain fatty acids (SCFAs)** in blood and fecal samples.  **Epitranscriptomic profiles of microbiota-derived cfRNA**: Changes from baseline at **week 12** in **cfRNA methylation sites and patterns** in blood samples. Statistical modeling will be applied to identify **cfRNA methylation biomarkers associated with therapeutic response to yFMT**.  **⑦ Patient-reported outcomes**  **Fear of hypoglycemia**: Change from baseline at **weeks 4 and 12** in Chinese version of **Hypoglycemia Fear Survey–Worry Scale II(HFS-WSII)** scores.  **Diabetes-related distress**: Change from baseline at **weeks 4 and 12** in **Diabetes Distress Scale (DDS)** scores.  **Quality of life**: Change from baseline at **weeks 4 and 12** in **EQ-5D-5L** questionnaire scores.  ****⑧ Lipid metabolism****  Changes from baseline at **weeks 4 and 12** in levels of **total cholesterol (TC), triglycerides (TG), low-density lipoprotein cholesterol (LDL-c), and high-density lipoprotein cholesterol (HDL-c)**.  ****⑨ Insulin dose****  **Total daily insulin dose (TDD)**: Change from baseline at **weeks 4 and 12** (units/day). |
|  | ****Safety assessment****  **①Incidence of adverse events (AEs)**: Adverse events will be recorded and graded according to the **Common Terminology Criteria for Adverse Events (CTCAE), version 5.0**.  **②Incidence of serious adverse events (SAEs)**: Including **severe hypoglycemia, diabetic ketoacidosis (DKA)**, and other SAEs, defined as medical events that result in **hospitalization or prolonged hospitalization, disability, impaired ability to work, life-threatening conditions, death, or congenital anomalies**. |
| **Statistics and analysis** | **All efficacy endpoints will be analyzed based on the intention-to-treat (ITT) population. Participants who do not complete the final treatment assessment will be included in the analysis and classified as non-responders. Safety endpoints will be analyzed in the safety analysis set.Key continuous glucose monitoring (CGM) metrics, as recommended by international expert consensus, will be calculated using Glyculator 2.0 software (Medical University of Lodz, Poland).**  **For continuous variables, normality will be assessed using the Shapiro–Wilk test. Data following a normal distribution will be expressed as mean ± standard deviation and analyzed using the independent samples t-test (for between-group comparisons) or the paired t-test (for within-group comparisons). Non-normally distributed data will be expressed as median (Q1, Q3) and analyzed using the Mann–Whitney U test (for between-group comparisons) or the Wilcoxon signed-rank test (for within-group comparisons). Categorical data will be expressed as number (percentage) and analyzed using Fisher’s exact test. The primary efficacy endpoint (composite glycaemic target achievement rate) and categorical secondary endpoints will be analyzed using generalized estimating equations (GEE) to assess the effects of group, time, and their interaction. Continuous secondary endpoints (e.g., changes in HbA1c and C-peptide) will be analyzed using a linear mixed-effects model, including fixed effects for group, time, their interaction, and baseline value, and a random effect for subject. Safety outcomes (incidence of adverse events) will be compared using Fisher’s exact test. Logistic regression analysis will be used to explore factors associated with response to yFMT treatment. Missing data for the primary endpoint will be handled using the last observation carried forward (LOCF) method. All statistical analyses will be performed using R software (version 4.3.2) and GraphPad Prism 9.0. All tests will be two-sided, and a P-value < 0.05 will be considered statistically significant. Bonferroni correction will be applied for post hoc multiple comparisons.** |
| ****Study period**** | **July 2025 - December 2026 (1.5 years)** |

**1.Background**

### **1.1 Research Background and Significance**

**1.1.1 The incidence of Type 1 Diabetes in China shows an increasing trend, and patients' glycemic control remains suboptimal**

Type 1 Diabetes Mellitus (T1DM) requires lifelong dependence on insulin therapy; it can occur at any age but most commonly presents during childhood and adolescence^[1]^. The latest survey on T1DM incidence in China shows that the incidence across all age groups is 1.01 per 100,000, while the incidence rates for the age groups 0-14, 15-29, and 30 and above are 1.93, 1.28, and 0.69 per 100,000, respectively^[2]^. According to these latest data, there are 13,000 newly diagnosed cases of T1DM in China each year, ranking the country first in the Western Pacific region in terms of the number of new cases. Compared with previous epidemiological data^[3]^ (where the incidence rate of T1DM in children under 14 was 0.59 per 100,000), T1DM in China is increasing rapidly; there is an urgent need to strengthen attention and related research in this field.

Education, nutrition, exercise, blood glucose monitoring, and insulin therapy are the “five-horse carriage” of comprehensive management for T1DM^[4]^. In recent years, although new insulin formulations, continuous glucose monitoring (CGM) systems, and novel insulin pumps have provided better means for blood glucose monitoring and the treatment of T1DM. However, relevant domestic and international data suggest that T1DM management still faces multiple challenges, such as high requirements for patients' self-management abilities, poor overall glycemic control in the population, and frequent occurrences of hypoglycemia^[5-10]^. Relevant measures are urgently needed to improve these adverse current conditions.

**1.1.2 Gut microbiota dysbiosis plays a key role in the pathogenesis of T1DM**

The core pathological mechanism of T1DM is the autoimmune-mediated destruction of pancreatic beta cells. Although traditional insulin therapy can control blood glucose, it cannot block the autoimmune process or restore islet β-cell function. In recent years, there has been an increasing awareness of the critical role of the gut microbiota as a “second genome” in the pathogenesis of T1DM^[11]^. Numerous studies have shown that T1DM patients exhibit characteristic changes in gut microbiota, including a decrease in butyrate-producing bacteria, a decline in secondary bile acids (such as LCA and isoLCA), and an increase in pathogenic bacteria (with a decreased Bacteroidetes/Firmicutes ratio). This is accompanied by impaired intestinal barrier function, forming a disrupted “microbiota-gut-islet axis” ^[12,13]^.

**1.1.3 Fecal Microbiota Transplantation offers new hope for the treatment of T1DM**

Fecal Microbiota Transplantation (FMT) is a disruptive technology that reconstructs micro-ecological balance by transplanting the gut microbiota of a healthy donor into the patient's intestinal tract. Animal studies have confirmed that adjusting the structure of the gut microbiota can improve or delay T1DM^[14,15]^. Therefore, FMT has the potential to treat T1DM, potentially not only altering insulin sensitivity but also modifying the course of T1DM by attenuating underlying autoimmunity. Its potential pathways of action include: (1) restoring short-chain fatty acid (SCFA)-producing microbiota to improve intestinal barrier function^[16]^; (2) modulating autoimmune responses^[17]^; (3) regulating metabolism through the gut-brain axis; and (4) inhibiting the overgrowth of pathogenic bacteria such as Enterobacteriaceae^[18]^.

### **1.2 Research Status**

**1.2.1 Research progress of FMT in the treatment of T1DM**

Currently, evidence for the application of FMT in T1DM patients remains relatively scarce. One RCT compared the efficacy of autologous FMT versus allogeneic FMT from healthy donors in delaying the decline of pancreatic beta-cell function^[19]^. The study showed that the rate of beta-cell decline in the allogeneic FMT group appeared to be slower than that observed in several other clinical trials; however, after receiving 12 doses of autologous FMT (rather than FMT from healthy donors), the rate of beta-cell decline was preserved for 3 months, and glycemic control improved. Metagenomic analysis found that changes in microbiota such as *Parabacteroides distasonis* were correlated with efficacy.The team led by Yu Liu confirmed that Washed Microbiota Transplantation (WMT) can significantly improve blood glucose fluctuations (reducing MAGE and CV) in brittle diabetes patients with complete loss of islet function and improve treatment satisfaction^[20]^. The team further discovered that WMT can increase levels of LCA and isoLCA, which is associated with the improvement of islet function^[21]^. In the above studies, the method of microbiota transplantation was liquid infusion via a nasojejunal tube.Additionally, two RCTs compared FMT capsules with placebo capsules regarding safety and efficacy in T1DM patients with severe gastrointestinal neuropathy^[22]^ and those with diabetic peripheral neuropathy^[23]^. Oral FMT capsules improved gastrointestinal symptoms and quality of life in T1DM patients with intestinal symptoms and significantly improved the severity of peripheral neuropathy. The above studies all reported a good safety profile for fecal microbiota transplantation, with the primary adverse events being mild gastrointestinal reactions^[19-23]^. The most common adverse events include nausea, abdominal pain, diarrhea, abdominal distension, fatigue, constipation, and decreased appetite.

**1.2.2 Research Gap**

Although existing studies have preliminarily demonstrated the potential value of fecal microbiota transplantation (FMT) in the treatment of type 1 diabetes mellitus (T1DM), several key scientific issues remain unresolved:

(1) Limited therapeutic targets：

1.Most current studies focus primarily on the protective effects of FMT on residual pancreatic β-cell function, with heterogeneous and inconsistent findings;

2.Systematic evaluations of FMT-mediated modulation of autoimmunity (e.g., changes in islet-related autoantibody titers) and improvements in insulin sensitivity are lacking.

(2) Inadequate efficacy evaluation framework：

1.Limited glycemic metrics: The majority of studies rely on traditional indicators such as glycated hemoglobin (HbA1c) and fasting plasma glucose, or isolated continuous glucose monitoring (CGM) parameters (e.g., time in range [TIR] or time below range [TBR]);

2.Lack of a composite “dual-target” assessment (TIR >70% combined with TBR <4%), which limits comprehensive evaluation of both the quantity and quality of glycemic control.

(3) Insufficient standardization of FMT technology：

1.Absence of a gold standard for donor selection: Multidimensional factors such as metabolic characteristics, microbial composition, and genetic background have not been systematically considered;

2.Substantial variability in transplantation procedures: Key parameters, including microbiota preparation methods and routes of administration, lack unified standards.

(4) Barriers to clinical application：

1.Most existing studies have used nasojejunal tube administration, which is technically demanding and poorly tolerated by patients;

2.Oral FMT capsules offer greater convenience by avoiding invasive procedures and reducing the logistical burden of repeated hospital visits for both donors and recipients, while being associated with fewer adverse events.

To address these limitations, the present study proposes, for the first time, the application of a “third-generation FMT technology”—youth-derived fecal microbiota transplantation (yFMT)—administered orally in capsule form. This small-sample, randomized, double-blind, parallel, placebo-controlled pilot study aims to systematically evaluate the multidimensional effects of yFMT in adults with T1DM, including its impact on glycemic control (assessed using a “dual-target” composite endpoint), pancreatic function (fasting C-peptide, 2-hour mixed-meal tolerance test [MMT] C-peptide and its area under the curve), insulin sensitivity (estimated glucose disposal rate, eGDR), and cellular immune status (titers of GADA, IAA, and ICA). The findings are expected to provide a novel paradigm for microbiota-based therapy in T1DM.

The yFMT technology offers three major innovative advantages:①Precision donor system: Strict age restriction (4–17 years) to capture the peak period of gut microbiota diversity, combined with a three-dimensional matching framework based on disease status, sex, and age;② Quality control innovation: Dual-platform donor screening using 16S rRNA sequencing and metagenomic analysis, together with a donor–recipient dynamic matching algorithm;③Process optimization: Adoption of low-temperature freeze-drying encapsulation technology to preserve microbial viability, and implementation of stringent criteria for harmful microorganism elimination (<0.1%).

**2. Objectives**

1. Primary Objective: To evaluate the efficacy and safety of yFMT on glycemic control in adults with T1DM.

2. Secondary Objectives:

① To explore the protective effect of yFMT on residual pancreatic β-cell function.

② To investigate the regulatory effect of yFMT on insulin sensitivity.

③ To analyze the regulation of cellular immunity by yFMT.

④ To discuss the impact of yFMT on gut microbiota and its metabolites.

⑤ Exploratory analysis: To screen for microbial biomarkers that predict the therapeutic efficacy of yFMT.

**3. Study Design and Procedures**

**3.1 Study Design**

This study is a small-sample, single-center, randomized, double-blind, parallel, placebo-controlled pilot study conducted at Zhongnan Hospital of Wuhan University, aimed at preliminarily assessing the efficacy and safety of yFMT in adult T1DM patients. The study includes a 17-day lead-in period, a 6-day treatment period, and a 3-month follow-up period.During the lead-in period (Day -17 to Day -3), insulin dosages may be optimized, and dietary guidance will be provided according to the Dietary Guidelines for Diabetes in China (2017). Subjects will wear a CGM system, and CGM data will be collected as baseline glycemic data. From Day -3 to Day 0 of the lead-in period, hospitalization is required for oral rifaximin administration and bowel preparation. Following the lead-in period, eligible subjects will be randomized in a 1:1 ratio into either the intervention group or the control group. The intervention group will receive capsules containing gut microbiota extract from the fresh feces of healthy donors (6 capsules twice daily for 6 consecutive days). The control group will receive placebo capsules (with the same dosage and frequency as the microbiota extract capsules). Both groups will continue their routine insulin therapy (maintaining their previous insulin regimens). Safety and efficacy evaluations will be performed at Weeks 4 and 12 post-intervention. Unblinding will occur after the primary endpoint assessment (at the end of the 3-month follow-up). For ethical considerations, participants in the placebo group will be offered a one-time free yFMT treatment after unblinding, following the same protocol as the intervention group (6 capsules twice daily for 6 days). This ensures that all participants have the opportunity to benefit, though participation remains voluntary. The study protocol has been reviewed and approved by the Ethics Committee of Zhongnan Hospital of Wuhan University. Prior to the trial, researchers will explain the nature, purpose, procedures, potential benefits, and risks of the trial to the subjects, who must sign an informed consent form. The consent form will explicitly state that participants assigned to the placebo group will have the opportunity to receive free yFMT after the study concludes.

**3.2 Randomization**

After completing the run-in period, all eligible subjects will be randomized 1:1 into the intervention or control group. The allocation process will utilize a computer-generated permuted block randomization code (prepared in advance by a statistical expert using R software and assembled into sealed, opaque envelopes in order by an independent researcher). To minimize selection bias, randomization codes will be assigned by an independent researcher. Once a qualified subject is enrolled, the principal investigator will notify the independent researcher to open the sealed envelopes in sequence to execute the allocation.

**3.3 Blinding**

This study employs a double-blind, single-dummy design, using a placebo for the gut microbiota extract capsules as a control. The appearance, shape, odor, and specifications of the placebo are identical to those of the active capsules. Experimental drugs will be randomized and coded according to the clinical trial randomization scheme. Researchers must strictly follow the medication codes when dispensing drugs and are not permitted to select medications; the codes will remain stable throughout the entire trial. For any subject who drops out or is withdrawn from the trial after randomization, their randomization number cannot be reused for other patients, regardless of whether medication was administered. To ensure objectivity in endpoint assessment, both the subjects and the personnel evaluating the endpoints will be blinded. Electronic blinding codes will be held by a non-blinded statistician and will not be unblinded until the study is completed.

**3.4 Sample Size Calculation**

As there are no existing reports on the composite glycemic target achievement rate (TIR > 70% + TBR < 4%) for yFMT in the field of T1DM, the sample size cannot be formally calculated. Drawing on the sample sizes of previous pilot studies (n = 10–20), this study intends to include 20 eligible adult T1DM patients, with 10 cases each in the intervention and control groups.

**3.5 Study Procedures**

**Abbreviations:** T1DM, Type 1 diabetes mellitus; ICF, Informed consent form; CGM, Continuous glucose monitoring; TDD, Total daily insulin dose; BP, Blood pressure; P, Pulse; BMI, Body mass index; WHR, Waist-to-hip ratio; HbA1c, Glycated hemoglobin; FPG, Fasting plasma glucose; MMTT 2h-PG, 2-hour plasma glucose during the mixed-meal tolerance test; MMTT 2h-CP, 2-hour C-peptide during the mixed-meal tolerance test; yFMT, yFMT, Youth-derived Fecal Microbiota Transplantation; BID, Twice daily; DKA, Diabetic ketoacidosis; ADEs, Adverse drug events; cfRNA, Cell-free RNA.

**4. Participant selection**

**4.1 Inclusion criteria**

①A diagnosis of T1DM confirmed by an endocrinologist, with a disease duration of ≥1 year;

②Age between 18 and 60 years;

③HbA1c between 7.0% and 14.0% or TIR <70%;

④Treatment with multiple daily injections (MDI) or continuous subcutaneous insulin infusion (CSII) for at least 3 months prior to enrollment;

⑤TDD ≥0.3 IU/kg/day before admission, with a basal insulin rate ≥0.05 IU/hour;

⑥Women of childbearing potential willing to use appropriate contraceptive measures during the study period;

⑦Provision of written informed consent.

**4.2 Exclusion criteria**

①Use of any glucose-lowering medications other than insulin;

②Continuous use (>3 days) of antibiotics, probiotic preparations, intestinal microecological modulators, or other medications with potential effects on the gut microbiota within 3 months prior to enrollment;

③Occurrence of acute diabetic complications within 1 month prior to enrollment, including diabetic ketoacidosis (DKA) or hyperosmolar hyperglycemic state;

④Presence of gastrointestinal diseases (e.g., persistent vomiting; confirmed or suspected gastrointestinal obstruction; short bowel syndrome; inflammatory bowel disease; active gastrointestinal bleeding; gastrointestinal perforation; high-output enteric fistula; severe diarrhea; significant fibrotic intestinal strictures; fulminant colitis or toxic megacolon), history of major gastrointestinal surgery (e.g., gastrectomy, pelvic anastomosis, colostomy, or other digestive system surgeries), malignancy, or sepsis;

⑤Severe cardiovascular or cerebrovascular disease, or significant hepatic, renal, or hematopoietic disorders, or severe immunosuppression, congenital or acquired immunodeficiency;

⑥Recent treatment with high-risk immunosuppressive or cytotoxic agents, such as rituximab, doxorubicin, or moderate-to-high dose systemic corticosteroids (e.g., prednisone ≥20 mg/day for more than 4 consecutive weeks);

⑦Use of closed-loop insulin delivery systems or sensor-augmented insulin pumps within the past 3 months;

⑧Concurrent participation in another randomized clinical trial;

⑨Pregnancy or lactation, or planning pregnancy during the study period;

⑩Inability to comply with the recommended intervention or follow-up schedule for any reason.

**4.3 Withdrawal criteria**

**4.3.1 Violation of inclusion criteria (erroneous enrollment)**

a. Retrospective identification of failure to meet key inclusion criteria (e.g., actual disease duration <1 year, concomitant use of non-insulin glucose-lowering agents);

b. Severe missing baseline data (e.g., failure to complete CGM monitoring, absence of blood or stool sample collection).

**4.3.2 Serious compliance issues**

a. Failure to complete yFMT intervention (capsule intake <50% of the prescribed dose);

b. Unauthorized changes to treatment during the run-in or intervention period (e.g., self-administration of antibiotics);

c. Major deviation from dietary recommendations (e.g., prolonged high-fat or high-sugar diet affecting microbial engraftment).

**4.3.3 Unreliable or missing data**

a. Missing ≥50% of key outcome data (e.g., TIR, C-peptide) due to refusal of follow-up or device malfunction;

b. Evidence of data fabrication or serious recording errors (e.g., intentional modification of glucose logs).

**4.3.4 Other factors**

a. Loss to follow-up (failure to complete ≥2 scheduled visits);

b. Withdrawal due to non–study-related conditions (e.g., accidental fracture, hospitalization for acute non-diabetic illness).

**4.4 Study termination criteria**

**4.4.1 Safety-related reasons**

a. Occurrence of serious adverse events (SAEs), such as DKA, severe hypoglycemia, or severe allergic reactions;

b. Persistent grade ≥3 gastrointestinal adverse events (e.g., severe diarrhea, vomiting, abdominal pain) unresponsive to symptomatic treatment;

c. New-onset autoimmune disease or worsening of pre-existing autoimmune conditions (e.g., thyroiditis, celiac disease).

**4.4.2 Poor adherence or voluntary withdrawal**

a. Failure to complete the yFMT intervention as per protocol (e.g., missed ≥50% of doses);

b. Participant request to withdraw from the study (e.g., inability to continue follow-up for personal reasons).

**4.4.3 Other medical reasons**

a. Pregnancy or planned pregnancy (as the effects of yFMT on the fetus are unknown);

b. Newly diagnosed conditions requiring exclusion (e.g., inflammatory bowel disease, malignancy);

c. Any other condition deemed by the investigators to necessitate withdrawal from the study.

**5. Study methods**

**5.1 Investigational regimen and specifications**

(1) Regimen in the intervention group: Gut microbiota extract capsules

Specification: Each capsule contains ≥4 × 10¹³ viable microorganisms

Source: Human-derived active intestinal microbiota obtained from rigorously screened healthy donors

Manufacturer: Meiyitian Biopharmaceutical Co., Ltd. (Wuhan, China)

Shelf life and storage conditions:Stored at −80 ± 2 °C: shelf life of 6 months, Stored at −20 ± 2 °C: shelf life of 4 weeks

Capsules should be thawed at room temperature and swallowed within 20 minutes after thawing

(2) Regimen in the control group: Gut microbiota extract placebo capsules

Composition: Skim milk, starch, and trace amounts of protective excipients

Manufacturer: Meiyitian Biopharmaceutical Co., Ltd. (Wuhan, China)

Shelf life and storage conditions:Stored at −80 ± 2 °C: shelf life of 6 months, Stored at −20 ± 2 °C: shelf life of 4 weeks

Capsules should be thawed at room temperature and swallowed within 20 minutes after thawing

The placebo capsules are manufactured to be indistinguishable from the active gut microbiota extract capsules in terms of appearance, size, color, dosage form, weight, taste, and odor. They are composed of pharmacologically inert substances without known therapeutic or toxic effects. If any adverse events related to the investigational products are identified during the study, the research team will provide prompt medical management and close follow-up to ensure participant safety.

**5.2. Treatment**

(1) Gut microbiota extract capsules or placebo capsules

Route of administration: Oral

Method of administration: Capsules should be swallowed whole without chewing or breaking; fasting is required for 2 hours before and after administration

Single dose: 6 capsules (one blister pack) per dose

Dosing frequency: Twice daily (once in the morning and once in the evening, preferably at fixed times)

Treatment duration: 6 consecutive days (total dose: 72 capsules)

Capsule sequence: White capsules are administered during the first 3 days, followed by white–green bicolor capsules during the subsequent 3 days (capsules with an “M” suffix code)

Timing of administration: On an empty stomach in the morning and evening to minimize the impact of gastric acid on microbial viability

(2) Standard insulin therapy

All participants will continue their usual insulin treatment regimens, including insulin pump therapy or multiple daily insulin injections. Insulin doses will be adjusted according to glucose monitoring results, and all dose adjustments must be documented. Detailed records of insulin use—including dose, timing, formulation, and any modifications—will be maintained, along with the rationale for adjustments and corresponding outcomes.

**5.3 Precautions and supportive measures**

Dietary preparation: Participants are required to follow a semi-liquid diet for one week prior to capsule administration. In addition, a 3-day course of antibiotics (rifaximin) will be administered before treatment, followed by bowel cleansing with polyethylene glycol electrolyte solution.

Storage and handling: Capsules should be stored at −80 °C and thawed at room temperature for 10 minutes prior to use. The capsules must be ingested within 20 minutes after thawing.

Management of adverse events:Mild abdominal bloating or diarrhea: observation only; treatment discontinuation is not required.

Severe abdominal pain or bloody stools: immediate discontinuation of the investigational product and prompt medical evaluation.

Dietary recommendations during intervention: Spicy foods should be avoided, and wheat bran–containing foods are prohibited during the intervention period.

Physical activity: In the absence of contraindications or comorbidities, participants are encouraged to engage in moderate-intensity aerobic exercise 3–5 times per week, with each session lasting at least 30 minutes, for a total weekly duration of 150–250 minutes. Recommended activities include brisk walking and stationary cycling.

**5.4 Concomitant medications**

(1) Permitted concomitant medications

1. Symptomatic treatments (indication and frequency must be recorded):

Gastrointestinal symptoms: diosmectite (for diarrhea), omeprazole (for gastric acid hypersecretion)

Allergy/inflammation: loratadine (non-sedating antihistamine), topical corticosteroids (e.g., hydrocortisone cream)

Analgesics: acetaminophen (nonsteroidal anti-inflammatory drugs are prohibited)

2. Maintenance medications for chronic conditions (stable dose for ≥4 weeks):

Antihypertensive agents (angiotensin-converting enzyme inhibitors or angiotensin receptor blockers preferred)

Thyroid hormone replacement therapy (e.g., levothyroxine sodium)

(2) Prohibited concomitant medications

1. Medications affecting the gut microbiota:

Antibiotics (e.g., penicillins, cephalosporins, macrolides)

Laxatives (e.g., polyethylene glycol, senna)

2.Immunomodulatory agents:

Systemic corticosteroids (prednisone ≥5 mg/day)

Immunosuppressive agents (e.g., methotrexate, cyclosporine)

3.Other glucose-lowering therapies:

Oral antidiabetic agents (e.g., metformin, SGLT-2 inhibitors)

GLP-1 receptor agonists

4.Non-essential traditional Chinese medicines or dietary supplements:

Herbal medicines containing laxative or antimicrobial components (e.g., rhubarb, Coptis chinensis)

Enzyme preparations or fiber supplements

(3) Medications requiring temporary suspension of the study intervention

1. Temporary use of prohibited medications (e.g., antibiotics for acute infections):

Study intervention will be suspended and recorded as an “interruption.”

After infection resolution, gut microbiota re-evaluation (stool testing) is required, and continuation of the study is permitted only if eligibility criteria are met.

2. Emergency medications (e.g., anesthetics required for surgery):

Must be reported to the ethics committee for documentation. A formal assessment will be conducted to determine whether study withdrawal is necessary.

**6. Study visits**

**6.1 Screening visit (Day −17): outpatient setting**

1. Provision and signing of electronic informed consent;

2. Collection of demographic characteristics, including age, sex, ethnicity, height, body weight, waist circumference, hip circumference, duration of T1DM, past medical history, and medication history, as well as lifestyle information (smoking, alcohol consumption, diet, and physical activity);

3. Assessment of glycemic status:HbA1c values obtained within the past 3 months (required range: 7.0–14.0%), or CGM data collected within the past month showing TIR <70%. If neither is available, HbA1c will be measured at screening;
4. Assessment of pancreatic function: fasting C-peptide levels obtained within the past 3 months;
5. Verification of exclusion criteria, including recent use of antibiotics or probiotics and the presence of concomitant autoimmune diseases;
6. Eligible participants will be provided with a Sinocare continuous glucose monitoring (CGM) device.

**6.2 Run-in period (Day −17 to Day 0)**

(1) Outpatient phase (Day −17 to Day −3)

1. Continuous glucose monitoring for 14 consecutive days;

2. Insulin dose optimization to achieve a stable regimen;

3. Standardized diet according to the Chinese Dietary Guidelines for Diabetes.

(2) Inpatient phase (Day −3 to Day 0)

Oral rifaximin at a dose of 0.2 g per administration, four times daily, for 3 consecutive days; On the evening of Day −3, bowel preparation will be initiated using polyethylene glycol electrolyte solution.

**6.3 Baseline visit (Day −3)**

(1) Clinical assessment: blood pressure, pulse, height, body weight, waist and hip circumferences; calculation of body mass index (BMI) and waist-to-hip ratio (WHR); total daily insulin dose (TDD, U/kg); and documentation of concomitant medications.

(2) Laboratory assessments:

①Metabolic parameters:

Glycemic metabolism: fasting plasma glucose, 2-hour glucose during mixed-meal tolerance test (MMT-2h glucose), and HbA1c;

Lipid metabolism: total cholesterol (TC), triglycerides (TG), low-density lipoprotein cholesterol (LDL-c), and high-density lipoprotein cholesterol (HDL-c).

②ilet β-cell function:

fasting C-peptide, 2-hour C-peptide during MMT, and the area under curve(AUC) of 2h C peptide of MMT;

③Insulin sensitivity: estimated glucose disposal rate (eGDR), calculated based on clinical parameters such as diastolic blood pressure(DBP), WHR, and HbA1c;

④Infection and inflammation markers: complete blood count, erythrocyte sedimentation rate (ESR), C-reactive protein (CRP), procalcitonin (PCT), and inflammatory mediators including TNF-α, IL-1β, IL-8, IFN-γ, lipopolysaccharide antibodies, and zonulin antibodies;

⑤Cellular and humoral immune markers: titers of GADA, IAA, and ICA, and proportions of peripheral blood Treg and Th17 cells;

⑥Gut microecology: gut microbiota composition, plasma and fecal metabolomic profiles, and circulating cell-free RNA (cfRNA);

⑦Baseline safety assessments: liver function, renal function, and a comprehensive thyroid function panel.

(3) Collection and analysis of CGM data obtained during the 2-week run-in period (Day −17 to Day −3);
(4) Completion of validated questionnaires, including the simplified Chinese (Mainland China) versions of the EQ-5D-5L, CHFS-WSII, and DDS.

4. Intervention period (Day 0 to Day 6): inpatient setting

(1) Intervention: administration of gut microbiota extract capsules or placebo capsules at a dose of 6 capsules per administration, twice daily, for 6 consecutive days;

(2) Daily monitoring: gastrointestinal adverse events (diarrhea, abdominal pain, vomiting) and glycemic variability, with real-time CGM monitoring and alerts.

5. Mid-study visit (Week 4 ± 3 days): outpatient setting

(1) Clinical assessment: blood pressure, pulse, height, body weight, waist and hip circumferences; calculation of BMI and WHR; TDD (U/kg); and documentation of concomitant medications;

(2) Laboratory assessments:

①Metabolic parameters:

Glycemic metabolism: fasting plasma glucose, MMT-2h glucose, and HbA1c;

Lipid metabolism: TC, TG, LDL-c, and HDL-c;

②ilet β-cell function: fasting C-peptide, 2-hour C-peptide during MMT, and AUC of 2h C-peptide of MMT;

③Insulin sensitivity: eGDR;

④Immune parameters: titers of GADA, IAA, and ICA, and proportions of peripheral blood Treg and Th17 cells.

(3) Collection and analysis of CGM data from Weeks 2 to 4 as an early efficacy assessment;
(4) Completion of questionnaires including EQ-5D-5L, CHFS-WSII, and DDS;
(5) Collection and documentation of adverse drug events (ADEs) and serious adverse events (SAEs).

6. End-of-study visit (Week 12 ± 3 days): outpatient setting

(1) Clinical assessment: blood pressure, pulse, height, body weight, waist and hip circumferences; calculation of BMI and WHR; TDD (U/kg); and documentation of concomitant medications;

(2) Laboratory assessments:

①Metabolic parameters: Glycemic metabolism: fasting plasma glucose, MMT-2h glucose, and HbA1c; Lipid metabolism: TC, TG, LDL-c, and HDL-c;

②ilet β-cell function: fasting C-peptide, 2-hour C-peptide during MMT, and AUC of 2h C-peptide of MMT;

③Insulin sensitivity: eGDR;

④Infection and inflammation markers:

Routine tests: complete blood count, ESR, CRP, and PCT;

Inflammatory mediators: TNF-α, IL-1β, IL-8, IFN-γ, lipopolysaccharide antibodies, and zonulin antibodies;

⑤Immune markers: titers of GADA, IAA, and ICA, and proportions of peripheral blood Treg and Th17 cells;

⑥Gut microecology: gut microbiota composition, plasma and fecal metabolomic profiles, and microbial-derived cfRNA in blood samples;

⑦Safety assessments: liver function, renal function, and comprehensive thyroid function tests.

(3) Collection and analysis of CGM data from Weeks 10 to 12;
(4) Completion of questionnaires including EQ-5D-5L, CHFS-WSII, and DDS;
(5) Collection and documentation of ADEs and SAEs;
(6) Unblinding and post-study arrangements

Study unblinding will be performed. After unblinding, participants in the placebo group will be offered the option to receive one course of free yFMT treatment, on a voluntary basis. This measure is implemented for ethical considerations to ensure that all participants have the opportunity to benefit from the potential therapeutic effects.

The yFMT regimen will be identical to that used in the intervention group (6 capsules twice daily for 6 consecutive days). No additional scheduled follow-up visits will be conducted after completion of the yFMT course; however, the research team will provide necessary medical support and guidance to ensure participant safety and well-being.

Participants will be instructed on how to report any adverse events, and the research team will closely monitor and document all ADEs and SAEs to ensure participant safety.

**Study Visit Schedule.**

| **Process** | **Screening period** | **Run-in period 1** | **Baseline period** | **Run-in period 2** | **Intervention period** | **Mid-term follow-up** | **Final follow-up** |
| --- | --- | --- | --- | --- | --- | --- | --- |
|  | **Day -17** | **Day -17 to Day -3** | **Day -3** | **Day -3 to Day 0** | **Day 0 to Day 6** | **Week 4 ± 3 days** | **Week 12 ± 3 days** |
| Obtain informed consent | **√** |  |  |  |  |  |  |
| Basic characteristics | **√** |  |  |  |  |  |  |
| Lifestyle survey |  |  | **√** |  |  |  |  |
| Record anthropometric measurements |  |  | **√** |  |  | **√** | **√** |
| Average total daily insulin dose (Recent 1-2 Weeks) |  |  | **√** |  |  | **√** | **√** |
| Glucose + C-peptide（Fasting and MMT 2h） |  |  | **√** |  |  | **√** | **√** |
| HbA1c | **√** |  | **√** |  |  | **√** | **√** |
| Islet autoantibody titer |  |  | **√** |  |  | **√** | **√** |
| Treg/Th17 ratio |  |  | **√** |  |  | **√** | **√** |
| Lipids profile |  |  | **√** |  |  | **√** | **√** |
| Liver function |  |  | **√** |  |  |  | **√** |
| Renal function |  |  | **√** |  |  |  | **√** |
| Thyroid function (five items) |  |  | **√** |  |  |  | **√** |
| Infection markers and inflammatory cytokines |  |  | **√** |  |  | **√** | **√** |
| Safety assessments |  |  | **√** |  | **√** | **√** | **√** |
| AEs and SAEs |  |  | **√** |  |  | **√** | **√** |
| Gut microbiome |  |  | **√** |  |  | **√** | **√** |
| Serum metabolomics |  |  | **√** |  |  |  | **√** |
| Oral + bowel cleansing |  |  |  | **√** |  |  |  |
| Oral yFMT capsules |  |  |  |  | **√** |  |  |
| Standard insulin therapy |  | **√** | **√** | **√** | **√** | **√** | **√** |
| CGM monitoring |  | **√** |  |  | **√** | **√** | **√** |
| Questionnaires |  |  | **√** |  |  | **√** | **√** |
| Provision of yFMT to the placebo group after unblinding |  |  |  |  |  |  | **√** |

**Note:** Basic characteristics: age, sex, ethnicity, height, weight, waist circumference, hip circumference, duration of T1DM, past medical history, and medication history; Lifestyle survey: smoking, alcohol consumption, diet, and physical activity; Record anthropometric measurements: height, weight, blood pressure, pulse, waist circumference, hip circumference, BMI, and WHR; Infection markers: WBC, ESR, CRP, PCT; Inflammatory cytokines: TNF-α, IL-1β, IL-6, IL-8, IFN-γ; Safety assessments: body temperature, pulse, respiration, and blood pressure; Adverse events and serious adverse events: including abnormalities in liver and kidney function tests, as well as symptoms such as nausea, abdominal pain, diarrhea, abdominal distension, fatigue, constipation, loss of appetite, and allergic reactions. SAEs refer to medical events occurring during the clinical trial that result in hospitalization or prolonged hospitalization, disability, impaired work capacity, life-threatening conditions, death, or congenital malformations; Completion of relevant questionnaires: Simplified Chinese version (Mainland China) of the EQ-5D-5L scale, Chinese version of the Hypoglycemia Fear Survey-Worry Subscale (HFS-WS), and the Diabetes Distress Scale (DDS).

**Abbreviations:** MMT 2h, Mixed-Meal Tolerance Test, 2 hours; HbA1c, glycated hemoglobin; Treg, Regulatory T cell; Th17, T helper 17 cells; yFMT, youth-derived Fecal Microbiota Transplantation; CGM, Continuous Glucose Monitoring; T1DM, Type 1 Diabetes Mellitus; BMI, Body Mass Index; WHR, Waist-to-Hip Ratio; WBC, White Blood Cell; ESR, Erythrocyte Sedimentation Rate; CRP, C-reactive Protein; PCT, Procalcitonin; TNF-α, Tumor Necrosis Factor-alpha; IL-1β, Interleukin-1β; IL-6, Interleukin-6; IL-8, Interleukin-8; IFN-γ, Interferon gamma.

**7. Measurement and analytical methods**

**7.1 CGM**

Sinocare CGM system will be provided and keeep same for all participants .Raw CGM data will be extracted and analyzed using Glyculator 2.0 software to assess overall glycemic control and glucose variability.

**7.2 Immune cell analysis**

Flow cytometry will be used to quantify the proportions of regulatory T cells (Tregs; CD4⁺CD25⁺FoxP3⁺) and T helper 17 cells (Th17; CD4⁺IL-17A⁺) in peripheral blood samples.

Changes in immune cell subsets will be evaluated to investigate the immunomodulatory effects of yFMT on cellular immunity.

**7.3 Autoantibody assessment**

Titers of GADA,IAA, and ICA will be measured using radioimmunoassay techniques. Longitudinal changes in autoantibody levels will be analyzed to explore the impact of yFMT on pancreatic autoimmunity.

**7.4 Gut microbiota analysis**

Fecal samples will be collected from participants and subjected to gut microbiome profiling using 16S rRNA gene sequencing. Particular attention will be paid to changes in the relative abundance of key bacterial genera, including Akkermansia and Roseburia, to evaluate the effects of yFMT on gut microbial composition.

**7.5 Targeted metabolomic analysis**

Targeted metabolomics approaches will be applied to quantify concentrations of secondary bile acids (including LCA and isoLCA) and short-chain fatty acids (SCFAs; e.g., acetate, propionate, and butyrate) in blood and fecal samples. Metabolic alterations will be assessed to elucidate the effects of yFMT on bile acid metabolism and SCFAs pathways.

**7.6 Methylation sequencing of cfRNA**

cfRNA will be extracted from collected blood samples and analyzed using Low-Input Multiple Methylation Sequencing (LIME-seq) or related technologies to characterize cfRNA methylation patterns. Changes in cfRNA methylation profiles will be evaluated to explore their potential as predictive biomarkers of therapeutic response to yFMT.

**8. Efficacy and safety assessment criteria**

**8.1 Efficacy evaluation**

(1) Primary efficacy endpoint

Change in the composite glycemic target achievement rate at Weeks 4 and 12 compared with baseline, defined as simultaneous attainment of TIR >70% and TBR<4%.

(2) Secondary efficacy endpoints

①Glycemic metabolism

HbA1c: absolute change (%) from baseline at Weeks 4 and 12;

Fasting plasma glucose and 2-hour postprandial glucose: absolute changes (mmol/L) from baseline at Weeks 4 and 12;

Core CGM metrics: absolute changes (%) from baseline at Weeks 4 and 12 in additional CGM-derived parameters.

②Assessment of ilet β-cell function

Fasting C-peptide: absolute change (ng/mL) from baseline at Weeks 4 and 12;

2-hour C-peptide during MMT: absolute change (ng/mL) from baseline at Weeks 4 and 12;

AUC of 2h C-peptide of MMT: change from baseline at Weeks 4 and 12.

③Insulin sensitivity

Change in eGDR from baseline at Weeks 4 and 12. Note: IneGDR = 4.964 − 0.121 * HbA1c (%) − 0.012 * diastolic blood pressure (mmHg) − 1.409 * WHR.

④Cellular immune modulation

Treg and Th17 proportions: changes from baseline at Weeks 4 and 12 in the proportions of peripheral blood CD4⁺CD25⁺FoxP3⁺ regulatory T cells (Tregs) and CD4⁺IL-17A⁺ T helper 17 cells (Th17);

Islet autoantibody titers: changes from baseline at Weeks 4 and 12 in titers of GADA, IAA, and ICA, as well as changes in the proportion of participants achieving a ≥50% reduction in autoantibody titers.

Infection and inflammation markers: Changes from baseline at Weeks 4 and 12 in white blood cell count (WBC), CRP, ESR, PCT, and inflammatory mediators including TNF-α, IL-1β, IL-8, IFN-γ, lipopolysaccharide antibodies, and zonulin antibodies.

⑤Gut microecological remodelingz:

Changes in key bacterial taxa: differences from baseline at Week 12 in the relative abundance of beneficial genera, such as Akkermansia and Roseburia, in fecal samples;

⑥Bile acid metabolic profile:

changes from baseline at Week 12 in secondary bile acids and SCFAs in blood and fecal samples;

Epitranscriptomic signatures of microbiota-derived cfRNA: changes from baseline at Week 12 in cfRNA methylation sites and patterns in blood samples. Statistical modeling will be applied to identify cfRNA methylation biomarkers associated with response to yFMT therapy.

⑦Patient-reported outcomes (PROs):

Fear of hypoglycemia: change from baseline at Weeks 4 and 12 in HFS-WSII scores;

Diabetes-related distress: change from baseline at Weeks 4 and 12 in DDS scores;

Quality of life: change from baseline at Weeks 4 and 12 in EQ-5D-5L index and visual analog scale scores.

⑧Lipid metabolism: Changes from baseline at Weeks 4 and 12 in TC, TG, LDL-c, and HDL-c.

⑨Insulin dosage: Change from baseline at Weeks 4 and 12 inTDD(U/day).

**8.2 Safety evaluation**

①Incidence of ADEs:Adverse events will be recorded and graded according to the Common Terminology Criteria for Adverse Events (CTCAE), version 5.0, including but not limited to gastrointestinal reactions (diarrhea, abdominal distension, vomiting), infection-related risks (e.g., events associated with microbial translocation), immune-related adverse events, and allergic reactions.

②Incidence of SAEs:Serious adverse events include severe hypoglycemic episodes, DKA, and other SAEs, defined as medical events occurring during the clinical trial that result in hospitalization or prolonged hospitalization, persistent or significant disability or incapacity, life-threatening conditions, death, or congenital anomalies.

## 9. Monitoring of Adverse Events

### 9.1 Assessment of adverse events

#### (1) Definitions

a. **AE:**Any unfavorable medical event occurring from the time a subject signs the informed consent form and is enrolled in the study until the last follow-up visit, regardless of whether it is causally related to FMT, shall be defined as an AE.

b. **Significant AE:**Any AE that necessitates targeted medical interventions (such as treatment discontinuation, dose reduction, or symptomatic management), including hematological and/or other laboratory abnormalities.

#### (2) Grading criteria for the intensity of adverse events

All clinical AEs occurring during this study will be recorded in the adverse event section of the study case report form (CRF), and the intensity of each AE will be graded. To ensure consistency, AE intensity will be classified as follows:

a. **Mild:** Perceptible discomfort that does not interfere with daily activities;

b. **Moderate:** Discomfort of sufficient severity to interfere with or reduce daily activities;

c. **Severe:** Inability to work or perform normal daily activities.

It is important to distinguish **severity** from **intensity** of an AE. The term severe refers to intensity and does not necessarily indicate a SAE. For example, a headache may be severe in intensity but does not qualify as an SAE unless it meets the criteria for an SAE.

#### (3) Criteria for assessing the relationship between adverse events and FMT

Causality between all AEs and FMT will be assessed using a five-level classification: **definitely related, probably related, possibly related, possibly unrelated, and definitely unrelated**. The first three categories will be classified as adverse reactions related to the investigational intervention.

Causality assessment will be based on the following five criteria:

a. A reasonable temporal relationship with FMT (i.e., occurrence within a defined post-transplantation time window);

b. The type of event is a known or theoretically plausible reaction following FMT, as supported by existing literature;

c. The severity or duration of the event is consistent with the dose or route of FMT administration;

d. The event cannot be reasonably explained by the subject’s underlying clinical condition, comorbidities, or other concomitant treatments or dietary changes;

e. Objective evidence (e.g., microbiota profiling, inflammatory markers) supports an association with FMT.

Investigators should evaluate the potential relationship between AEs, FMT, and concomitant medications, with reference to the table below.

| ****Assessment criteria**** | ****Assessment results**** | | | | |
| --- | --- | --- | --- | --- | --- |
|  | ****1**** | ****2**** | ****3**** | ****4**** | ****5**** |
| ****Definitely related**** | ****+**** | ****+**** | ****-**** | ****+**** | ****+**** |
| ****Probably related**** | ****+**** | ****+**** | ****-**** | ****+**** | ****?**** |
| ****Possibly related**** | ****+**** | ****+**** | ****±**** | ****±**** | ****?**** |
| ****Possibly unrelated**** | ****+**** | ****-**** | ****±**** | ****±**** | ****?**** |
| ****Definitely unrelated**** | ****-**** | ****-**** | ****+**** | ****-**** | ****-**** |

Notes: “+” indicates definitely present; “-” indicates absent; “±” indicates difficult to confirm or exclude; “?” indicates unknown.

Based on the above table, the relationship between adverse events and the investigational product will be classified into the following five categories:

1: Definitely related, 2: Probably related, 3: Possibly related, 4: Possibly unrelated,

5: Definitely unrelated;

The numerator will be the total number of subjects classified as categories 1 + 2 + 3, and the denominator will be the total number of enrolled subjects evaluable for AE assessment.

**9.2 Assessment of SAE**

Definition: A serious adverse event (SAE) refers to any clinical event associated with the investigational product at any dose or occurring at any time during the observation period that suggests a significant hazard, contraindication, adverse reaction, or condition requiring special caution. An AE meeting one or more of the following criteria will be classified as an SAE:

a. Death;

b. Life-threatening events (defined as situations in which the subject was at immediate risk of death at the time of the event; this does not include events that might have resulted in death if they had been more severe);

c. Hospitalization or prolongation of existing hospitalization;

d. Persistent or significant disability or loss of working capacity;

e. Congenital anomaly or birth defect.

Certain medical events that do not result in death, are not life-threatening, or do not require hospitalization may nevertheless be considered SAEs if, based on appropriate medical judgment, they may jeopardize the subject or require medical or surgical intervention to prevent one of the outcomes listed above.

**9.3 SAE Management**

When an AE occurs, the investigator may determine appropriate management measures based on the subject’s clinical condition. Generally, the following approaches may be adopted:

① Observation without discontinuation of FMT;

② Observation with discontinuation of FMT, without rescue therapy;

③ Discontinuation of FMT with initiation of rescue therapy.

All AEs shall be followed up and thoroughly documented, including management procedures and outcomes, until the event is appropriately resolved or the subject’s condition stabilizes. Laboratory abnormalities shall be followed until they return to normal or to pre-FMT baseline levels. Follow-up methods may include hospitalization, outpatient visits, home visits, telephone calls, or other communication methods, depending on the severity of the AE. If widespread unexpected adverse reactions occur during the clinical study, the drug regulatory authority and the ethics committee may decide, as appropriate, to suspend or terminate the clinical trial, and such decisions shall be documented.

**9.4 Risk assessment and AE management**

The investigational intervention in this study may be associated with adverse reactions such as nausea, abdominal pain, diarrhea, abdominal distension, fatigue, constipation, and decreased appetite. Potential risk events include DKA, severe hypoglycemia, and severe allergic reactions (e.g., dyspnea or even anaphylactic shock). If any of the above adverse reactions or risk events occur, physicians will promptly provide free symptomatic treatment. If necessary, the intervention will be discontinued, and appropriate compensation will be provided to the subject.

**9.5 Follow-up and documentation of AE**

All AEs, particularly those related to the investigational product, shall be followed until they return to baseline status or become stable. If recovery to baseline or stabilization cannot be achieved after follow-up, this shall be documented and explained in the study records. Except for SAEs that are not required to be reported immediately as specified in the protocol or other study documents (e.g., Investigator’s Brochure), investigators shall submit written reports of all SAEs, followed by timely and detailed written follow-up reports. SAE reports and follow-up reports shall identify subjects by their clinical trial identification codes rather than by their real names, national identification numbers, or residential addresses. AEs and laboratory abnormalities deemed important for safety evaluation as specified in the protocol shall be reported within 24 hours. In the case of death, investigators shall submit autopsy reports and final medical reports to the ethics committee.

**9.6 Abnormal laboratory findings**

Investigators shall assess whether abnormal laboratory results are clinically significant and provide possible explanations. Laboratory abnormalities resulting from reported AEs shall also be recorded as AEs in the AE case report form. Clinically significant laboratory abnormalities meeting one or more of the following criteria shall be recorded as independent diagnoses in the AE section of the study records (excluding laboratory abnormalities already reported as consequences of AEs):

a. Accompanied by clinical symptoms;

b. Resulting in modification of the investigational treatment;

c. Requiring changes in concomitant medications and/or other therapeutic interventions.

**10. Quality control and quality assurance of the study**

**10.1 Study process management**

(1) Establishment of an independent quality control team

An independent quality control team composed of physicians not involved in this trial will be established to oversee all aspects of the study, ensuring its objectivity and scientific integrity. The main responsibilities include:

① Monitoring the implementation of all trial procedures across each study phase;

② Monitoring adherence to intestinal microbiota extract capsule intake during the intervention period;

③ Conducting weekly verification of CGM wear compliance, dietary records, and concomitant medication use;

④ Randomly auditing original source documents for 20% of enrolled cases (e.g., insulin dose adjustment logs).

(2) Quality control of intestinal microbiota extract capsules:

① Donor screening: The manufacturer shall provide donor health certificates, microbiota profiling reports (e.g., Akkermansia abundance), and pathogen screening results (all negative).

② Capsule preparation: Each batch of capsules shall be accompanied by viable bacterial count reports and microbiota stability testing results.

③ Transportation and storage: Capsules shall be transported under −80 °C or liquid nitrogen conditions and stored in designated ultra-low-temperature freezers at the study site, with continuous temperature monitoring. Complete records of receipt, storage, and use shall be maintained.

(3) Standardization of sample collection and testing

① Blood samples: Two tubes of 5 mL anticoagulated whole blood will be collected using vacuum tubes; serum separation will be completed within 1 hour; aliquots will be stored at −80 °C.

② Fecal samples: Samples will be preserved using DNA/RNA stabilizing agents to prevent microbial degradation.

③ Testing institutions: Laboratories must hold ISO 9001 quality management system certification and provide documentation of standardized operating procedures.

**10.2 Quality control measures**

(1) Qualification review of investigators

Investigators participating in the clinical trial must possess appropriate expertise, qualifications, and capabilities in clinical research. Investigator eligibility will be confirmed through qualification review, and personnel assignments will remain relatively stable.

(2) Pre-trial training

① Training content: Comprehensive understanding of the study protocol and all outcome measures; standardized operating procedures; adverse event monitoring and management.

② Key requirements: Objective documentation of symptoms; conducting examinations according to specified schedules and methods; complete and accurate completion of study medical records.

(3) Investigational product management

Medication adherence will be monitored using pill count methods; follow-up management will be strengthened; complete records of investigational product use will be maintained.

(4) AE management

Potential adverse reactions will be communicated in advance; emergency response plans will be established; a rapid reporting mechanism will be implemented.

**10.3 Management of participant compliance**

(1) Inpatient period management

Participants will remain hospitalized throughout the intervention period. Dedicated staff will be responsible for drug dispensing and supervision of administration. Daily records will be maintained documenting intake of yFMT capsules or placebo capsules and any adverse events.

(2) Follow-up period management

Regular follow-up visits will be conducted; communication and education will be strengthened to improve follow-up completion rates.

(3) Monitoring system

Independent monitors appointed by the sponsor will conduct periodic reviews of study documentation to ensure compliance with GCP requirements.

**11. Data safety monitoring**

A data safety monitoring plan will be developed based on the level of study risk. All adverse events will be thoroughly documented, appropriately managed, and followed until resolution or stabilization, and serious and unexpected adverse events will be reported in a timely manner to the ethics committee, competent authorities, and drug regulatory agencies as required. The principal investigator will periodically conduct cumulative reviews of all adverse events. If necessary, investigator meetings will be convened to evaluate the risk–benefit profile of the study. For studies exceeding minimal risk, independent data monitors will be appointed to review study data. For high-risk studies, an independent Data and Safety Monitoring Board will be established to review accumulated safety and efficacy data and to provide recommendations on study continuation.

**12. Statistical analysis**

All efficacy endpoints will be analyzed based on the intention-to-treat (ITT) population. Participants who do not complete end-of-treatment assessments will remain included in the analysis and will be classified as non-responders. Safety endpoints will be analyzed in the safety analysis set. Key CGM metrics recommended by international expert consensus were calculated using Glyculator 2.0 software (Medical University of Lodz, Lodz, Poland).

For continuous variables, normality will be assessed using the Shapiro–Wilk test. Normally distributed data will be expressed as mean ± standard deviation and analyzed using independent-sample t tests (between-group comparisons) or paired t tests (within-group comparisons). Non-normally distributed data will be expressed as median (Q1, Q3) and analyzed using the Mann–Whitney U test (between groups) or the Wilcoxon signed-rank test (within groups). Categorical variables will be presented as counts (%) and compared using Fisher’s exact test. The primary efficacy endpoint (composite response rate) and categorical secondary endpoints will be analyzed using generalized estimating equations to assess group effects, time effects, and interaction effects. Continuous secondary endpoints (e.g., changes in HbA1c and C-peptide levels) will be analyzed using linear mixed-effects models, with fixed effects including group, time, interaction terms, and baseline values, and random effects specified at the subject level. Safety outcomes (incidence of adverse events) will be compared using Fisher’s exact test. Logistic regression analysis will be performed to explore factors associated with response to yFMT treatment. Missing data for the primary endpoint will be handled using the last observation carried forward method.

All analyses will be conducted using R software (version 4.3.2) and GraphPad Prism 9.0. All statistical tests will be two-sided, with P < 0.05 considered statistically significant. Bonferroni correction will be applied for post-hoc multiple comparisons.

**13. Medical Ethics**
This study will be conducted in accordance with the Declaration of Helsinki and other relevant international and national regulations. The study will not commence until the protocol has been reviewed and approved by the ethics committee. Prior to enrollment, investigators are responsible for providing subjects or their legal representatives with a complete and comprehensive explanation of the study’s purpose, procedures, and potential risks. Written informed consent must be obtained before participation. Subjects will be informed of their right to withdraw from the study at any time. Signed informed consent forms will be retained as part of the clinical study documentation. Throughout the study, the privacy of participants and the confidentiality of their personal data will be strictly protected.

14. **Study timeline**

Refinement of the study protocol: July–August 2025

Research project approval: September 2025

Ethics approval: September 2025

Participant recruitment and intervention: October 2025–July 2026

Database lock: August 2026

Data analysis and manuscript preparation: September–December 2026

15. **Study personnel**

| **Name** | **Title / Specialty** | **Responsibilities** | **GCP Training (Date)** |
| --- | --- | --- | --- |
| Zhe Dai | Chief Physician / Endocrinology | Study design, quality control and supervision | December 2024 |
| Mengyun Lei | Attending Physician / Endocrinology | Study design, study implementation, data analysis, manuscript preparation | December 2024 |
| Jun Tang | Associate Chief Physician / Endocrinology | Study implementation, data collection, manuscript preparation | December 2024 |
| Shuting Li | Senior Nurse / Endocrinology | Follow-up and data collection | December 2024 |
| Fang Liu | Senior Nurse / Endocrinology | Follow-up and data collection | December 2024 |

**16.References**

1. Alberti KG, Zimmet PZ. Definition, diagnosis and classification of diabetes mellitus and its complications. Part 1: diagnosis and classification of diabetes mellitus provisional report of a WHO consultation. Diabet Med. 1998;15(7):539-553.

2. Yang Z, Wang K, Li T, et al. Childhood diabetes in China. Enormous variation by place and ethnic group. Diabetes Care. 1998;21(4):525-529.

3. Weng J, Zhou Z, Guo L, et al. Incidence of type 1 diabetes in China, 2010-13: population based study. BMJ. 2018;360:j5295. Published 2018 Jan 3.

4. Chiang JL, Kirkman MS, Laffel LM, Peters AL; Type 1 Diabetes Sourcebook Authors. Type 1 diabetes through the life span: a position statement of the American Diabetes Association. Diabetes Care. 2014;37(7):2034-2054.

5. Beck RW, Tamborlane WV, Bergenstal RM, et al. The T1D Exchange clinic registry. J Clin Endocrinol Metab. 2012;97(12):4383-4389.

6. iller KM, Foster NC, Beck RW, et al. Current state of type 1 diabetes treatment in the U.S.: updated data from the T1D Exchange clinic registry. Diabetes Care. 2015;38(6):971-978.

7. Li J, Yang D, Yan J, et al. Secondary diabetic ketoacidosis and severe hypoglycaemia in patients with established type 1 diabetes mellitus in China: a multicentre registration study. Diabetes Metab Res Rev. 2014;30(6):497-504.

8. Liu L, Yang D, Zhang Y, et al. Glycaemic control and its associated factors in Chinese adults with type 1 diabetes mellitus. Diabetes Metab Res Rev. 2015;31(8):803-810.

9. Qin ZY, Yan JH, Yang DZ, et al. Behavioral Analysis of Chinese Adult Patients with Type 1 Diabetes on Self-monitoring of Blood Glucose. Chin Med J (Engl). 2017;130(1):39-44.

10.Zheng XY, Weng JP, Luo SH, Xiong SS, Yang DZ, Qiu LL, et al. [A cross-sectional investigation of self-monitoring of blood glucose in adults with type 1 diabetes melitus in Guangdong Province]. Chin J Diabetes. 2016;8(4):215–19. Chinese.

11.Knip M, Siljander H. The role of the intestinal microbiota in type 1 diabetes mellitus. Nat Rev Endocrinol. 2016;12(3):154-167.

12.de Goffau MC, Fuentes S, van den Bogert B, et al. Aberrant gut microbiota

composition at the onset of type 1 diabetes in young children. Diabetologia. 2014;57(8):1569-1577.

13.Burger-van Paassen N, Vincent A, Puiman PJ, et al. The regulation of intestinal

mucin MUC2 expression by short-chain fatty acids: implications for epithelial

protection. Biochem J. 2009;420(2):211-219. Published 2009 May 13.

14.Calcinaro F, Dionisi S, Marinaro M, et al. Oral probiotic administration induces

interleukin-10 production and prevents spontaneous autoimmune diabetes in the

non-obese diabetic mouse. Diabetologia. 2005;48(8):1565-1575.

14. Mithieux G. Does Akkermansia muciniphila play a role in type 1 diabetes?. Gut. 2018;67(8):1373-1374.

16.Allegretti JR, Mullish BH, Kelly C, Fischer M. The evolution of the use of faecal

microbiota transplantation and emerging therapeutic indications. Lancet. 2019;394(10196):420-431.

17.Allegretti JR, Kassam Z, Mullish BH, et al. Effects of Fecal Microbiota

Transplantation With Oral Capsules in Obese Patients. Clin Gastroenterol Hepatol. 2020;18(4):855-863.e2.

18.Fei N, Zhao L. An opportunistic pathogen isolated from the gut of an obese

human causes obesity in germfree mice. ISME J. 2013;7(4):880-884.

19.de Groot P, Nikolic T, Pellegrini S, et al. Faecal microbiota transplantation halts

progression of human new-onset type 1 diabetes in a randomised controlled trial. Gut. 2021;70(1):92-105.

20.Li YY, Zhu YX, Zhou Y, Qian C, Zhou J, Chen RR, et al. [Efficacy and safety of washed microbiota transplantation in the treatment of brittle diabetes]. Chin J Diabetes. 2020;12(12): 962–7. Chinese.

21.Liu Q, Hua Y, He R, et al. Restoration of intestinal secondary bile acid synthesis: A

potential approach to improve pancreatic β cell function in type 1 diabetes. Cell Rep Med. 2025;6(5):102130.

22. Høyer KL, Dahl Baunwall SM, Kornum DS, et al. Faecal microbiota transplantation for patients with diabetes type 1 and severe gastrointestinal neuropathy (FADIGAS): a randomised, double-blinded, placebo-controlled trial. EClinicalMedicine. 2024;79:103000. Published 2024 Dec 16.

23. Yang J, Yang X, Wu G, et al. Gut microbiota modulate distal symmetric polyneuropathy in patients with diabetes. Cell Metab. 2023;35(9):1548-1562.e7.
