# Supplementary material for: Efficacy, safety, and feasibility of youth-derived fecal microbiota transplantation among adults with type 1 diabetes mellitus: A protocol of Pilot Randomized Controlled Trial
Source: PLoS One. 2026 Sep 21;21(9):e0343078. doi: 10.1371/journal.pone.0343078 (PMC13592622; doi:10.1371/journal.pone.0343078)
Supplement: Supplementary file 7 [file pone.0343078.s006.doc]

The authors have addressed the major concerns, and the manuscript has been substantially improved. A minor issue remains and should be addressed:

1. **Please further clarify the strategy for handling missing data in the statistical analysis.** Authors state that participants missing the final assessment will be classified as non-responders, while multiple imputation is also described for handling missing data. It seems to be a paradox, the authors should clarify.
