# Supplementary material for: Efficacy, safety, and feasibility of youth-derived fecal microbiota transplantation among adults with type 1 diabetes mellitus: A protocol of Pilot Randomized Controlled Trial
Source: PLoS One. 2026 Sep 21;21(9):e0343078. doi: 10.1371/journal.pone.0343078 (PMC13592622; doi:10.1371/journal.pone.0343078)
Supplement: Supplementary file 4 [file pone.0343078.s007.docx]

Dear Dr. Ze Xiang,

Academic Editor, PLOS ONE,

Thank you very much for giving us the opportunity to revise our manuscript entitled “Efficacy, safety, and feasibility of Youth-derived Fecal Microbiota Transplantation among adults with Type 1 Diabetes Mellitus: A protocol of pilot randomized controlled trial” (PONE-D-26-05078).

We would also like to express our sincere gratitude to the reviewers for their constructive, thorough, and helpful comments. We have carefully addressed all requirements and reviewers’ comments in the revised manuscript. All changes in the manuscript have been highlighted using the “Track Changes” function in the Word file labeled ‘Revised Manuscript with Track Changes’.

The point-by-point responses to the Journal Requirements and Reviewers’ comments are detailed below.

**Part 1: Response to Journal Requirements**

To the Editor:

We have carefully reviewed the Journal Requirements and have ensured that the manuscript complies with all of them. Our specific responses are as follows:

**Requirement 1: Please ensure that your manuscript meets PLOS ONE's style requirements, including those for file naming.**

**-Response:** Thank you for the reminder. We have thoroughly reviewed the PLOS ONE style templates and carefully revised the manuscript’s formatting, layout, and file naming (including figures and tables) to ensure full compliance with the guidelines.

**Requirement 2: We note that you have indicated that there are restrictions to data sharing for this study. PLOS only allows data to be available upon request if there are legal or ethical restrictions on sharing data publicly.**

**-Response:** We thank you for raising this important issue regarding data sharing. We have carefully reviewed PLOS ONE's data availability policy and have addressed your concerns as follows:

As this manuscript is a study protocol, the clinical dataset has not yet been generated. Upon study completion, data will be available upon reasonable request, subject to corresponding author (Zhe Dai, daizhe@znhospital.cn).

We have revised in the ‘**Data Availability**’ section as follows:

“*As this manuscript is a study protocol, the clinical dataset has not yet been generated. Upon study completion, data will be available upon reasonable request, subject to corresponding author (Zhe Dai, daizhe@znhospital.cn).*” (Page 27, Line 535-537).

**Requirement 3: If the reviewer comments include a recommendation to cite specific previously published works, please review and evaluate these publications to determine whether they are relevant and should be cited. There is no requirement to cite these works unless the editor has indicated otherwise.**

**-Response:** We acknowledge the journal's policy regarding citation recommendations. We confirm that the reviewers did not suggest specific citations in this round of review. Furthermore, we have carefully scrutinized the reference list in the revised manuscript to ensure that all cited works are directly relevant to our study and contribute to the scientific context.

**Part 2: Response to reviewers**

===========================================================

RESPONSES TO REVIEWER #1

===========================================================

**Comment 1: Line 271. The primary endpoint was the composite achievement rate of TIR > 70% +TBR < 4%. Please clarify this definition with more specific definition.**

**-Response:** Thank you for this valuable comment. We have revised the manuscript to provide a precise definition of the primary endpoint.The primary endpoint is the composite achievement rate of TIR (time in range 3.9-10.0 mmol/L) >70% and TBR3.9 (time below range <3.9 mmol/L) <4% at 3 months (weeks 10-12) post-intervention, relative to baseline (days -17 to -3 during the run-in period).

We have corrected our expression in the ‘**Methods > Endpoints**’ section :

“*The primary endpoint is change in the composite achievement rate of TIR>70% and TBR3.9 <4% at 3 months (Weeks 10-12) post-intervention compared to baseline (days -17 to -3 during the run-in period).*” (Page 16, Line 311-313).

**Comment 2: Line 358. The pre-planned interim analysis will be conducted. Please clarify and be more specific on what interim analysis will be conducted and what action will be taken based on the pre-planned interim analysis.**

**-Response:** Thank you for this important methodological comment. We apologize for the lack of clarity in our original description.

We have revised the relevant section to specify that the pre-planned interim analysis will be conducted after the first 10 participants (50% of the total sample) have completed the 12-week follow-up, with the following explicit objectives:

(1) **Safety monitoring:** The independent Data Monitoring Committee (DMC) will review all unblinded safety data (adverse events, severe adverse events, and hypoglycemic episodes) to determine whether the trial should continue, be modified, or be terminated prematurely due to unacceptable toxicity or safety signals.
(2) **Effect size estimation for future trials:** The DMC will also review the unblinded primary endpoint data (composite achievement rate of TIR >70% with TBR3.9 <4%) to provide a preliminary estimate of the treatment effect (with 95% confidence intervals). This estimate will not be used to modify the sample size of the current trial, given its pilot nature (n=20), but will serve as the basis for sample size calculations in a future confirmatory randomized controlled trial.
(3) **Futility assessment (exploratory):** If the observed treatment effect in the intervention group shows no directionally favorable trend toward the composite endpoint compared with the placebo group, the DMC may recommend early termination to avoid exposing additional participants to an ineffective intervention.

Importantly, no formal hypothesis testing will be performed at the interim analysis, and the type I error rate will not be consumed, as the current trial is designed as a pilot study rather than a confirmatory trial. The final analysis will remain definitive.

We have revised in the ‘**Methods > Sample Size and Pilot Trial Rationale**’ section as follows:

“*A pre-planned interim analysis will be conducted after the first 10 participants (50% of the total planned sample) have completed the 12-week follow-up. An independent Data Monitoring Committee (DMC) will review unblinded safety data to monitor for unacceptable AEs and will evaluate the primary composite endpoint to estimate the treatment effect for future sample size planning. No formal hypothesis testing will be performed, and no sample size re-estimation is planned for the current trial, consistent with its pilot nature. Based on the DMC’s review, the trial may continue as planned, be modified, or be terminated early if unacceptable safety concerns or clear futility are observed.*” (Page 21, Line 415-423)

**Comment 3: Line 373. To increase the transparency, please clarify the specifications for GEE, such as the correlation structure and so on.**

**-Response:** Thank you for this important methodological comment. We agree that greater transparency regarding the correlation structure is necessary.

We have revised our statistical analysis plan as follows:

For the primary efficacy endpoint—the composite glycemic target achievement rate (TIR >70% with TBR3.9<4%) measured at two post-baseline time points (Week 4 and Week 12) —we will employ a generalized estimating equation (GEE) model with a binary distribution, logit link function, and an exchangeable correlation structure, which assumes equal correlation between repeated measurements within the same participant. The model will include group (yFMT vs. placebo), time (Week 4 vs. Week 12), and the group-by-time interaction as fixed effects, with the corresponding baseline value as a covariate when applicable. The primary inferential focus will be on the group effect across both time points, while the interaction term will be used to assess whether the treatment effect varies over time.

Given the small sample size (n=20), we will also perform Fisher’s exact test as a sensitivity analysis at each individual time point to confirm the robustness of the findings. The results of both analyses will be reported.

We have revised in the ‘**Methods > Statistics and Analysis**’ section:

“*For the primary efficacy endpoint assessed at Week 4 and Week 12 post-intervention, a generalized estimating equation (GEE) model with a binary distribution, logit link function, and an exchangeable correlation structure will be employed. The model includes group, time, and their interaction as fixed effects, with baseline composite achievement status as covariates as appropriate. The group effect averaged across both time points will serve as the primary inferential basis. Given the pilot nature of the study (n=20), Fisher’s exact test will additionally be performed at each time point as a sensitivity analysis to ensure robustness.*” (Page 22, Line 436-443)

**Comment 4: Line 376. It is unclear what covariates will be adjusted in the model. Please clarify this.**

**-Response:** Thank you for this comment. We have revised the statistical analysis plan to explicitly define the covariates to be adjusted in the linear mixed-effects models.

For the primary efficacy analysis—the composite glycemic target achievement rate (TIR >70% with TBR3.9 <4%) analyzed using the GEE model—no covariates other than the baseline composite achievement status will be adjusted, given the small sample size (n=20) and the pilot nature of the study.

Group, time, and their interaction are structural fixed effects of the repeated-measures design, rather than additional covariates. The covariates adjusted in the LMM are listed below.

| **Covariate** | **Rationale** |
| --- | --- |
| Baseline value of the respective outcome | To adjust for regression to the mean and increase statistical precision |
| Age | Known to influence glycemic control and immune function in T1DM |
| T1DM duration | Longer duration is associated with lower residual β-cell function and may affect C-peptide outcomes |

Importantly, to avoid model overfitting given the limited sample size (n=20), we adhere to the rule of thumb of ≤1 covariate per 5-10 participants for the primary model. The full covariate-adjusted model will be used for the primary analysis, while a parsimonious model adjusting only for baseline value, group, and time will be performed as a sensitivity analysis to confirm robustness.

We have revised the manuscript in the ‘**Methods > Statistics and Analysis**’ section as follows:

“*For continuous secondary outcomes, linear mixed-effects models will be applied with group, time, and their interaction as fixed effects, the baseline value of the respective outcome as a covariate, and age and T1DM duration as additional covariates, with a random intercept for each participant. Multicollinearity will be assessed using the variance inflation factor (VIF), with VIF >10 indicating significant collinearity. Given the limited sample size, a parsimonious sensitivity model including only baseline value, group, and time will also be performed to assess robustness against overfitting.*” (Page 22,23, Line 443-450)

**Comment 5: Line 378. The missing data for the primary endpoint will be imputed with the last observation. This is concerning to impute the primary endpoint!**

**-Response:** Thank you for raising this important methodological concern. We fully acknowledge that the last observation carried forward (LOCF) method has inherent limitations, particularly the assumption that participants’ outcomes remain unchanged after dropout, which may introduce bias.

We have adopted multiple imputation to handle missing values. Specifically, 20 imputed datasets will be generated, with the imputation model including baseline outcome values, age, T1DM duration, and treatment group as auxiliary variables. Results will be pooled across imputed datasets using Rubin’s rules.

We have revised the manuscript in the ‘**Methods > Statistics and Analysis**’ section as follows:

“*Missing data will be handled using multiple imputation, generating 20 imputed datasets with imputation models including baseline outcome values, age, T1DM duration, and treatment group. Results will be pooled using Rubin’s rules.*” (Page 23, Line 458-461)

===========================================================

RESPONSES TO REVIEWER #2

===========================================================

**Major revisions:**

**Comment 1: Scientific Rationale and Novelty: The concept of youth-derived FMT (yFMT) is innovative and relevant. However, the rationale for selecting young donors (4–17 years) requires stronger justification. The claim that this age group represents “peak microbiota diversity” is not sufficiently supported by direct evidence.**

**-Response:** We thank the reviewer for this critical comment. We agree that the original claim of “peak microbiota diversity” lacked sufficient direct evidence, and we have removed this statement.

Our selection of donors aged 4-17 years is now supported by evidence from two complementary lines:

1. First, longitudinal studies have demonstrated that the gut microbiota during childhood and adolescence exhibits greater temporal stability compared to both early infancy and older age groups [1,2]. This stability is theoretically advantageous for successful engraftment, as strains stably maintained in the donor are more likely to persist in the recipient [3].
2. Second, animal studies consistently show that young donor microbiota confers metabolic and immune benefits when transplanted into aged or diseased recipients. For instance, young-to-old FMT in mice reduced body weight, prevented fat accumulation, improved frailty, and reversed age-related inflammation in the brain and retina [4,5]. In sex-matched mouse models, early-life FMT from children induced long-lasting effects on adipose tissue function and systemic glucose homeostasis that persisted into adulthood [6].

While direct evidence linking young donor age to superior outcomes specifically in T1DM remains to be established—which is precisely what our pilot study aims to generate—the convergence of ecological rationale and animal data provides a strong justification for our donor selection strategy.

We have revised the manuscript accordingly in the ‘**INTRODUCTION**’ section:

“*The yFMT capsules are prepared from rigorously screened healthy young donors (aged 4-17 years), selected for the relatively high temporal stability of the gut microbiota during this developmental window.*”(Page 5, Line 101-103)

We also have revised the manuscript accordingly in the ‘**Methods>Study intervention**’ section:

“*The yFMT capsules are manufactured by Meiyitian Biomedical Co., Ltd. (Wuhan, Hubei, China) and prepared from rigorously screened healthy young donors (aged 4-17 years), selected for the relatively high temporal stability of the gut microbiota during this developmental window*.”(Page 11, Line 216-219)

We also have revised the manuscript accordingly in the ‘**DISCUSSION**’ section:

“*The rationale for selecting young donors (aged 4-17 years) is grounded in the relatively high gut microbiota temporal stability characteristic of this developmental window*.”(Page 24, Line 486-488).

**REFERENCES:**

[1] Yatsunenko T, Rey FE, Manary MJ, et al. Human gut microbiome viewed across age and geography. *Nature*. 2012;486(7402):222-227.

[2] Wu J, Shen H, Lv Y, et al. Age over sex: evaluating gut microbiota differences in healthy Chinese populations. *Front Microbiol*. 2024;15:1412991.

[3] Schmidt TSB, Li SS, Maistrenko OM, et al. Drivers and determinants of strain dynamics following fecal microbiota transplantation. Nat Med.2022;28(9):1902-1912.

[4] Parker A, Romano S, Ansorge R, et al. Fecal microbiota transfer between young and aged mice reverses hallmarks of the aging gut, eye, and brain. *Microbiome*. 2022;10(1):68.

[5] Xie J, Kim T, Liu Z, et al. Young gut microbiota transplantation improves the metabolic health of old mice. *mSystems*. 2025;10(6):e0160124.

[6] La Rosa F, Guzzardi MA, Pardo-Tendero M, et al. Effects of children’s microbiota on adipose and intestinal development in sex-matched mice persist into adulthood following a single fecal microbiota transplantation. *Mol Metab*. 2025;97:102157.

**Comment 2: Study Design and Sample Size: The study is appropriately designed as a randomized controlled pilot trial. The sample size (n=10 per group) is justified based on pilot study guidance . However: The small sample limits interpretability of efficacy outcomes. The manuscript should clearly emphasize feasibility objectives (e.g., recruitment, adherence).**

**-Response:** We thank the reviewer for this important comment. We fully agree that the small sample size (n=10 per group) limits the interpretability of definitive efficacy outcomes. In response, we have reframed the study objectives throughout the manuscript to clearly emphasize its pilot/feasibility nature.

Specifically, we have revised the following sections:

We have revised **the title of the manuscript,** we now state:

“*Efficacy, safety, and feasibility of Youth-derived Fecal Microbiota Transplantation among adults with Type 1 Diabetes Mellitus: A protocol of pilot randomized controlled trial.*”(Page1, Line 1-4)

In the ‘**ABSTRACT**’ section, we now state:

“*This pilot trial assessed the feasibility, safety, and preliminary efficacy of yFMT in adults with T1DM.*”(Page 2, Line 26,27)

“*Feasibility will be assessed through recruitment rate, retention rate, intervention adherence, and acceptability.*”(Page 3, Line 44,45)

In the ‘**INTRODUCTION**’ section, we now state:

“*our study pioneers the oral use of yFMT in a pilot RCT to evaluate the feasibility, safety, and preliminary efficacy of yFMT in adults with T1DM.*”(Page5, Line 103-105)

In the ‘**Methods>Study Design**’ section, we now state:

“*This pilot, randomized, double-blind, placebo-controlled trial conducted at Zhongnan Hospital of Wuhan University (China), is designed primarily to assess the feasibility and safety of yFMT in adults with T1DM.*”(Page 6, Line 108-109)

In the ‘**Methods>Endpoints**’ section, we now state:

“*Feasibility endpoints include: (1) recruitment rate (proportion of screened participants enrolled); (2) retention rate (proportion of participants completing the 12-week follow-up); (3) intervention adherence (percentage of prescribed capsules consumed); and (4) acceptability (participant-reported tolerability and willingness to undergo treatment).*”(Page 17, Line 317-321)

In the ‘**Methods>Statistics and Analysis**’ section, we now state:

“*Feasibility endpoints will be analyzed using descriptive statistics. Recruitment rate will be calculated as the proportion of enrolled participants among all screened individuals. Retention rate will be calculated as the proportion of enrolled participants who complete the 12-week follow-up. Adherence to the intervention will be calculated as the proportion of prescribed capsules consumed. Acceptability will be summarized by the proportion of participants reporting tolerability and willingness to undergo the treatment. All Feasibility endpoints will be presented as frequencies and percentages, with 95% confidence intervals where appropriate.*”(Page 23, Line 450-458)

In the ‘**DISCUSSION**’ section, we now explicitly state:

“*This pilot study incorporates predefined Feasibility endpoints—including recruitment rate, retention rate, intervention adherence, and acceptability—which are critical for informing the design and conduct of a future confirmatory trial. Recruitment and retention rates will provide essential information on patient interest and the practicality of the study procedures. Adherence and acceptability data will help refine the intervention protocol and identify potential barriers to implementation in larger, multicenter settings. Together, these feasibility metrics, combined with the preliminary efficacy and safety data, will provide a comprehensive foundation for planning a subsequent adequately powered trial.*” (Page 25, Line 493-501)

We believe these revisions better position our study within its intended pilot framework and address the reviewer’s concern regarding efficacy interpretability.

**Comment 3: Primary Endpoint: The composite endpoint (TIR >70% and TBR <4%) is clinically meaningful. Clarification is needed on: Whether both criteria must be achieved simultaneously. Statistical handling of the composite endpoint.**

**-Response:** We thank the reviewer for this important methodological comment. We have revised the definition and statistical handling of the primary endpoint to ensure clarity.

**Regarding the definition:** The composite endpoint requires both criteria to be achieved simultaneously in the same individual—that is, TIR (3.9-10.0mmol/L) >70% AND TBR (<3.9mmol/L) <4% during the 14-day CGM assessment period. We have revised the ‘**Methods > Endpoints**’ section to clarify this point:

“*The primary endpoint is change in the composite achievement rate of TIR>70% and TBR3.9 <4% at 3 months (weeks 10-12) post-intervention compared to baseline (days -17 to -3 during the run-in period).*” (Page 15, Line 290-292)

**Regarding the statistical handling:** As the endpoint is binary (achievement vs. non-achievement) and measured at two post-baseline time points (Week 4 and Week 12), we will employ a generalized estimating equation (GEE) model with a binary distribution, logit link function, and exchangeable correlation structure to compare the composite achievement rates between groups. The model includes group, time, and their interaction as fixed effects, with baseline composite achievement status as a covariate. The group effect averaged across both time points will serve as the primary inferential basis. This approach appropriately accounts for the within-subject correlation between repeated measurements.

Given the pilot nature of the study (n=20), Fisher’s exact test will additionally be performed at each individual time point as a sensitivity analysis to confirm the robustness of the findings.

We have updated the ‘**Methods > Statistical Analysis**’ section accordingly:

“*For the primary efficacy endpoint assessed at Week 4 and Week 12 post-intervention, a generalized estimating equation (GEE) model with a binary distribution, logit link function, and an exchangeable correlation structure will be employed. The model includes group, time, and their interaction as fixed effects, with baseline composite achievement status as covariates as appropriate. The group effect averaged across both time points will serve as the primary inferential basis. Given the pilot nature of the study (n=20), Fisher’s exact test will additionally be performed at each time point as a sensitivity analysis to ensure robustness.*” (Page 22, Line 436-443)

**Comment 4: Statistical Analysis:**

**Did the author check the normality, which test has been used?**

**-Response:** We appreciate the reviewer’s question regarding our statistical methodology. Yes, normality of all continuous data was performed using the Shapiro-Wilk test at each time point prior to analysis. This is standard practice in our analytical pipeline.

Based on the test results, normally distributed data will be analyzed using independent samples t-test (between-group comparisons) or paired t-test (within-group comparisons), and presented as mean±standard deviation. Non-normally distributed data will be analyzed using Mann-Whitney U test (between-group) or Wilcoxon signed-rank test (within-group), and presented as median with interquartile range (Q1, Q3). This approach ensures appropriate statistical handling regardless of the underlying data distribution.

We have revised the ‘**Methods > Statistical Analysis**’ section accordingly:

“*Normality of continuous variables will be assessed using the Shapiro-Wilk test at each time point. Normally distributed data will be presented as mean±standard deviation and analyzed using independent samples t-test (between-group) or paired t-test (within-group). Non-normally distributed data will be presented as median (Q1, Q3) and analyzed using Mann-Whitney U test (between-group) or Wilcoxon signed-rank test (within-group).*” (Page 22, Line 428-433)

**The author also should mention the Statistical tests used and the level of significance in the Statistical Analysis and elaborate the abbreviated name below each table.**

**-Response:** We thank the reviewer for this comment. We have revised the statistical analysis section to clearly specify the tests used and the significance level, and we have ensured that all abbreviations are defined in table footnotes.

**Regarding the statistical tests:** For the primary composite endpoint (binary outcome, assessed at Week 4 and Week 12), we will employ a generalized estimating equation (GEE) model with a binary distribution, logit link, and exchangeable correlation structure, including group, time, and their interaction as fixed effects, with baseline values as covariates as appropriate. Fisher’s exact test will be used as a sensitivity analysis at each individual time point. For continuous secondary outcomes, linear mixed-effects models will be applied with group, time, and their interaction as fixed effects, the baseline value of the respective outcome as a covariate, and age and T1DM duration as additional covariates, with a random intercept for each participant. Multicollinearity will be assessed using the variance inflation factor (VIF), with VIF >10 indicating significant collinearity. Given the limited sample size, a parsimonious sensitivity model including only baseline value, group, and time will also be performed to assess robustness against overfitting. Feasibility endpoints will be analyzed using descriptive statistics. Recruitment rate will be calculated as the proportion of enrolled participants among all screened individuals. Retention rate will be calculated as the proportion of enrolled participants who complete the 12-week follow-up. Adherence to the intervention will be calculated as the proportion of prescribed capsules consumed. Acceptability will be summarized by the proportion of participants reporting tolerability and willingness to undergo the treatment. All Feasibility endpoints will be presented as frequencies and percentages, with 95% confidence intervals where appropriate. Statistical significance is defined as two-sided P < 0.05.We also have added a dedicated footnote below each table to fully define all abbreviations used.

We have revised the ‘**Methods > Statistical Analysis**’ section accordingly:

“*For the primary efficacy endpoint assessed at Week 4 and Week 12 post-intervention, a generalized estimating equation (GEE) model with a binary distribution, logit link function, and an exchangeable correlation structure will be employed. The model includes group, time, and their interaction as fixed effects, with baseline composite achievement status as covariates as appropriate. The group effect averaged across both time points will serve as the primary inferential basis. Given the pilot nature of the study (n=20), Fisher’s exact test will additionally be performed at each time point as a sensitivity analysis to ensure robustness. For continuous secondary outcomes, linear mixed-effects models will be applied with group, time, and their interaction as fixed effects, the baseline value of the respective outcome as a covariate, and age and T1DM duration as additional covariates, with a random intercept for each participant. Multicollinearity will be assessed using the variance inflation factor (VIF), with VIF >10 indicating significant collinearity. Given the limited sample size, a parsimonious sensitivity model including only baseline value, group, and time will also be performed to assess robustness against overfitting. Feasibility endpoints will be analyzed using descriptive statistics. Recruitment rate will be calculated as the proportion of enrolled participants among all screened individuals. Retention rate will be calculated as the proportion of enrolled participants who complete the 12-week follow-up. Adherence to the intervention will be calculated as the proportion of prescribed capsules consumed. Acceptability will be summarized by the proportion of participants reporting tolerability and willingness to undergo the treatment. All Feasibility endpoints will be presented as frequencies and percentages, with 95% confidence intervals where appropriate. Missing data will be handled using multiple imputation, generating 20 imputed datasets with imputation models including baseline outcome values, age, T1DM duration, and treatment group. Results will be pooled using Rubin’s rules. Logistic regression analysis will be used to explore factors associated with response to yFMT treatment. CGM metrics, as recommended by international expert consensus, will be calculated using Glyculator 2.0 software (Medical University of Lodz, Poland). All statistical analyses will be performed using R software (version 4.3.2) and GraphPad Prism 9.0. Statistical significance is defined as two-sided P<0.05.*” (Page 22,23, Line 436-466)

**The authors must explain how they controlled confounding factors, as it seems that adjustment of confounders is not considered. Did they assess multicollinearity?**

**-Response:** We thank the reviewer for this important methodological question. We have addressed both points as follows:

**Regarding confounding control:** For continuous secondary outcomes analyzed using linear mixed-effects models, we included the baseline value of the respective outcome as a covariate, and adjusted for the two most clinically relevant factors for T1DM outcomes (age and T1DM duration) as additional covariates. Group, time, and their interaction are structural fixed effects of the repeated-measures design rather than additional covariates, and were included accordingly. Given the limited sample size (n=20), we deliberately restricted additional covariate adjustment to age and T1DM duration to avoid overfitting. Moreover, a parsimonious sensitivity model adjusting only for baseline value, group, and time will also be performed to assess robustness.

**Regarding multicollinearity:** We will assess multicollinearity among all covariates in the final model using the variance inflation factor (VIF). A VIF >10 will be considered indicative of significant multicollinearity.

We have revised the ‘**Methods > Statistics and Analysis**’ section accordingly:

“*For continuous secondary outcomes, linear mixed-effects models will be applied with group, time, and their interaction as fixed effects, the baseline value of the respective outcome as a covariate, and age and T1DM duration as additional covariates, with a random intercept for each participant. Multicollinearity will be assessed using the variance inflation factor (VIF), with VIF >10 indicating significant collinearity. Given the limited sample size, a parsimonious sensitivity model including only baseline value, group, and time will also be performed to assess robustness against overfitting.*” (Page 22,23, Line 443-450)

**The proposed use of mixed-effects models and generalized estimating equations is appropriate. However: The use of Last Observation Carried Forward (LOCF) is not recommended. More robust missing data approaches (e.g., multiple imputation) should be considered.**

**-Response:** We sincerely appreciate the reviewer’s valuable suggestion regarding the handling of missing data. We have adopted multiple imputation to handle missing values for our primary and secondary outcomes to ensure the robustness of our statistical inferences.

Specifically, 20 imputed datasets will be generated, with the imputation model including baseline outcome values, age, T1DM duration, and treatment group as auxiliary variables. Results will be pooled across imputed datasets using Rubin’s rules.

We have revised the ‘**Methods > Statistical Analysis**’ section accordingly:

“*Missing data will be handled using multiple imputation, generating 20 imputed datasets with imputation models including baseline outcome values, age, T1DM duration, and treatment group. Results will be pooled using Rubin’s rules.*” (Page 23, Line 458-461)

**Results section should be rewritten according to the modified method and statistical analysis.**

**-Response:** We thank the reviewer for this comment. We would like to clarify that this manuscript is a study protocol describing the design and methodology of an ongoing trial, rather than a completed study report. As the trial is currently ongoing and data collection has not yet been completed, no clinical results are available at this stage.

Upon study completion, we will conduct all statistical analyses according to the finalized analytical plan described in the ‘**Methods**’ section, and the ‘**Results**’ section will be presented accordingly in the future study report.

We have ensured that the ‘**Methods**’ section has been fully updated to incorporate all reviewer-recommended refinements, and the planned analyses are now clearly specified.

**Comment 5: Donor Selection and Safety: Donor-recipient matching is mentioned but insufficiently described. Critical details missing: Infectious disease screening, Microbiological safety procedures. These are essential for FMT-based interventions.**

**-Response:** We sincerely appreciate your crucial comment regarding donor selection.The yFMT capsules are derived from healthy young donors aged 4-17 years. Sourced from a rigorously screened donor registry, donor-recipient matching is conducted utilizing a three-dimensional framework based on disease state, sex, and age. Crucially, all donor screening, infectious disease testing, and microbiological safety procedures are executed in strict accordance with *the Chinese expert consensus on screening and management of fecal microbiota transplantation donors (2022 edition)*^[1]^. This comprehensive safety protocol encompasses exhaustive blood and stool pathogen screening, the systematic exclusion of multidrug-resistant organisms, mental health evaluations, and capsule preparation conducted under Good Manufacturing Practice (GMP)-grade standards to guarantee maximal biosafety.

We have updated this rationale in the ‘**Methods >Procedures >Study intervention**’ sections：

“*All donor screening, infectious disease testing, and microbiological safety procedures comply with the Chinese expert consensus on fecal microbiota transplantation donor management[1], with dual informed consent obtained from both donors (when age-appropriate) and their legal guardians.*” (Page 11,12, Line 220-224)

**REFERENCES:**

[1] Society of Parenteral and Enteral Nutrition, Chinese Medical Association;Microecology Professional Committee of Shanghai Preventive Medicine Association.Chinese expert consensus on screening and management of fecal microbiotatransplantation donors (2022 edition). Chinese Journal of Gastrointestinal Surgery.(2022) 25(9):757-65. doi: 10.3760/cma.j.cn441530-20220606-00246

**Comment 6: Ethical Considerations: Ethical approval is reported . However: The use of pediatric donors raises ethical concerns. The manuscript should clarify the consent procedures and Safety monitoring protocols**

**-Response:** We sincerely thank you for this insightful comment concerning the ethics of pediatric donor recruitment. To fully address these concerns, we will safeguard the rights and safety of our young donors through the following specific measures:

**Informed Consent:**A dedicated dual informed consent process was implemented. Following a detailed explanation of all study-related information to the donors and their legal guardians, written informed consent was obtained from both the donors (when age-appropriate) and their legal guardians before enrollment.

**Safety Monitoring and Management:** In accordance with the Chinese expert consensus on screening and management of fecal microbiota transplantation donors (2022 edition), a comprehensive donor management system was established, comprising:

1. Dynamic Health Monitoring: Regular (e.g., bimonthly) face-to-face clinical health follow-ups for donors.
2. Clear Discontinuation Mechanism: Immediate eligibility suspension is triggered upon any health or microbiota abnormalities. Consecutive screening failures (twice) result in permanent disqualification and secure specimen disposal to guarantee recipient safety.
3. Data and Privacy Protection: All donor information is securely managed within an encrypted, restricted-access information system.

We have updated this rationale in the ‘**Methods >Procedures >Study intervention**’ section:

“*All donor screening, infectious disease testing, and microbiological safety procedures comply with the Chinese expert consensus on fecal microbiota transplantation donor management[1], with dual informed consent obtained from both donors (when age-appropriate) and their legal guardians.*” (Page 11,12, Line 220-224)

**REFERENCES:**

[1] Society of Parenteral and Enteral Nutrition, Chinese Medical Association;Microecology Professional Committee of Shanghai Preventive Medicine Association.Chinese expert consensus on screening and management of fecal microbiotatransplantation donors (2022 edition). Chinese Journal of Gastrointestinal Surgery.(2022) 25(9):757-65. doi: 10.3760/cma.j.cn441530-20220606-00246

**Comment 7: Intervention Details: The dosing regimen (12 capsules/day for 6 days) is clearly described. However: No clear justification is provided for dose or duration. Supporting references or preliminary data are needed.**

**-Response:** Thanks for your careful review and the helpful comments regarding our manuscript. This regimen is designed to deliver a total cumulative dose of approximately 2.0×10^^13^ viable bacteria over a single course. This dosage is highly consistent with a recently published oral fecal microbiota transplantation study targeting pediatric autoimmune disease (Crohn’s disease). In that study [1], a protocol of 10 capsules/day for 6 days (with a total bacterial load of 3.0×10^^13^) was employed, demonstrating well-established safety and efficacy profiles. Given that T1DM is likewise an autoimmune disorder and shares a comparable pathophysiological baseline of gut dysbiosis, adapting our dosage and duration based on this established framework is both rational and clinically conservative. Furthermore, the design of this protocol fully aligns with the “sufficient dose and multiple administrations” principle advocated in *the Chinese Expert Consensus on the Clinical Application of Fecal Microbiota Transplantation (2022 Edition)*[2] to ensure effective microbiota engraftment.

We have updated this rationale in the ‘**Methods >Procedures >Study intervention**’ section:

“*The administration protocol is as follows: 6 capsules are to be taken orally twice daily (a total of 12 capsules/day) under fasting conditions (at least 2 hours before or after meals) for six consecutive days,delivering a total cumulative dose of approximately 2.0×10^^13^ viable bacteria per course.*” (Page 12, Line 225-229)

**REFERENCES:**

[1] Zou B, Liu S, Dong C, et al. Fecal microbiota transplantation restores gut microbiota diversity in children with active Crohn’s disease: a prospective trial. *J Transl Med*. 2025;23(1):288.

[2] Society of Parenteral and Enteral Nutrition, Chinese Medical Association;Microecology Professional Committee of Shanghai Preventive Medicine Association.Chinese expert consensus on screening and management of fecal microbiotatransplantation donors (2022 edition). Chinese Journal of Gastrointestinal Surgery.(2022) 25(9):757-65. doi: 10.3760/cma.j.cn441530-20220606-00246

**Comment 8: Discussion: Lack of discussion regarding the effect of any possible confounding factors such as age, sex, and comorbidities that can affect the study findings.**

**-Response:** We thank the reviewer for this important comment. We agree that potential confounding factors such as age, sex, comorbidities, and T1DM duration should be discussed as they may influence the effecacy of yFMT.

**Regarding the statistical adjustment:** In the ‘**Methods > Statistics and Analysis**’ section, we have specified that linear mixed-effects models will be applied with group, time, and their interaction as fixed effects, the baseline value of the respective outcome as a covariate, and age and T1DM duration as additional covariates, with a random intercept for each participant.(as detailed in our response to Comment 4 and Comment 6). Sex and comorbidities, while also potential confounders, will not be included in the primary model due to sample size constraints (n=20) to avoid overfitting.

**Regarding the discussion of these factors:** We have revised the ‘**Discussion**’ section to address these potential confounders based on existing evidence::

1. **Age and T1DM duration:** Both factors may influence FMT response. Longer T1DM duration is inversely correlated with residual β-cell function [1], which may limit the potential for β-cell preservation, a key rationale for FMT in T1DM. Age may affect gut microbiota composition and immune function [2].
2. **Sex:** Sex differences in gut microbiota composition and FMT response have been documented [3], though the underlying mechanisms remain to be fully elucidated.
3. **Comorbidities:** Underlying comorbidities, particularly inflammatory bowel disease, immunosuppression, and neurodevelopmental conditions, have been identified as significant risk factors for poor FMT response in pediatric recipients [4].

Since our study protocol excludes patients with significant comorbidities that could affect gut microbiota (per the exclusion criteria), and we will adjust for age and T1DM duration in our statistical models, the confounding potential of these factors is minimized. However, we acknowledge that sex-stratified analyses will not be feasible due to the limited sample size, and we have noted this as a limitation. Future larger-scale confirmatory trials should systematically evaluate these factors as potential effect modifiers.

We have revised the ‘**Discussion**’ section as follows:

“*Additionally, several recipient-related factors may further complicate the interpretation of fecal microbiota transplantation outcomes in this pilot study. Longer T1DM duration has been associated with reduced residual β-cell function, potentially limiting the capacity for β-cell preservation[1]. Age may influence both gut microbiota composition and immune responsiveness[2]. Underlying comorbidities are also recognized as important determinants of fecal microbiota transplantation efficacy and safety[3]. Although age and T1DM duration were adjusted for in the statistical analyses and individuals with relevant comorbidities were excluded per protocol, the limited sample size (n=20) precludes robust subgroup analyses for these variables, as well as for other potential confounders. This further underscores a key limitation of the present pilot study and highlights the need for future adequately powered trials to systematically evaluate these potential effect modifiers.*” (Page 25,26, Line 506-517)

**REFERENCES:**

[1]. de Groot P, Nikolic T, Pellegrini S, et al. Faecal microbiota transplantation halts progression of human new-onset type 1 diabetes in a randomised controlled trial. Gut. 2021;70(1):92-105. doi:10.1136/gutjnl-2020-322630

[2]. Karmisholt Grosen A, Mikkelsen S, Aas Hindhede L, et al. Effects of clinical donor characteristics on the success of faecal microbiota transplantation for patients in Denmark with Clostridioides difficile infection: a single-centre, prospective cohort study. Lancet Microbe. 2025;6(5):101034. doi:10.1016/j.lanmic.2024.101034

[3]. Kellermayer R, Wu Q, Nagy-Szakal D, et al. Fecal Microbiota Transplantation Commonly Failed in Children With Co-Morbidities. J Pediatr Gastroenterol Nutr. 2022;74(2):227-235. doi:10.1097/MPG.0000000000003336

**Comment 9: Limitation and conclusion:**

**This study had limitations, primarily the issue of bias and possible confounding." Controlling for confounding variables is a critical step in conducting studies. Author should add an explanation regarding potential limitations for this trial.The authors should use potential confounding variables as covariates in a regression model. the conclusion should be rewritten based on the above point.**

**-Response:** We sincerely thank you for these insightful and critical comments. We would like to clarify that this is a study protocol describing the design of an ongoing trial, rather than a completed study report. As such, no study results are currently available for analysis.

Nevertheless, we fully agree that confounding control and limitation discussion are essential even at the protocol stage. We have addressed these points as follows:

1. **Prespecified confounding control in the statistical analysis:** In the ‘**Methods > Statistical Analysis**’ section, we have prespecified that linear mixed-effects models will be used for continuous secondary outcomes, with group, time, and their interaction as fixed effects, the baseline value of the respective outcome as a covariate, and age and T1DM duration as additional covariates. Sex and comorbidities, while also potential confounders, will not be included in the primary model due to sample size constraints to avoid overfitting.
2. **Discussion of potential confounding factors in the ‘Discussion’ section:** We have revised the ‘**Discussion**’ section to explicitly discuss recipient-related factors that may influence FMT outcomes based on existing evidence, including age, T1DM duration, sex, and comorbidities, along with appropriate supporting references.
3. **Acknowledgment of limitations:** We have also expanded the limitations paragraph to explicitly state that the small sample size may limit our ability to fully control for all potential confounders and to perform robust subgroup analyses, which is a recognized limitation of pilot study.

Upon study completion, we will conduct all analyses according to the prespecified statistical plan and formulate our conclusions with due consideration of these limitations.

We have revised the ‘**Discussion**’ section as follows:

“*However, our study is also limited by a relatively small sample size, single-center design, and relatively short(12-week) follow-up period, which might be insufficient to assess long-term glycemic benefit and sustained impact on residual islet β cell function. Additionally, several recipient-related factors may further complicate the interpretation of fecal microbiota transplantation outcomes in this pilot study. Longer T1DM duration has been associated with reduced residual β-cell function, potentially limiting the capacity for β-cell preservation[14]. Age may influence both gut microbiota composition and immune responsiveness[24]. Underlying comorbidities are also recognized as important determinants of fecal microbiota transplantation efficacy and safety[25]. Although age and T1DM duration were adjusted for in the statistical analyses and individuals with relevant comorbidities were excluded per protocol, the limited sample size (n=20) precludes robust subgroup analyses for these variables, as well as for other potential confounders. This further underscores a key limitation of the present pilot study and highlights the need for future adequately powered trials to systematically evaluate these potential effect modifiers.*” (Page 25,26, Line 503-517)

**REFERENCES:**

[1]. de Groot P, Nikolic T, Pellegrini S, et al. Faecal microbiota transplantation halts progression of human new-onset type 1 diabetes in a randomised controlled trial. Gut. 2021;70(1):92-105. doi:10.1136/gutjnl-2020-322630

[2]. Karmisholt Grosen A, Mikkelsen S, Aas Hindhede L, et al. Effects of clinical donor characteristics on the success of faecal microbiota transplantation for patients in Denmark with Clostridioides difficile infection: a single-centre, prospective cohort study. Lancet Microbe. 2025;6(5):101034. doi:10.1016/j.lanmic.2024.101034

[3]. Kellermayer R, Wu Q, Nagy-Szakal D, et al. Fecal Microbiota Transplantation Commonly Failed in Children With Co-Morbidities. J Pediatr Gastroenterol Nutr. 2022;74(2):227-235. doi:10.1097/MPG.0000000000003336

**Minor revision:**

**Comment 1:Language and Clarity: Minor grammatical issues and typographical inconsistencies are present. Professional language editing is recommended.**

**-Response:** We sincerely appreciate the reviewer’s valuable suggestion regarding the language quality. To address this concern, the manuscript has undergone comprehensive language editing and meticulous proofreading. We have carefully revised the text to correct minor grammatical errors, resolve typographical inconsistencies, and improve overall clarity and academic flow. We believe the readability and presentation of the manuscript have been significantly enhanced.

**Comment 2: Trial Registration: Trial registration appears to have occurred after study initiation. Clarification is needed to confirm compliance with prospective registration standards.**

**-Response:** We would like to clarify the timeline of our clinical trial to confirm full compliance with prospective registration standards.

**Trial Registration Date:** November 7, 2025 (Registration No. ChiCTR2500111955)

**Participant Recruitment Commencement:** November 20, 2025

Since the trial was officially registered prior to the enrollment of the first participant, this study strictly qualifies as a prospective registration under ICMJE guidelines.

Detailed information regarding this timeline can be found under the ‘**Abstract > Trial registration and Methods > Trial status**’ section：

1. **Abstract > Trial registration and Methods:** “Chinese Clinical Trial Registry identifier: ChiCTR2500111955 (November 7, 2025).” (Page 3, Line 54)
2. **Methods > Trial status:** “ Recruitment started on November 20, 2025, and is ongoing, with completion expected by July 31, 2026.” (Page 7, Line 130-132)

**Comment 3: Figures: Figures are referenced but should be improved for clarity and interpretation.**

**-Response:** Thank you for the valuable comment. We have thoroughly revised and optimized our figures to improve their clarity and interpretation:

First, the feasibility endpoints have been integrated into Figure 1, and Figure 3 has been modified to better adhere to standard reporting guidelines.

Furthermore, in accordance with PLOS ONE’s specific technical requirements, we have processed all figures using the journal's official optimization tool. This ensures that all images now meet the required benchmarks for resolution, typography, and contrast.

**Comment 4: Tables:-Author mention how data presented, method of data analysis, abbreviation and the Statistical tests used for comparison and the level of significance below each table and figure. …. Revise.**

**-Response:** We thank the reviewer for this important guidance on formatting. As this is a randomized controlled trial (RCT) protocol and the trial is currently ongoing, no clinical data or results tables/figures have been generated yet. Upon study completion, we will incorporate comprehensive footnotes (including data presentation formats, statistical tests, abbreviations, and significance levels) beneath every table and figure in the future full manuscript.

**Comment 5:Terminology and Abbreviations: Ensure consistent use of terminology (e.g., yFMT vs FMT). Consider reducing excessive abbreviations.**

**-Response:**We sincerely thank the reviewer for this constructive suggestion to improve the readability of our manuscript. We have carefully reviewed the entire manuscript to ensure consistent use of terminology (e.g., using ‘yFMT’ consistently for the youth-derived intervention) and have reduced excessive or non-standard abbreviations by defining them at first use and using full terms where appropriate to improve readability.

===========================================================

RESPONSES TO REVIEWER #3

===========================================================

**Comment 1: The authors should further clarify how yFMT may improve glycemic outcomes based on animal studies or previously published clinical evidence. In the Introduction, the discussion mainly focuses on the role of yFMT in preventing or delaying T1DM, whereas evidence supporting improvement of glycemic outcomes in individuals with established T1DM is limited. Since the current RCT focuses on glycemic improvement, more mechanistic and clinical evidence supporting this rationale should be added and discussed in greater detail.**

**-Response:** We sincerely appreciate your valuable suggestion. In accordance with your comments, we have outlined the underlying pathways based on animal studies, followed by a summary of recent clinical evidence revealing the efficacy of FMT in optimizing T1DM glycemic outcomes.

We have revised in the ‘**INTRODUCTION**’ section：

“*Evidence from animal studies have demonstrated that modulation of the gut microbiota can improved glycemic control through multiple mechanisms, including enhancement of intestinal barrier integrity, increased production of short-chain fatty acids, regulation of immune responses, and preservation of residual β-cell function[1,2]. Clinical evidence supports the potential of fecal microbiota transplantation in enhancing glycemic control among patients with T1DM. In a randomized controlled trial(RCT), both autologous and allogeneic fecal microbiota transplantation groups exhibited a reduction in glycated hemoglobin (HbA1c) at 12 months, without significant changes in insulin dosages[3]. Additionally, clinical studies demonstrate that washed microbiota transplantation significantly increases the time in range (TIR) and reduces glycemic variability among adults with T1DM[4,5].* ” (Page 4,5, Line 75-87)

**REFERENCES**

[1] Calcinaro F, Dionisi S, Marinaro M, Candeloro P, Bonato V, Marzotti S, et al. Oral probiotic administration induces interleukin-10 production and prevents spontaneous autoimmune diabetes in the non-obese diabetic mouse. *Diabetologia*. 2005;48(8):1565-75.

[2] Hänninen A, Toivonen R, Pöysti S, Belzer C, Plovier H, Ouwerkerk JP, et al. Akkermansia muciniphila induces gut microbiota remodelling and controls islet autoimmunity in NOD mice. *Gut*. 2018;67(8):1445-1453.

[3] de Groot P, Nikolic T, Pellegrini S, Sordi V, Imangaliyev S, Rampanelli E, et al. Faecal microbiota transplantation halts progression of human new-onset type 1 diabetes in a randomised controlled trial. *Gut*. 2021;70(1):92-105.

[4] Li YY, Zhu YX, Zhou Y, Qian C, Zhou J, Chen RR, et al. [Efficacy and safety of washed microbiota transplantation in the treatment of brittle diabetes]. *Chin J Diabetes*. 2020;12(12): 962-7. Chinese.

[5 Liu Q, Hua Y, He R, Xiang L, Li S, Zhang Y, et al. Restoration of intestinal secondary bile acid synthesis: A potential approach to improve pancreatic β cell function in type 1 diabetes. *Cell Rep Med*. 2025;6(5):102130.

**Comment 2: Please clarify the inclusion criteria defining suboptimal glycemic control, specifically HbA1c 7–14% and/or TIR <70%. In addition, the duration used for TIR assessment should be specified (e.g., 3 days, 14 days, or another period). This is important because HbA1c and TIR should ideally reflect comparable periods of glycemic assessment when used together to define suboptimal glycemia. Furthermore, the statistical analysis may benefit from subgroup analyses comparing participants with discordant glycemic metrics, such as:HbA1c ≥7.0% but TIR >70%; and HbA1c <7.0% but TIR <70%.**

**-Response:**Thank you for your valuable comments. We appreciate the opportunity to clarify our criteria and improve the manuscript. In this study, “suboptimal glycemic control” is defined as meeting either of the following criteria prior to screening:

1. HbA1c of 7.0-14.0% within the past three months.
2. 14-day CGM-derived TIR<70% within the past month.

We have clarified this information in ‘**Methods > Participants**’ section and T**able 1:**

**Methods > Participants:** “suboptimal glycemic outcomes,defined as HbA1c of 7.0-14.0% within the past three months or 14-day CGM-derived TIR<70% within the past month.” (Page 7, Line 138,139)

**Table 1:** “HbA1c between 7.0 to 14.0% (within past 3 months) or TIR < 70% (14-day CGM within the past month)”

**Comment 3: Please clarify whether participants aged exactly 18 years and 60 years were included in the eligibility criteria.**

**-Response:** Thanks for your question regarding the age eligibility criteria. We clarify that participants aged exactly 18 and 60 years were included in the study. To ensure absolute clarity, we have updated the text in **table 1**.- “Aged 18-60 years (inclusive)”

**Comment 4: More details should be provided regarding the optional course of open-label yFMT treatment. Specifically, please clarify how this extension phase was conducted, including eligibility, timing, dosing, and outcome assessment.**

**-Response:** Thank you for this constructive comment regarding the optional open-label extension phase. To ensure ethical equity, all consenting participants in the placebo group will be eligible to receive a complimentary course of open-label yFMT upon study unblinding, without any baseline glycemic or HbA1c criteria. This extension phase utilizes a treatment regimen and dosage identical to those of the yFMT group. Data collected during this open-label extension phase will be analyzed separately and are not included in the primary analysis of the randomized controlled trial. Post-treatment, participants are advised to undergo HbA1c testing and 14-day CGM monitoring at 1 and 3 months. Concurrently, they are instructed to self-report any subsequent adverse events via the 24-hour safety hotline.

We have revised in the ‘**Methods>Post-Study Procedures**’ section:

“*To ensure ethical equity, all consenting participants in the placebo group will be eligible to receive a complimentary course of open-label yFMT upon study unblinding, without any baseline glycemic or HbA1c criteria. This extension phase utilizes a treatment regimen and dosage identical to those of the yFMT group. Data from this open-label extension phase will be analyzed separately and are not included in the primary analysis. Post-treatment, participants are advised to undergo HbA1c testing and 14-day CGM monitoring at 1 and 3 months. Concurrently, they are instructed to self-report any subsequent adverse events via the 24-hour safety hotline*.” (Page 15, Line 284-291)

**Comment 5: Please clarify the definition and timing of the primary outcome assessment. It is currently unclear whether the primary endpoint refers to the absolute value measured at the end of the study or the change from baseline. The description appears inconsistent between the manuscript text and the tables.**

**-Response:** We thank the reviewer for this important clarification question. We apologize for the inconsistency in the description of the primary endpoint between the text and tables. We have now carefully revised and unified the definition throughout the manuscript.

The primary endpoint is defined as the composite achievement rate of TIR (time in range 3.9-10.0 mmol/L) >70% and TBR3.9 (time below range <3.9 mmol/L) <4% at 3 months (weeks 10-12) post-intervention, relative to baseline (days -17 to -3 during the run-in period). This time point was selected to assess the sustained glycemic effects of yFMT. The 1-month (weeks 2-4) assessment is retained as a secondary endpoint to evaluate early glycemic changes and track the trajectory of treatment response over time.

We have revised the ‘‘**Abstract > Methods and analysis and Methods > Endpoints**’ sections accordingly:

**Abstract > Methods and analysis:** “*The primary efficacy endpoint is the change from baseline in the rate of achieving the composite target of TIR > 70% and time below range <4% at 12 weeks post-randomization. Secondary efficacy endpoints include:(1) the change from baseline in the same composite achievement rate at 4 weeks post-intervention;*” (Page 2, Line 35-39)

**Methods > Endpoints:** “*The primary endpoint is change in the composite achievement rate of TIR>70% and TBR3.9 <4% at 3 months (Weeks 10-12) post-intervention compared to baseline (days -17 to -3 during the run-in period).*” (Page 15, Line 311-313)

“*The 1-month (Weeks 2-4) assessment is defined as a secondary endpoint to evaluate early glycemic changes*.” (Page 17, Line 316,317)

**Comment 6: The manuscript mentions a standardized diet during the run-in period. Please provide additional details regarding the dietary protocol, including composition, duration, monitoring, and whether adherence was assessed.**

**-Response:** We thank the reviewer for this comment. During the 14-day run-in period (days -17 to -3), participants are instructed to follow a standardized diet according to the Chinese Diabetes Dietary Guidelines (2017). Adherence is monitored through dietary records and regular follow-up communications via WeChat or outpatient visits.

We have revised the ‘**Methods > Procedures > Run-in Period and Baseline Assessments**’ section accordingly:

“*During this period, participants are instructed to follow a standardized diet according to the Chinese Diabetes Dietary Guidelines (2017). Dietary adherence is monitored through food records and regular follow-up communications via WeChat or outpatient visits.*” (Page 9, Line 159-162)

===========================================================

RESPONSES TO REVIEWER #4

===========================================================

**Comment 1: In overall, the protocol requires a detail description, from sample size calculation (according to the objectives), detail protocols for each laboratory measurement and research parameters.**

**-Response:**Thank you for the constructive comment. To address these requirements, we have fully elaborated on the sample size calculation, laboratory measurement protocols, and specific research parameters in the revised manuscript as detailed below.

1. **Regarding to sample size calculation:** As no prior data exist on achieving the composite glycemic target (TIR >70% with TBR3.9 <4%) following yFMT in adults with T1DM, a traditional sample size calculation based on an anticipated effect size was not feasible. The initial sample size is based on the minimum requirement of 10-15 cases specified for pilot trials. An interim analysis will be carried out to assess feasibility and safety, and to inform whether a sample size adjustment is warranted.

The detailed rationale for this adaptive design and sample size strategy can be found in the ‘**Methods>Sample Size and Pilot Trial Rationale**’ section:

“*This investigation is a pilot study. Its primary aim is not to establish the efficacy of yFMT, but to address the key uncertainties in this novel interventional field to inform the design of a future definitive clinical trial. The specific pilot objectives are to assess the feasibility (recruitment, adherence) and safety of the oral yFMT protocol, to evaluate the variability of the primary outcome, and to collect preliminary effect-size estimates for the planning of a subsequent pivotal trial.*

*As no prior data exist on achieving the composite glycemic target (TIR >70% with TBR3.9 <4%) following yFMT in adults with T1DM, a traditional sample size calculation based on an anticipated effect size was not feasible. The sample size for this trial was therefore determined in accordance with methodological best practices for pilot trial design. We referenced the guidance proposed by Wang et al., which indicates that a pilot study for a future definitive trial with 90% power and a two-sided alpha of 0.05 typically requires approximately 10 to 25 participants per group to provide meaningful parameter estimates for small-to-moderate effect sizes [23]. Based on this, and considering the common requirement for assessing primary feasibility objectives (e.g., recruitment rate, procedural acceptance), a sample size of 10 participants per group was set.*

*A pre-planned interim analysis will be conducted after the first 10 participants (50% of the total planned sample) have completed the 12-week follow-up. An independent Data Monitoring Committee (DMC) will review unblinded safety data to monitor for unacceptable AEs and will evaluate the primary composite endpoint to estimate the treatment effect for future sample size planning. No formal hypothesis testing will be performed, and no sample size re-estimation is planned for the current trial, consistent with its pilot nature. Based on the DMC’s review, the trial may continue as planned, be modified, or be terminated early if unacceptable safety concerns or clear futility are observed.*” (Page 20,21, Line 396-423)

2. **Regarding to laboratory measurement protocols:** We fully agree with the reviewer that detailed laboratory protocols are essential for reproducibility. In the revised manuscript, we have provided a comprehensive summary table in the ‘**Methods**’ section (**S2 File**) that lists all laboratory measurements, including the analytical method, units, and normal reference ranges. Blood samples will be processed within 1 hour of collection and stored at -80°C until analysis. Fecal samples will be collected using tubes containing preservation buffer to stabilize microbial composition at ambient temperature during transport, and subsequently stored at -80 °C for further microbiome and metabolomic analyses.

**REFERENCES**

[1] Wang Y, Wang LQ, Yang JW, Shi GX, Tu JF, Yan SY, et al. [Application and reflection on pilot study in acupuncture clinical research]. Zhongguo Zhen Jiu. 2021;41(3):325-9. Chinese.

===========================================================

We hope that these comprehensive revisions fully address the editor's and reviewers' concerns, and we look forward to hearing from you.

Sincerely yours,

Dr. Zhe Dai & Dr. Zhiqiang Li

Zhongnan Hospital of Wuhan University

Email: daizhe@znhospital.cn; Lizhiqiang@whu.edu.cn
