# Supplementary material for: Efficacy, safety, and feasibility of youth-derived fecal microbiota transplantation among adults with type 1 diabetes mellitus: A protocol of Pilot Randomized Controlled Trial
Source: PLoS One. 2026 Sep 21;21(9):e0343078. doi: 10.1371/journal.pone.0343078 (PMC13592622; doi:10.1371/journal.pone.0343078)
Supplement: Supplementary file 5 [file pone.0343078.s008.docx]

Dear editors,

Many thanks for the invitation. The authors have well addressed my concerns. I had no further comment and recommend for its publication.
