# Supplementary material for: Efficacy, safety, and feasibility of youth-derived fecal microbiota transplantation among adults with type 1 diabetes mellitus: A protocol of Pilot Randomized Controlled Trial
Source: PLoS One. 2026 Sep 21;21(9):e0343078. doi: 10.1371/journal.pone.0343078 (PMC13592622; doi:10.1371/journal.pone.0343078)
Supplement: Supplementary file 6 [file pone.0343078.s009.docx]

Dear Dr. Ze Xiang,

Academic Editor, PLOS ONE,

Thank you very much for giving us the opportunity to further revise our manuscript entitled “Efficacy, safety, and feasibility of Youth-derived Fecal Microbiota Transplantation among adults with Type 1 Diabetes Mellitus: A protocol of pilot randomized controlled trial” (PONE-D-26-05078).

We would also like to express our sincere gratitude to the editor and the reviewers for their constructive and helpful comments. We have carefully considered all remaining comments and addressed them point by point in the revised manuscript. All changes in the manuscript have been highlighted using the “Track Changes” function in the Word file labeled ‘Revised Manuscript with Track Changes’.

Responses to the remaining comments are detailed below.

**Response to reviewers**

===========================================================

**Reviewer 3: The authors have addressed the major concerns, and the manuscript has been substantially improved. A minor issue remains and should be addressed:**

**Comment 1. Please further clarify the strategy for handling missing data in the statistical analysis. Authors state that participants missing the final assessment will be classified as non-responders, while multiple imputation is also described for handling missing data. It seems to be a paradox, the authors should clarify.**

**-Response:** We thank the reviewer for raising this important point. We apologize for the lack of clarity in our original description. We have revised our statistical analysis plan to adopt a differentiated approach to handling missing data:

For the primary endpoint (binary outcome: achievement vs. non-achievement), participants without primary endpoint assessments at Week 12 will be considered non-responders using a non-responder imputation approach in the intention-to-treat analysis. This conservative approach was selected for the primary efficacy analysis.

For secondary continuous outcomes and covariates, missing data will be addressed using multiple imputation. Twenty imputed datasets will be generated using baseline outcome values, age, T1DM duration, and treatment group as predictors, and results will be pooled using Rubin’s rules.

This approach is not contradictory but reflects the different characteristics of the primary binary endpoint and secondary continuous outcomes.

We have revised the ‘METHODS > Statistics and Analysis’ section as follows:

“*Efficacy analyses will be performed in the intention-to-treat population. For the primary efficacy endpoint, participants with missing primary endpoint assessments will be considered non-responders using a non-responder imputation approach in the intention-to-treat analysis. Safety analyses will be performed in the safety analysis set.*” (Page 22, Line 430-433)

“*For secondary continuous outcomes and covariates, missing data will be addressed using multiple imputation. Twenty imputed datasets will be generated using baseline outcome values, age, T1DM duration, and treatment group as predictors, and results will be pooled using Rubin’s rules.*” (Page 23, Line 458-461)

==========================================================

We hope that these revisions fully address the editor's and reviewers' concerns, and we look forward to hearing from you.

Sincerely yours,

Dr. Zhe Dai & Dr. Zhiqiang Li

Zhongnan Hospital of Wuhan University

Email: daizhe@znhospital.cn; Lizhiqiang@whu.edu.cn
