# Supplementary material for: Efficacy, safety, and feasibility of youth-derived fecal microbiota transplantation among adults with type 1 diabetes mellitus: A protocol of Pilot Randomized Controlled Trial
Source: PLoS One. 2026 Sep 21;21(9):e0343078. doi: 10.1371/journal.pone.0343078 (PMC13592622; doi:10.1371/journal.pone.0343078)
Supplement: Peer review history

# Peer review: Decision Letter 0

**DOI:** 10.1371/journal.pone.0343078.r001

**Authors:** Ze Xiang

20 May 2026

Dear Dr. Dai,

Thank you for submitting your manuscript to PLOS ONE. After careful consideration, we feel that it has merit but does not fully meet PLOS ONE’s publication criteria as it currently stands. Therefore, we invite you to submit a revised version of the manuscript that addresses the points raised during the review process.

==============================

**The manuscript needs major revision according to the reviewers' comments.**

==============================

Please submit your revised manuscript by Jul 04 2026 11:59PM. If you will need more time than this to complete your revisions, please reply to this message or contact the journal office at plosone@plos.org. When you're ready to submit your revision, log on to https://www.editorialmanager.com/pone/ and select the 'Submissions Needing Revision' folder to locate your manuscript file.

- A letter that responds to each point raised by the academic editor and reviewer(s). You should upload this letter as a separate file labeled 'Response to Reviewers'.
- A marked-up copy of your manuscript that highlights changes made to the original version. You should upload this as a separate file labeled 'Revised Manuscript with Track Changes'.
- An unmarked version of your revised paper without tracked changes. You should upload this as a separate file labeled 'Manuscript'.

If you would like to make changes to your financial disclosure, please include your updated statement in your cover letter. Guidelines for resubmitting your figure files are available below the reviewer comments at the end of this letter.

If applicable, we recommend that you deposit your laboratory protocols in protocols.io to enhance the reproducibility of your results. Protocols.io assigns your protocol its own identifier (DOI) so that it can be cited independently in the future. For instructions see: https://journals.plos.org/plosone/s/submission-guidelines#loc-laboratory-protocols. Additionally, PLOS ONE offers an option for publishing peer-reviewed Lab Protocol articles, which describe protocols hosted on protocols.io. Read more information on sharing protocols at https://plos.org/protocols?utm_medium=editorial-email&utm_source=authorletters&utm_campaign=protocols.

As the corresponding author, your ORCID iD is verified in the submission system and will appear in the published article. PLOS supports the use of ORCID, and we encourage all coauthors to register for an ORCID iD and use it as well. Please encourage your coauthors to verify their ORCID iD within the submission system before final acceptance, as unverified ORCID iDs will not appear in the published article. *Only* the individual author can complete the verification step; PLOS staff *cannot* verify ORCID iDs on behalf of authors.

We look forward to receiving your revised manuscript.

Kind regards,

Ze Xiang

Academic Editor

PLOS One

Journal Requirements:

When submitting your revision, we need you to address these additional requirements.

1. Please ensure that your manuscript meets PLOS ONE's style requirements, including those for file naming. The PLOS ONE style templates can be found at

https://journals.plos.org/plosone/s/file?id=wjVg/PLOSOne_formatting_sample_main_body.pdf and

https://journals.plos.org/plosone/s/file?id=ba62/PLOSOne_formatting_sample_title_authors_affiliations.pdf

2. We note that you have indicated that there are restrictions to data sharing for this study. PLOS only allows data to be available upon request if there are legal or ethical restrictions on sharing data publicly. For more information on unacceptable data access restrictions, please see http://journals.plos.org/plosone/s/data-availability#loc-unacceptable-data-access-restrictions.

Before we proceed with your manuscript, please address the following prompts:

a) If there are ethical or legal restrictions on sharing a de-identified data set, please explain them in detail (e.g., data contain potentially identifying or sensitive patient information, data are owned by a third-party organization, etc.) and who has imposed them (e.g., a Research Ethics Committee or Institutional Review Board, etc.). Please also provide contact information for a data access committee, ethics committee, or other institutional body to which data requests may be sent.

b) If there are no restrictions, please upload the minimal anonymized data set necessary to replicate your study findings to a stable, public repository and provide us with the relevant URLs, DOIs, or accession numbers. For a list of recommended repositories, please see

https://journals.plos.org/plosone/s/recommended-repositories. You also have the option of uploading the data as Supporting Information files, but we would recommend depositing data directly to a data repository if possible.

We will update your Data Availability statement on your behalf to reflect the information you provide.

3. If the reviewer comments include a recommendation to cite specific previously published works, please review and evaluate these publications to determine whether they are relevant and should be cited. There is no requirement to cite these works unless the editor has indicated otherwise.

[Note: HTML markup is below. Please do not edit.]

Reviewers' comments:

Reviewer's Responses to Questions

**Comments to the Author**

1. Does the manuscript provide a valid rationale for the proposed study, with clearly identified and justified research questions?

Reviewer #1: Yes

Reviewer #2: Yes

Reviewer #3: Yes

Reviewer #4: No

**********

2. Is the protocol technically sound and planned in a manner that will lead to a meaningful outcome and allow testing the stated hypotheses?

Reviewer #1: Yes

Reviewer #2: Yes

Reviewer #3: Yes

Reviewer #4: No

**********

3. Is the methodology feasible and described in sufficient detail to allow the work to be replicable?

Reviewer #1: Yes

Reviewer #2: Yes

Reviewer #3: Yes

Reviewer #4: No

**********

4. Have the authors described where all data underlying the findings will be made available when the study is complete?

The PLOS Data policy

Reviewer #1: No

Reviewer #2: No

Reviewer #3: Yes

Reviewer #4: Yes

**********

5. Is the manuscript presented in an intelligible fashion and written in standard English?

Reviewer #1: Yes

Reviewer #2: No

Reviewer #3: Yes

Reviewer #4: Yes

**********

Please use the space provided to explain your answers to the questions above and, if applicable, provide comments about issues authors must address before this protocol can be accepted for publication. You may also include additional comments for the author, including concerns about research or publication ethics.

You may also provide optional suggestions and comments to authors that they might find helpful in planning their study.

Reviewer #1: The group plan to conduct a randomized controlled pilot study to evaluate the efficacy and safety of orally administered yFMT capsules in adults with T1DM.

1. Line 271. The primary endpoint was the composite achievement rate of TIR > 70% +TBR < 4%. Please clarify this definition with more specific definition.

2. Line 358. The pre-planned interim analysis will be conducted. Please clarify and be more specific on what interim analysis will be conducted and what action will be taken based on the pre=planned interim analysis.

3. Line 373. To increase the transparency, please clarify the specifications for GEE, such as the correlation structure and so on.

4. Line 376. It is unclear what covariates will be adjusted in the model. Please clarify this.

5. Line 378. The missing data for the primary endpoint will be imputed with the last observation. This is concerning to impute the primary endpoint!

Reviewer #2: This manuscript describes a single-center, randomized, double-blind, placebo-controlled pilot trial evaluating the efficacy and safety of youth-derived fecal microbiota transplantation (yFMT) in adults with type 1 diabetes mellitus (T1DM). The study aims to assess feasibility, safety, and preliminary efficacy using continuous glucose monitoring (CGM)-based composite endpoints over a 12-week follow-up period.

1. Scientific Rationale and Novelty: The concept of youth-derived FMT (yFMT) is innovative and relevant. However, the rationale for selecting young donors (4–17 years) requires stronger justification. The claim that this age group represents “peak microbiota diversity” is not sufficiently supported by direct evidence.

2. Study Design and Sample Size: The study is appropriately designed as a randomized controlled pilot trial. The sample size (n=10 per group) is justified based on pilot study guidance . However: The small sample limits interpretability of efficacy outcomes. The manuscript should clearly emphasize feasibility objectives (e.g., recruitment, adherence).

3. Primary Endpoint: The composite endpoint (TIR >70% and TBR <4%) is clinically meaningful. Clarification is needed on: Whether both criteria must be achieved simultaneously. Statistical handling of the composite endpoint.

4. Statistical Analysis:

-Did the author check the normality, which test has been used?

-The author also should mention the Statistical tests used and the level of significance in the Statistical Analysis and elaborate the abbreviated name below each table.

-The authors must explain how they controlled confounding factors, as it seems that adjustment of confounders is not considered. Did they assess multicollinearity?

-The proposed use of mixed-effects models and generalized estimating equations is appropriate. However: The use of Last Observation Carried Forward (LOCF) is not recommended. More robust missing data approaches (e.g., multiple imputation) should be considered.

- Results section should be rewritten according to the modified method and statistical analysis.

5. Donor Selection and Safety: Donor-recipient matching is mentioned but insufficiently described. Critical details missing: Infectious disease screening, Microbiological safety procedures. These are essential for FMT-based interventions.

6. Ethical Considerations: Ethical approval is reported . However: The use of pediatric donors raises ethical concerns. The manuscript should clarify the consent procedures and Safety monitoring protocols

7. Intervention Details: The dosing regimen (12 capsules/day for 6 days) is clearly described. However: No clear justification is provided for dose or duration. Supporting references or preliminary data are needed.

8. Discussion: Lack of discussion regarding the effect of any possible confounding factors such as age, sex, and comorbidities that can affect the study findings.

9. Limitation and conclusion:

- This study had limitations, primarily the issue of bias and possible confounding." Controlling for confounding variables is a critical step in conducting studies. Author should add an explanation regarding potential limitations for this trial.

-The authors should use potential confounding variables as covariates in a regression model.

- the conclusion should be rewritten based on the above point.

Minor Comments

1. Language and Clarity: Minor grammatical issues and typographical inconsistencies are present. Professional language editing is recommended.

2. Trial Registration: Trial registration appears to have occurred after study initiation . Clarification is needed to confirm compliance with prospective registration standards.

3. Figures: Figures are referenced but should be improved for clarity and interpretation.

4. Tables:-Author mention how data presented, method of data analysis, abbreviation and the Statistical tests used for comparison and the level of significance below each table and figure. …. Revise.

5. Terminology and Abbreviations: Ensure consistent use of terminology (e.g., yFMT vs FMT). Consider reducing excessive abbreviations.

Reviewer #3: Many thanks for inviting me to review this randomized controlled trial focusing on the efficacy and safety of orally administered yFMT capsules in adults with T1DM. The manuscript is generally well written and presented in standard English. The study design is appropriate and follows a standard RCT framework. However, before publication, several issues should be addressed:

1. The authors should further clarify how yFMT may improve glycemic outcomes based on animal studies or previously published clinical evidence. In the Introduction, the discussion mainly focuses on the role of yFMT in preventing or delaying T1DM, whereas evidence supporting improvement of glycemic outcomes in individuals with established T1DM is limited. Since the current RCT focuses on glycemic improvement, more mechanistic and clinical evidence supporting this rationale should be added and discussed in greater detail.

2. Please clarify the inclusion criteria defining suboptimal glycemic control, specifically HbA1c 7–14% and/or TIR <70%. In addition, the duration used for TIR assessment should be specified (e.g., 3 days, 14 days, or another period). This is important because HbA1c and TIR should ideally reflect comparable periods of glycemic assessment when used together to define suboptimal glycemia. Furthermore, the statistical analysis may benefit from subgroup analyses comparing participants with discordant glycemic metrics, such as:

HbA1c ≥7.0% but TIR >70%; and

HbA1c <7.0% but TIR <70%.

3. Please clarify whether participants aged exactly 18 years and 60 years were included in the eligibility criteria.

4. More details should be provided regarding the optional course of open-label yFMT treatment. Specifically, please clarify how this extension phase was conducted, including eligibility, timing, dosing, and outcome assessment.

5. Please clarify the definition and timing of the primary outcome assessment. It is currently unclear whether the primary endpoint refers to the absolute value measured at the end of the study or the change from baseline. The description appears inconsistent between the manuscript text and the tables.

6. The manuscript mentions a standardized diet during the run-in period. Please provide additional details regarding the dietary protocol, including composition, duration, monitoring, and whether adherence was assessed.

Reviewer #4: In overall, the protocol requires a detail description, from sample size calculation (according to the objectives), detail protocols for each laboratory measurement and research parameters.

**********

what does this mean?). If published, this will include your full peer review and any attached files.

If you choose “no”, your identity will remain anonymous but your review may still be made public.

**Do you want your identity to be public for this peer review?** For information about this choice, including consent withdrawal, please see our Privacy Policy

Reviewer #1: No

Reviewer #2: **Yes:**  Rehab H. Werida, Ph.D. Damanhour University, Egypt.

Reviewer #3: No

Reviewer #4: No

**********

[NOTE: If reviewer comments were submitted as an attachment file, they will be attached to this email and accessible via the submission site. Please log into your account, locate the manuscript record, and check for the action link "View Attachments". If this link does not appear, there are no attachment files.]

To ensure your figures meet our technical requirements, please review our figure guidelines: https://journals.plos.org/plosone/s/figures

You may also use PLOS’s free figure tool, NAAS, to help you prepare publication quality figures: https://journals.plos.org/plosone/s/figures#loc-tools-for-figure-preparation.

NAAS will assess whether your figures meet our technical requirements by comparing each figure against our figure specifications.

# Author comment: Author response to Decision Letter 1

**DOI:** 10.1371/journal.pone.0343078.r002

3 Jul 2026

Dear Dr. Ze Xiang,

Academic Editor, PLOS ONE,

Thank you very much for giving us the opportunity to revise our manuscript entitled “Efficacy, safety, and feasibility of Youth-derived Fecal Microbiota Transplantation among adults with Type 1 Diabetes Mellitus: A protocol of pilot randomized controlled trial” (PONE-D-26-05078).

We would also like to express our sincere gratitude to the reviewers for their constructive, thorough, and helpful comments. We have carefully addressed all requirements and reviewers’ comments in the revised manuscript. All changes in the manuscript have been highlighted using the “Track Changes” function in the Word file labeled ‘Revised Manuscript with Track Changes’.

The point-by-point responses to the Journal Requirements and Reviewers’ comments are detailed below.

Part 1: Response to Journal Requirements

To the Editor:

We have carefully reviewed the Journal Requirements and have ensured that the manuscript complies with all of them. Our specific responses are as follows:

Requirement 1: Please ensure that your manuscript meets PLOS ONE's style requirements, including those for file naming.

-Response: Thank you for the reminder. We have thoroughly reviewed the PLOS ONE style templates and carefully revised the manuscript’s formatting, layout, and file naming (including figures and tables) to ensure full compliance with the guidelines.

Requirement 2: We note that you have indicated that there are restrictions to data sharing for this study. PLOS only allows data to be available upon request if there are legal or ethical restrictions on sharing data publicly.

-Response: We thank you for raising this important issue regarding data sharing. We have carefully reviewed PLOS ONE's data availability policy and have addressed your concerns as follows:

As this manuscript is a study protocol, the clinical dataset has not yet been generated. Upon study completion, data will be available upon reasonable request, subject to corresponding author (Zhe Dai, daizhe@znhospital.cn).

We have revised in the ‘Data Availability’ section as follows:

“As this manuscript is a study protocol, the clinical dataset has not yet been generated. Upon study completion, data will be available upon reasonable request, subject to corresponding author (Zhe Dai, daizhe@znhospital.cn).” (Page 27, Line 535-537).

Requirement 3: If the reviewer comments include a recommendation to cite specific previously published works, please review and evaluate these publications to determine whether they are relevant and should be cited. There is no requirement to cite these works unless the editor has indicated otherwise.

-Response: We acknowledge the journal's policy regarding citation recommendations. We confirm that the reviewers did not suggest specific citations in this round of review. Furthermore, we have carefully scrutinized the reference list in the revised manuscript to ensure that all cited works are directly relevant to our study and contribute to the scientific context.

Part 2: Response to reviewers

===========================================================

RESPONSES TO REVIEWER #1

===========================================================

Comment 1: Line 271. The primary endpoint was the composite achievement rate of TIR > 70% +TBR < 4%. Please clarify this definition with more specific definition.

-Response: Thank you for this valuable comment. We have revised the manuscript to provide a precise definition of the primary endpoint.The primary endpoint is the composite achievement rate of TIR (time in range 3.9-10.0 mmol/L) >70% and TBR3.9 (time below range <3.9 mmol/L) <4% at 3 months (weeks 10-12) post-intervention, relative to baseline (days -17 to -3 during the run-in period).

We have corrected our expression in the ‘Methods > Endpoints’ section :

“The primary endpoint is change in the composite achievement rate of TIR>70% and TBR3.9 <4% at 3 months (Weeks 10-12) post-intervention compared to baseline (days -17 to -3 during the run-in period).” (Page 16, Line 311-313).

Comment 2: Line 358. The pre-planned interim analysis will be conducted. Please clarify and be more specific on what interim analysis will be conducted and what action will be taken based on the pre-planned interim analysis.

-Response: Thank you for this important methodological comment. We apologize for the lack of clarity in our original description.

We have revised the relevant section to specify that the pre-planned interim analysis will be conducted after the first 10 participants (50% of the total sample) have completed the 12-week follow-up, with the following explicit objectives:

(1)Safety monitoring: The independent Data Monitoring Committee (DMC) will review all unblinded safety data (adverse events, severe adverse events, and hypoglycemic episodes) to determine whether the trial should continue, be modified, or be terminated prematurely due to unacceptable toxicity or safety signals.

(2)Effect size estimation for future trials: The DMC will also review the unblinded primary endpoint data (composite achievement rate of TIR >70% with TBR3.9 <4%) to provide a preliminary estimate of the treatment effect (with 95% confidence intervals). This estimate will not be used to modify the sample size of the current trial, given its pilot nature (n=20), but will serve as the basis for sample size calculations in a future confirmatory randomized controlled trial.

(3)Futility assessment (exploratory): If the observed treatment effect in the intervention group shows no directionally favorable trend toward the composite endpoint compared with the placebo group, the DMC may recommend early termination to avoid exposing additional participants to an ineffective intervention.

Importantly, no formal hypothesis testing will be performed at the interim analysis, and the type I error rate will not be consumed, as the current trial is designed as a pilot study rather than a confirmatory trial. The final analysis will remain definitive.

We have revised in the ‘Methods > Sample Size and Pilot Trial Rationale’ section as follows:

“A pre-planned interim analysis will be conducted after the first 10 participants (50% of the total planned sample) have completed the 12-week follow-up. An independent Data Monitoring Committee (DMC) will review unblinded safety data to monitor for unacceptable AEs and will evaluate the primary composite endpoint to estimate the treatment effect for future sample size planning. No formal hypothesis testing will be performed, and no sample size re-estimation is planned for the current trial, consistent with its pilot nature. Based on the DMC’s review, the trial may continue as planned, be modified, or be terminated early if unacceptable safety concerns or clear futility are observed.” (Page 21, Line 415-423)

Comment 3: Line 373. To increase the transparency, please clarify the specifications for GEE, such as the correlation structure and so on.

-Response: Thank you for this important methodological comment. We agree that greater transparency regarding the correlation structure is necessary.

We have revised our statistical analysis plan as follows:

For the primary efficacy endpoint—the composite glycemic target achievement rate (TIR >70% with TBR3.9<4%) measured at two post-baseline time points (Week 4 and Week 12) —we will employ a generalized estimating equation (GEE) model with a binary distribution, logit link function, and an exchangeable correlation structure, which assumes equal correlation between repeated measurements within the same participant. The model will include group (yFMT vs. placebo), time (Week 4 vs. Week 12), and the group-by-time interaction as fixed effects, with the corresponding baseline value as a covariate when applicable. The primary inferential focus will be on the group effect across both time points, while the interaction term will be used to assess whether the treatment effect varies over time.

Given the small sample size (n=20), we will also perform Fisher’s exact test as a sensitivity analysis at each individual time point to confirm the robustness of the findings. The results of both analyses will be reported.

We have revised in the ‘Methods > Statistics and Analysis’ section:

“For the primary efficacy endpoint assessed at Week 4 and Week 12 post-intervention, a generalized estimating equation (GEE) model with a binary distribution, logit link function, and an exchangeable correlation structure will be employed. The model includes group, time, and their interaction as fixed effects, with baseline composite achievement status as covariates as appropriate. The group effect averaged across both time points will serve as the primary inferential basis. Given the pilot nature of the study (n=20), Fisher’s exact test will additionally be performed at each time point as a sensitivity analysis to ensure robustness.” (Page 22, Line 436-443)

Comment 4: Line 376. It is unclear what covariates will be adjusted in the model. Please clarify this.

-Response: Thank you for this comment. We have revised the statistical analysis plan to explicitly define the covariates to be adjusted in the linear mixed-effects models.

For the primary efficacy analysis—the composite glycemic target achievement rate (TIR >70% with TBR3.9 <4%) analyzed using the GEE model—no covariates other than the baseline composite achievement status will be adjusted, given the small sample size (n=20) and the pilot nature of the study.

Group, time, and their interaction are structural fixed effects of the repeated-measures design, rather than additional covariates. The covariates adjusted in the LMM are listed below.

Covariate Rationale

Baseline value of the respective outcome To adjust for regression to the mean and increase statistical precision

Age Known to influence glycemic control and immune function in T1DM

T1DM duration Longer duration is associated with lower residual β-cell function and may affect C-peptide outcomes

Importantly, to avoid model overfitting given the limited sample size (n=20), we adhere to the rule of thumb of ≤1 covariate per 5-10 participants for the primary model. The full covariate-adjusted model will be used for the primary analysis, while a parsimonious model adjusting only for baseline value, group, and time will be performed as a sensitivity analysis to confirm robustness.

We have revised the manuscript in the ‘Methods > Statistics and Analysis’ section as follows:

“For continuous secondary outcomes, linear mixed-effects models will be applied with group, time, and their interaction as fixed effects, the baseline value of the respective outcome as a covariate, and age and T1DM duration as additional covariates, with a random intercept for each participant. Multicollinearity will be assessed using the variance inflation factor (VIF), with VIF >10 indicating significant collinearity. Given the limited sample size, a parsimonious sensitivity model including only baseline value, group, and time will also be performed to assess robustness against overfitting.” (Page 22,23, Line 443-450)

Comment 5: Line 378. The missing data for the primary endpoint will be imputed with the last observation. This is concerning to impute the primary endpoint!

-Response: Thank you for raising this important methodological concern. We fully acknowledge that the last observation carried forward (LOCF) method has inherent limitations, particularly the assumption that participants’ outcomes remain unchanged after dropout, which may introduce bias.

We have adopted multiple imputation to handle missing values. Specifically, 20 imputed datasets will be generated, with the imputation model including baseline outcome values, age, T1DM duration, and treatment group as auxiliary variables. Results will be pooled across imputed datasets using Rubin’s rules.

We have revised the manuscript in the ‘Methods > Statistics and Analysis’ section as follows:

“Missing data will be handled using multiple imputation, generating 20 imputed datasets with imputation models including baseline outcome values, age, T1DM duration, and treatment group. Results will be pooled using Rubin’s rules.” (Page 23, Line 458-461)

===========================================================

RESPONSES TO REVIEWER #2

===========================================================

Major revisions:

Comment 1: Scientific Rationale and Novelty: The concept of youth-derived FMT (yFMT) is innovative and relevant. However, the rationale for selecting young donors (4–17 years) requires stronger justification. The claim that this age group represents “peak microbiota diversity” is not sufficiently supported by direct evidence.

-Response: We thank the reviewer for this critical comment. We agree that the original claim of “peak microbiota diversity” lacked sufficient direct evidence, and we have removed this statement.

Our selection of donors aged 4-17 years is now supported by evidence from two complementary lines:

1.First, longitudinal studies have demonstrated that the gut microbiota during childhood and adolescence exhibits greater temporal stability compared to both early infancy and older age groups [1,2]. This stability is theoretically advantageous for successful engraftment, as strains stably maintained in the donor are more likely to persist in the recipient [3].

2.Second, animal studies consistently show that young donor microbiota confers metabolic and immune benefits when transplanted into aged or diseased recipients. For instance, young-to-old FMT in mice reduced body weight, prevented fat accumulation, improved frailty, and reversed age-related inflammation in the brain and retina [4,5]. In sex-matched mouse models, early-life FMT from children induced long-lasting effects on adipose tissue function and systemic glucose homeostasis that persisted into adulthood [6].

While direct evidence linking young donor age to superior outcomes specifically in T1DM remains to be established—which is precisely what our pilot study aims to generate—the convergence of ecological rationale and animal data provides a strong justification for our donor selection strategy.

We have revised the manuscript accordingly in the ‘INTRODUCTION’ section:

“The yFMT capsules are prepared from rigorously screened healthy young donors (aged 4-17 years), selected for the relatively high temporal stability of the gut microbiota during this developmental window.”(Page 5, Line 101-103)

We also have revised the manuscript accordingly in the ‘Methods>Study intervention’ section:

“The yFMT capsules are manufactured by Meiyitian Biomedical Co., Ltd. (Wuhan, Hubei, China) and prepared from rigorously screened healthy young donors (aged 4-17 years), selected for the relatively high temporal stability of the gut microbiota during this developmental window.”(Page 11, Line 216-219)

We also have revised the manuscript accordingly in the ‘DISCUSSION’ section:

“The rationale for selecting young donors (aged 4-17 years) is grounded in the relatively high gut microbiota temporal stability characteristic of this developmental window.”(Page 24, Line 486-488).

REFERENCES:

[1]Yatsunenko T, Rey FE, Manary MJ, et al. Human gut microbiome viewed across age and geography. Nature. 2012;486(7402):222-227.

[2] Wu J, Shen H, Lv Y, et al. Age over sex: evaluating gut microbiota differences in healthy Chinese populations. Front Microbiol. 2024;15:1412991.

[3] Schmidt TSB, Li SS, Maistrenko OM, et al. Drivers and determinants of strain dynamics following fecal microbiota transplantation. Nat Med.2022;28(9):1902-1912.

[4] Parker A, Romano S, Ansorge R, et al. Fecal microbiota transfer between young and aged mice reverses hallmarks of the aging gut, eye, and brain. Microbiome. 2022;10(1):68.

[5] Xie J, Kim T, Liu Z, et al. Young gut microbiota transplantation improves the metabolic health of old mice. mSystems. 2025;10(6):e0160124.

[6] La Rosa F, Guzzardi MA, Pardo-Tendero M, et al. Effects of children’s microbiota on adipose and intestinal development in sex-matched mice persist into adulthood following a single fecal microbiota transplantation. Mol Metab. 2025;97:102157.

Comment 2: Study Design and Sample Size: The study is appropriately

Attachment Submitted filename: Response to Reviewers.docx

# Peer review: Decision Letter 1

**DOI:** 10.1371/journal.pone.0343078.r003

**Authors:** Ze Xiang

6 Aug 2026

Dear Dr. Dai,

Thank you for submitting your manuscript to PLOS ONE. After careful consideration, we feel that it has merit but does not fully meet PLOS ONE’s publication criteria as it currently stands. Therefore, we invite you to submit a revised version of the manuscript that addresses the points raised during the review process.

**The manuscript should be revised according to the reviewers' comments.**

Please submit your revised manuscript by Sep 20 2026 11:59PM. If you will need more time than this to complete your revisions, please reply to this message or contact the journal office at plosone@plos.org. When you're ready to submit your revision, log on to https://www.editorialmanager.com/pone/ and select the 'Submissions Needing Revision' folder to locate your manuscript file.

- A letter that responds to each point raised by the academic editor and reviewer(s). You should upload this letter as a separate file labeled 'Response to Reviewers'.
- A marked-up copy of your manuscript that highlights changes made to the original version. You should upload this as a separate file labeled 'Revised Manuscript with Track Changes'.
- An unmarked version of your revised paper without tracked changes. You should upload this as a separate file labeled 'Manuscript'.

If you would like to make changes to your financial disclosure, please include your updated statement in your cover letter. Guidelines for resubmitting your figure files are available below the reviewer comments at the end of this letter.

If applicable, we recommend that you deposit your laboratory protocols in protocols.io to enhance the reproducibility of your results. Protocols.io assigns your protocol its own identifier (DOI) so that it can be cited independently in the future. For instructions see: https://journals.plos.org/plosone/s/submission-guidelines#loc-laboratory-protocols. Additionally, PLOS ONE offers an option for publishing peer-reviewed Lab Protocol articles, which describe protocols hosted on protocols.io. Read more information on sharing protocols at https://plos.org/protocols?utm_medium=editorial-email&utm_source=authorletters&utm_campaign=protocols.

As the corresponding author, your ORCID iD is verified in the submission system and will appear in the published article. PLOS supports the use of ORCID, and we encourage all coauthors to register for an ORCID iD and use it as well. Please encourage your coauthors to verify their ORCID iD within the submission system before final acceptance, as unverified ORCID iDs will not appear in the published article. *Only* the individual author can complete the verification step; PLOS staff *cannot* verify ORCID iDs on behalf of authors.

We look forward to receiving your revised manuscript.

Kind regards,

Ze Xiang

Academic Editor

PLOS One

Journal Requirements:

If the reviewer comments include a recommendation to cite specific previously published works, please review and evaluate these publications to determine whether they are relevant and should be cited. There is no requirement to cite these works unless the editor has indicated otherwise.

Please review your reference list to ensure that it is complete and correct. If you have cited papers that have been retracted, please include the rationale for doing so in the manuscript text, or remove these references and replace them with relevant current references. Any changes to the reference list should be mentioned in the rebuttal letter that accompanies your revised manuscript. If you need to cite a retracted article, indicate the article’s retracted status in the References list and also include a citation and full reference for the retraction notice.

[Note: HTML markup is below. Please do not edit.]

Reviewers' comments:

Reviewer's Responses to Questions

**Comments to the Author**

1. Does the manuscript provide a valid rationale for the proposed study, with clearly identified and justified research questions?

Reviewer #1: Yes

Reviewer #3: Yes

**********

2. Is the protocol technically sound and planned in a manner that will lead to a meaningful outcome and allow testing the stated hypotheses?

Reviewer #1: Yes

Reviewer #3: Yes

**********

3. Is the methodology feasible and described in sufficient detail to allow the work to be replicable?

Reviewer #1: Yes

Reviewer #3: Yes

**********

4. Have the authors described where all data underlying the findings will be made available when the study is complete?

The PLOS Data policy

Reviewer #1: No

Reviewer #3: Yes

**********

5. Is the manuscript presented in an intelligible fashion and written in standard English?

Reviewer #1: Yes

Reviewer #3: Yes

**********

Please use the space provided to explain your answers to the questions above and, if applicable, provide comments about issues authors must address before this protocol can be accepted for publication. You may also include additional comments for the author, including concerns about research or publication ethics.

You may also provide optional suggestions and comments to authors that they might find helpful in planning their study.

Reviewer #1: Thanks for addressing all the raised comments or concerns. There is no further concerns from this reviewer.

Reviewer #3: The authors have addressed the major concerns, and the manuscript has been substantially improved. A minor issue remains and should be addressed:

1. Please further clarify the strategy for handling missing data in the statistical analysis. Authors state that participants missing the final assessment will be classified as non-responders, while multiple imputation is also described for handling missing data. It seems to be a paradox, the authors should clarify.

**********

what does this mean?). If published, this will include your full peer review and any attached files.

If you choose “no”, your identity will remain anonymous but your review may still be made public.

**Do you want your identity to be public for this peer review?** For information about this choice, including consent withdrawal, please see our Privacy Policy

Reviewer #1: No

Reviewer #3: No

**********

[NOTE: If reviewer comments were submitted as an attachment file, they will be attached to this email and accessible via the submission site. Please log into your account, locate the manuscript record, and check for the action link "View Attachments". If this link does not appear, there are no attachment files.]

To ensure your figures meet our technical requirements, please review our figure guidelines: https://journals.plos.org/plosone/s/figures

You may also use PLOS’s free figure tool, NAAS, to help you prepare publication quality figures: https://journals.plos.org/plosone/s/figures#loc-tools-for-figure-preparation.

NAAS will assess whether your figures meet our technical requirements by comparing each figure against our figure specifications.

Attachment Submitted filename: review.doc

# Author comment: Author response to Decision Letter 2

**DOI:** 10.1371/journal.pone.0343078.r004

9 Aug 2026

Dear Dr. Ze Xiang,

Academic Editor, PLOS ONE,

Thank you very much for giving us the opportunity to further revise our manuscript entitled “Efficacy, safety, and feasibility of Youth-derived Fecal Microbiota Transplantation among adults with Type 1 Diabetes Mellitus: A protocol of pilot randomized controlled trial” (PONE-D-26-05078).

We would also like to express our sincere gratitude to the editor and the reviewers for their constructive and helpful comments. We have carefully considered all remaining comments and addressed them point by point in the revised manuscript. All changes in the manuscript have been highlighted using the “Track Changes” function in the Word file labeled ‘Revised Manuscript with Track Changes’.

Responses to the remaining comments are detailed below.

Response to reviewers

===========================================================

Reviewer 3: The authors have addressed the major concerns, and the manuscript has been substantially improved. A minor issue remains and should be addressed:

Comment 1. Please further clarify the strategy for handling missing data in the statistical analysis. Authors state that participants missing the final assessment will be classified as non-responders, while multiple imputation is also described for handling missing data. It seems to be a paradox, the authors should clarify.

-Response: We thank the reviewer for raising this important point. We apologize for the lack of clarity in our original description. We have revised our statistical analysis plan to adopt a differentiated approach to handling missing data:

For the primary endpoint (binary outcome: achievement vs. non-achievement), participants without primary endpoint assessments at Week 12 will be considered non-responders using a non-responder imputation approach in the intention-to-treat analysis. This conservative approach was selected for the primary efficacy analysis.

For secondary continuous outcomes and covariates, missing data will be addressed using multiple imputation. Twenty imputed datasets will be generated using baseline outcome values, age, T1DM duration, and treatment group as predictors, and results will be pooled using Rubin’s rules.

This approach is not contradictory but reflects the different characteristics of the primary binary endpoint and secondary continuous outcomes.

We have revised the ‘METHODS > Statistics and Analysis’ section as follows:

“Efficacy analyses will be performed in the intention-to-treat population. For the primary efficacy endpoint, participants with missing primary endpoint assessments will be considered non-responders using a non-responder imputation approach in the intention-to-treat analysis. Safety analyses will be performed in the safety analysis set.” (Page 22, Line 430-433)

“For secondary continuous outcomes and covariates, missing data will be addressed using multiple imputation. Twenty imputed datasets will be generated using baseline outcome values, age, T1DM duration, and treatment group as predictors, and results will be pooled using Rubin’s rules.” (Page 23, Line 458-461)

==========================================================

We hope that these revisions fully address the editor's and reviewers' concerns, and we look forward to hearing from you.

Sincerely yours,

Dr. Zhe Dai & Dr. Zhiqiang Li

Zhongnan Hospital of Wuhan University

Email: daizhe@znhospital.cn; Lizhiqiang@whu.edu.cn

Attachment Submitted filename: Response_to_Reviewers_auresp_2.docx

# Peer review: Decision Letter 2

**DOI:** 10.1371/journal.pone.0343078.r005

**Authors:** Ze Xiang

24 Aug 2026

Efficacy, safety, and feasibility of Youth-derived Fecal MicrobiotaTransplantation among adults with Type 1 Diabetes Mellitus: A protocol of pilot randomized controlled trial

PONE-D-26-05078R2

Dear Dr. Dai,

We’re pleased to inform you that your manuscript has been judged scientifically suitable for publication and will be formally accepted for publication once it meets all outstanding technical requirements.

Within one week, you’ll receive an e-mail detailing the required amendments. When these have been addressed, you’ll receive a formal acceptance letter and your manuscript will be scheduled for publication.

An invoice will be generated when your article is formally accepted. Please note, if your institution has a publishing partnership with PLOS and your article meets the relevant criteria, all or part of your publication costs will be covered. Please make sure your user information is up-to-date by logging into Editorial Manager at Editorial Manager® and clicking the ‘Update My Information' link at the top of the page. For questions related to billing, please contact billing support.

If your institution or institutions have a press office, please notify them about your upcoming paper to help maximize its impact. If they’ll be preparing press materials, please inform our press team as soon as possible -- no later than 48 hours after receiving the formal acceptance. Your manuscript will remain under strict press embargo until 2 pm Eastern Time on the date of publication. For more information, please contact onepress@plos.org.

Kind regards,

Ze Xiang

Academic Editor

PLOS One

Additional Editor Comments (optional):

Reviewers' comments:

Reviewer's Responses to Questions

**Comments to the Author**

1. Does the manuscript provide a valid rationale for the proposed study, with clearly identified and justified research questions?

Reviewer #1: Yes

Reviewer #3: Yes

**********

2. Is the protocol technically sound and planned in a manner that will lead to a meaningful outcome and allow testing the stated hypotheses?

Reviewer #1: Yes

Reviewer #3: Yes

**********

3. Is the methodology feasible and described in sufficient detail to allow the work to be replicable?

Reviewer #1: Yes

Reviewer #3: Yes

**********

4. Have the authors described where all data underlying the findings will be made available when the study is complete?

The PLOS Data policy

Reviewer #1: No

Reviewer #3: Yes

**********

5. Is the manuscript presented in an intelligible fashion and written in standard English?

Reviewer #1: Yes

Reviewer #3: Yes

**********

Please use the space provided to explain your answers to the questions above and, if applicable, provide comments about issues authors must address before this protocol can be accepted for publication. You may also include additional comments for the author, including concerns about research or publication ethics.

You may also provide optional suggestions and comments to authors that they might find helpful in planning their study.

Reviewer #1: Authors have successfully addressed all the raised comments and updated the manuscript accordingly.

Reviewer #3: Dear editors,

Many thanks for the invitation. The authors have well addressed my concerns. I had no further comment and recommend for its publication.

**********

what does this mean?). If published, this will include your full peer review and any attached files.

If you choose “no”, your identity will remain anonymous but your review may still be made public.

**Do you want your identity to be public for this peer review?** For information about this choice, including consent withdrawal, please see our Privacy Policy

Reviewer #1: No

Reviewer #3: No

**********

Attachment Submitted filename: renamed_26c0b.docx

# Editor report: Acceptance letter

**DOI:** 10.1371/journal.pone.0343078.r006

**Authors:** Ze Xiang

PONE-D-26-05078R2

PLOS One

Dear Dr. Dai,

I'm pleased to inform you that your manuscript has been deemed suitable for publication in PLOS One. Congratulations! Your manuscript is now being handed over to our production team.

At this stage, our production department will prepare your paper for publication. This includes ensuring the following:

* All references, tables, and figures are properly cited

* All relevant supporting information is included in the manuscript submission,

* There are no issues that prevent the paper from being properly typeset

You will receive further instructions from the production team, including instructions on how to review your proof when it is ready. Please keep in mind that we are working through a large volume of accepted articles, so please give us a few days to review your paper and let you know the next and final steps.

Lastly, if your institution or institutions have a press office, please let them know about your upcoming paper now to help maximize its impact. If they'll be preparing press materials, please inform our press team within the next 48 hours. Your manuscript will remain under strict press embargo until 2 pm Eastern Time on the date of publication. For more information, please contact onepress@plos.org.

You will receive an invoice from PLOS for your publication fee after your manuscript has reached the completed accept phase. If you receive an email requesting payment before acceptance or for any other service, this may be a phishing scheme. Learn how to identify phishing emails and protect your accounts at https://explore.plos.org/phishing.

If we can help with anything else, please email us at customercare@plos.org.

Thank you for submitting your work to PLOS One and supporting open access.

Kind regards,

PLOS One Editorial Office Staff

on behalf of

Dr. Ze Xiang

Academic Editor

PLOS One
